# A new electrochemical strategy for the synthesis of a new type of sulfonamide derivatives

**DOI:** 10.1038/s41598-020-74733-2

**Published:** 2020-10-21

**Authors:** Hamed Goljani, Zahra Tavakkoli, Ali Sadatnabi, Mahmood Masoudi-khoram, Davood Nematollahi

**Affiliations:** Faculty of Chemistry, Bu-Ali-Sina University, 65174-38683 Hamedan, Iran

**Keywords:** Electrochemistry, Reaction mechanisms

## Abstract

This study is the first report of electrochemical generation of hydroxyimino-cyclohexa-dien-ylidene haloniums and their application in the synthesis of new halo-*N*-hydroxysulfonamide derivatives. These compounds were obtained in a one-pot process based on the reaction of halonium acceptors with arylsulfinic acids. The method is easy to carry out, as it is performed using the carbon electrodes in a simple undivided cell. The protocol has a broad substrate scope with a tolerance for a variety of functional groups. The proposed mechanism is a ping-pong type reaction mechanism, which in its first stage the halonitroarene is reduced at the cathode to related hydroxylamine and in the second stage the cathodically generated hydroxylamine by oxidation at the anode and participating in disproportionation reaction is converted to the halonium acceptor.

## Introduction

**Figure 1:**
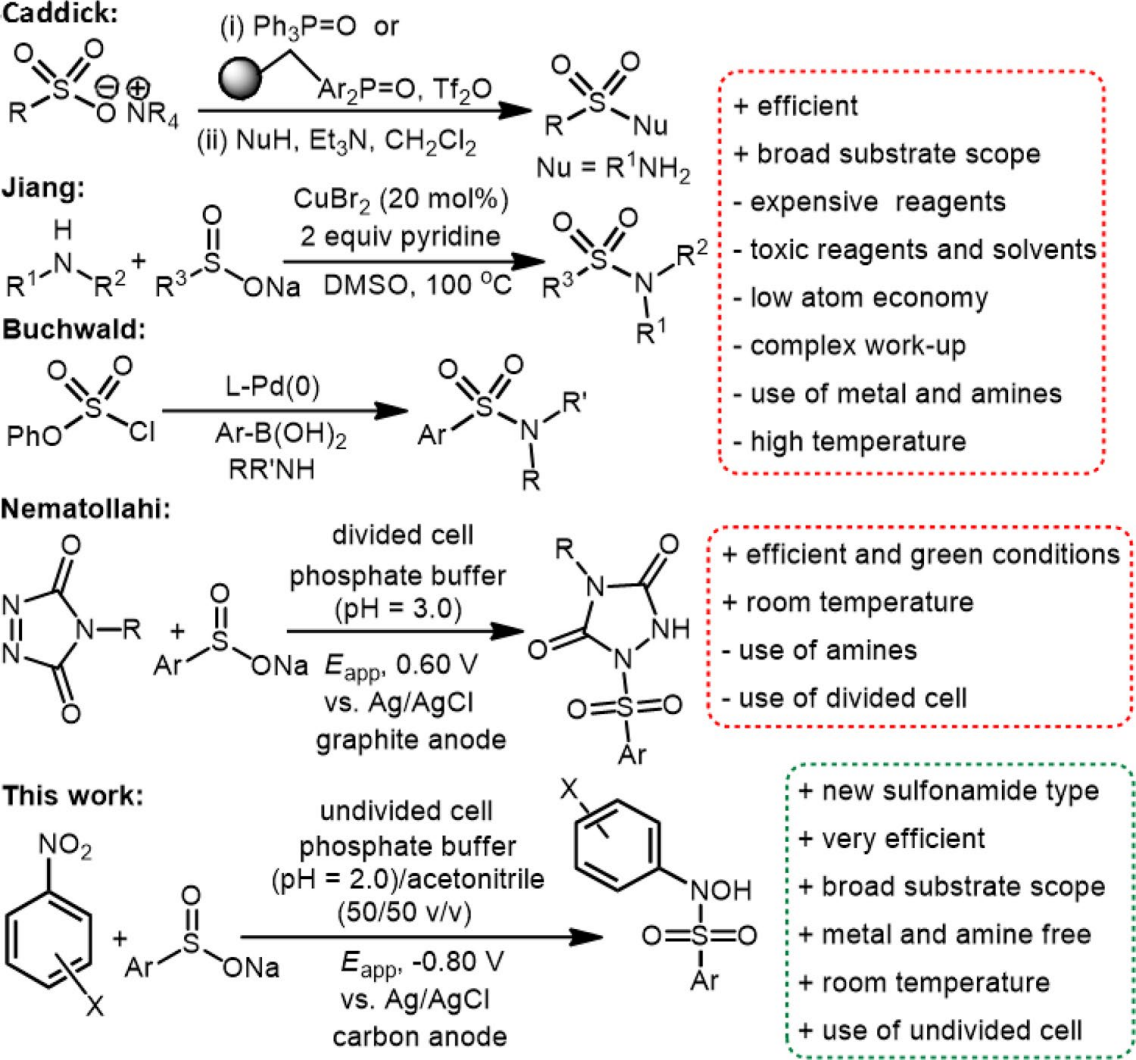
Overview of the synthesis of sulfonamide derivatives. The structures of the compounds were drawn using ChemOffice 12.0 (CambridgeSoft).

Sulfonamides are a well-known and very important class of organic compounds because of their diverse biological and pharmacological activities^1^. These characteristics have developed, numerous strategies for the synthesis of sulfonamide derivatives. The most common method for the synthesis of sulfonamides is direct N–S bond formation^2–10^. In addition, some sulfonamide derivatives were synthesized through electrochemical oxidation of amines in the presence of arylsulfinic acids^11–15^ or through electrochemical oxidative coupling of amines and thiols^16,17^. These are valuable methods, but they have the disadvantage that it uses aromatic amines which are toxic^11–17^. Other methods used in the synthesis of these compounds are the use of metal-catalysts for the synthesis of new sulfonamide derivatives (Fig. 1)^18–26^. These methods does not require the use of amines, but still have disadvantages such as the use of metal-catalysts and harsh workup conditions. From another point of view, to the best of our knowledge, no synthesis has been reported for halo-*N*-hydroxysulfonamide derivatives. This type of sulfonamide (*N*-hydroxysulfonamide) has recently been used as a nitroxyl (HNO) donors in the management of acute decompensated heart failure^27,28^. These data led us to look for an efficient method without these drawbacks for the synthesis of some halo-*N*-hydroxysulfonamide derivatives.

So, we report here the first example of in situ generation and reaction of the unstable hydroxyimino-cyclohexa-dien-ylidenehaloniums intermediates for the successful synthesis of new halo-*N*-hydroxy sulfonamide derivatives based on successive reduction and oxidation of halonitroarenes in the presence of arylsulfinic acids. It should be noted that the toxicity of nitro compounds is lower than similar amines^29^.

On the other hand, aromatic halonitro compounds were widely used in the synthesis of drugs, lumber preservatives, dyes, photographic chemicals, agrochemicals and flavorsm^30^. The first step in the synthesis of these compounds is hydrogenation of aromatic halonitro compounds mainly using transition metal catalysts^31–33^, while we have performed hydrogenation of halonitro compounds electrochemically without using transition metal catalysts. Aromatic halonitro compounds are also attractive because they have a π donor and a σ acceptor halogen atom and also an electron-withdrawing nitro group in their structure. These features as well as the absence of electrochemical data on these compounds, have also led us to understand the electrochemical properties of aromatic halonitro compounds, as another goal of this study. Synthesis of a new class of compounds which has not yet been synthesized electrochemically, in situ generation of the unstable hydroxyimino-cyclohexa-dien-ylidenehaloniums intermediates, for the first time, report of a novel reaction mechanism for the formation of halo-*N*-hydroxysulfonamide compounds, which has not been reported elsewhere and report a sustainable and efficient protocol for the synthesis of a new type of organic compounds are important aspect of this work.

## Materials and methods

A µ-Autolab model PGSTAT 20 potentiostat/galvanostat (Metrohm-Autolab, Netherland) was used for the preparative electrolysis, cyclic voltammetry, differential pulse voltammetry and controlled potential coulometry experiments. The cell system consisted of a glassy carbon disc with diameter of 3.0 mm, a platinum rod and an Ag/AgCl (3 M) were used as working, counter and reference electrodes, respectively. The cathode used in macroscale electrolysis (synthesis) was an assembly of four carbon rods with of 32 cm^2^ area, while the anode consisted of a carbon rod with an area of 8 cm^2^. All electrodes were from AZAR Electrodes, (Iran). *p*-iodonitrobenzene, *o*-iodonitrobenzene, *p*-chloronitrobenzene and *p*-bromonitrobenzene were synthesized according to previously described methods^34,35^. *p*-Toluenesulfinic acid sodium salt (97%), benzensulfinic acid sodium salt (97%), *p*-chlorosulfinic acid sodium salt, acetic acid (99%), perchloric acid (70%) and phosphoric acid (85%) were obtained from Sigma-Aldrich.

### Experimental procedures

#### General procedure for synthesis of 1b–3b, 1c–3c, 1d–3d and 1e–2e

In an undivided cell equipped with four carbon rods as cathode and one carbon rod as anode, a solution (*ca*. 80 mL) of water (phosphate buffer, pH, 2.0, *c* = 0.2 M)/acetonitrile or DMF (50/50 v/v) containing halonitrobenzene (0.5 mM) and arylsulfinic acid (0.5 mM) was electrolyzed at − 0.8 V *vs*. Ag/AgCl. The electrolysis was terminated when the current decayed to 5% of its original value. At the end of the electrolysis, the cell was placed overnight. The precipitated pale yellow was collected by filtration and purified by thin layer chromatography (*n*-hexane/ethyl acetate 4/1).

### Characteristic of the products

*N-Hydroxy-N-(4-iodophenyl) benzenesulfonamide (1b)* Pale yellow; MP: 108–110 °C; ^1^H NMR: *δ* ppm (500 MHz, CD_3_CO-*d*_3_): 6.99 (d, *J* = 10 Hz , 2H, aromatic), 7.56 (m, 4H, aromatic), 7.66 (d, *J* = 10 Hz, 2H, aromatic), 7.73 (m, 1H, aromatic), 10.21 (s, 1H, OH); ^13^C NMR: *δ* ppm (125 MHz, CD_3_CO-*d*_3_: 91.2, 124.5, 128.6 , 129.5, 132.8, 134.0, 137.3, 143.0; IR (KBr) (cm^-1^): 3334 (medium, O–H), 1477 (medium C=C), 1340 and 1165 (strong, S=O), 1084, 999, 685, 593, 572; MS (*m/z*) (EI, 70 eV) (relative intensity): 218 (100), 77 (100), 359 (80), 141 (50), 374 (M, 25).

*N-Hydroxy-N-(4-iodophenyl)-4-methylbenzenesulfonamide (2b)* Pale yellow; MP: 157–158 °C; ^1^H NMR: *δ* ppm (500 MHz, CD_3_CO-*d*_3_): 2.42 (s, 3H, methyl), 6.99 (d, *J* = 10, 2H, aromatic), 7.35 (d, *J* = 10 Hz, 2H, aromatic), 7.43 (d, *J* = 10 Hz, 2H, aromatic), 7.66 (d, *J* = 10 Hz, 2H, aromatic), 10.30 (s, 1H, OH); ^13^C NMR: *δ* ppm (125 MHz, CD_3_CO-*d*_3_: 20.7, 91.0, 124.5, 128.6 , 129.1, 129.5, 130.0, 137.2, 138.1, 143.1, 145.0; IR (KBr) (cm^−1^): 3351 (medium, O–H), 1596 (medium C=C), 1475, 1337 and 1161 (strong, S=O), 1088, 990, 713, 664, 586, 548; MS (*m/z*) (EI, 70 eV) (relative intensity): 91 (100), 155 (85), 262 (25), 139 (35), 388 (M-1, 40).

*4-Chloro-N-hydroxy-N-(4-iodophenyl)benzenesulfonamide (3b)* Pale yellow; MP: 154–156 °C; ^1^H NMR: *δ* ppm (500 MHz, CD_3_CO-*d*_3_): 7.01 (d, *J* = 10, 2H, aromatic), 7.55 (d, *J* = 10 Hz, 2H, aromatic), 7.61 (d, *J* = 10 Hz, 2H, aromatic), 7.68 (d, *J* = 10 Hz, 2H, aromatic), 10.38 (s, 1H, OH); ^13^C NMR: *δ* ppm (125 MHz, CD_3_CO-*d*_3_: 91.5, 124.6, 124.9 , 128.9, 131.2, 137.4, 138.9, 140.0, 142.6; IR (KBr) (cm^−1^): 3357 (medium, O–H), 1571 (medium C=C), 1476, 1344 and 1165 (strong, S=O), 1091, 992, 829, 759, 620, 557; MS (*m/z*) (EI, 70 eV) (relative intensity): 111 (100), 175 (90), 282 (25), 393 (15), 408 (M-1, 40).

*N-(4-Chlorophenyl)-N-hydroxybenzenesulfonamide (1c)* Pale yellow; MP: 102–104 °C; ^1^H NMR: *δ* ppm (500 MHz, CD_3_CO-*d*_3_): 7.18 (d, *J* = 10 Hz , 2H, aromatic), 7.33 (d, *J* = 10, 2H, aromatic), 7.57 (m, 4H, aromatic), 7.73 (m, 1H, aromatic), 10.29 (s, 1H, OH); ^13^C NMR: *δ* ppm (125 MHz, CD_3_CO-*d*_3_: 124.2, 128.2 , 128.5, 129.5, 132.0, 132.8, 134.0, 141.8; IR (KBr) (cm^−1^): 3335 (medium, O–H), 1584 (medium C=C), 1481, 1348 and 1179 (strong, S=O), 1087, 1015, 831, 737, 608, 559, 577; MS (*m/z*) (EI, 70 eV) (relative intensity): 142 (100), 77 (70), 111 (45), 267 (10), 283 (M, 15).

*N-(4-Chlorophenyl)-N-hydroxy-4-methylbenzenesulfonamide (2c)* Pale yellow; MP: 129–130 °C; ^1^H NMR: *δ* ppm (500 MHz, CD_3_CO-*d*_3_): 2.42 (S, 3H, methyl), 7.19 (d, *J* = 10, 2H, aromatic), 7.32 (d, *J* = 10 Hz, 2H, aromatic), 7.35 (d, *J* = 10 Hz, 2H, aromatic), 7.43 (d, *J* = 10 Hz, 2H, aromatic), 10.23 (s, 1H, OH); ^13^C NMR: *δ* ppm (125 MHz, CD_3_CO-*d*_3_: 20.5, 124.1, 128.2 , 129.1, 129.5, 129.9, 131.8, 141.9, 145.0; IR (KBr) (cm^−1^): 3346 (medium, O–H), 2926 (weak, CH_3_) 1595 (medium C=C), 1482, 1342 and 1163 (strong, S=O), 1086, 889, 831, 814, 731, 671, 555; MS (*m/z*) (EI, 70 eV) (relative intensity): 91 (100), 142 (90), 111 (60), 65 (60), 281(30), 297 (M-1, 15).

*4-Chloro-N-(4-chlorophenyl)-N-hydroxybenzenesulfonamide (3c)* Pale yellow; MP: 142–146 °C; ^1^H NMR: *δ* ppm (500 MHz, CD_3_CO-*d*_3_): 7.20 (d, *J* = 10, 2H, aromatic), 7.35 (d, *J* = 10 Hz, 2H, aromatic), 7.55 (d, *J* = 10 Hz, 2H, aromatic), 7.61 (d, *J* = 10 Hz, 2H, aromatic), 10.39 (s, 1H, OH); ^13^C NMR: *δ* ppm (125 MHz, CD_3_CO-*d*_3_: 124.2, 128.4, 128.9 , 131.2, 131.4, 132.2, 140.0, 141.5; IR (KBr) (cm^−1^): 3349 (medium, O–H), 1574 (medium C=C), 1482, 1350 and 1182 (strong, S=O), 1090, 1013, 834, 760, 638, 589, 562; MS (*m/z*) (EI, 70 eV) (relative intensity): 111 (100), 175 (85), 142 (25), 301 (10), 316 (M-1, 20).

*N-Hydroxy-N-(2-iodophenyl)benzenesulfonamide (1d)* Pale yellow; MP: 142–144 °C; ^1^H NMR: *δ* ppm (500 MHz, CD_3_CO-*d*_3_): 6.78 (d, *J* = 10 Hz , 1H, aromatic), 7.11 (t, 1H, aromatic), 7.30 (t, 1H, aromatic), 7.68 (m, 2H, aromatic), 7.81 (m, 3H, aromatic), 7.98 (d, J = 10 Hz, 1H, aromatic), 10.19 (s, 1H, OH); ^13^C NMR: *δ* ppm (125 MHz, CD_3_CO-*d*_3_: 99.0, 126.3, 128.5 , 128.7, 130.1, 130.3, 134.3, 139.6, 144.5; IR (KBr) (cm^−1^): 3329 (medium, O–H), 1451 (medium C=C), 1350 and 1175 (strong, S=O), 1087, 908, 767, 692, 571; MS (*m/z*) (EI, 70 eV) (relative intensity): 224 (100), 203 (80), 218 (40), 359 (40), 375 (M, 25).

*N-Hydroxy-N-(2-iodophenyl)-4-methylbenzenesulfonamide (2d)* Pale yellow; MP: 162–164 °C;^1^H NMR: *δ* ppm (500 MHz, CD_3_CO-*d*_3_): 2.49 (S, 3H, methyl), 7.1 (t, 1H, aromatic), 7.29 (t, 1H, aromatic), 7.45 (d, *J* = 10 Hz, 2H, aromatic), 7.68 (d, *J* = 10 Hz, 2H, aromatic), 7.94 (d, *J* = 10 Hz, 2H, aromatic), 10.19 (s, 1H, OH); ^13^C NMR: *δ* ppm (125 MHz, CD_3_CO-*d*_3_: 99.0, 126.2, 128.5 , 129.2, 130.2, 131.3, 139.5, 144.6, 145.1; IR (KBr) (cm^−1^): 3337 (medium, O–H), 1463 (medium C=C), 1344 and 1165 (strong, S=O), 1087, 769, 710, 665, 577; MS (*m/z*) (EI, 70 eV) (relative intensity): 223 (100), 203 (60), 91 (70), 373 (20), 389 (M, 25).

*4-Chloro-N-hydroxy-N-(2-iodophenyl)benzenesulfonamide (3d)* Pale yellow; MP: 154–156 °C; ^1^H NMR: *δ* ppm (500 MHz, CD_3_CO-*d*_3_): 6.84 (d, *J* = 10 Hz , 1H, aromatic), 7.13 (t, 1H, aromatic), 7.34 (t, 1H, aromatic), 7.71 (d, J = 10, 2H, aromatic), 7.81 (d, J = 10, 2H, aromatic), 7.96 (d, *J* = 10 Hz , 1H, aromatic), 10.19 (s, 1H, OH); ^13^C NMR: *δ* ppm (125 MHz, CD_3_CO-*d*_3_): 99.0, 126.1, 128.7 , 129.0, 130.4, 131.8, 132.8, 139.6, 140.1, 144.2; IR (KBr) (cm^−1^): 3350 (medium, O–H), 1464 (medium C=C), 1348 and 1172 (strong, S=O), 1091, 764, 707, 640, 574; MS (*m/z*) (EI, 70 eV) (relative intensity): 223 (100), 203 (75), 111(60), 393 (20), 409 (M, 15).

*N-(4-Bromophenyl)-N-hydroxybenzenesulfonamide (1e)* Pale yellow; MP: 92–94 °C; ^1^H NMR: *δ* ppm (500 MHz, CD_3_CO-*d*_3_): 7.14 (d, *J* = 10 Hz , 2H, aromatic), 7.51 (d, *J* = 10 Hz, 2H, aromatic), 7.58 (m, 4H, aromatic),7.75 (m, 1H, aromatic), 10.31 (s, 1H, OH); ^13^C NMR: *δ* ppm (125 MHz, CD_3_CO-*d*_3_): 119.9, 124.5, 128.6, 129.5, 131.3, 132.8, 134.0, 142.3; IR (KBr) (cm^−1^): 3341 (medium, O–H), 1480 (medium C=C), 1342 and 1163 (strong, S=O), 1069, 886, 720, 667, 591; MS (*m/z*) (EI, 70 eV) (relative intensity): 77 (100), 141 (40), 51 (50), 311 (10), 326 (M, 25).

*N-(4-Bromophenyl)-N-hydroxy-4-methylbenzenesulfonamide (2e)* Pale yellow; MP: 113–114 °C; ^1^H NMR: *δ* ppm (500 MHz, CD_3_CO-*d*_3_): 2.44 (S, 3H, methyl), 7.15 (d, *J* = 10, 2H, aromatic), 7.37 (d, *J* = 10 Hz, 2H, aromatic), 7.45 (d, *J* = 10 Hz, 2H, aromatic), 7.49 (d, *J* = 10 Hz, 2H, aromatic), 10.24 (s, 1H, OH);^13^C NMR: *δ* ppm (125 MHz, CD_3_CO-*d*_3_: 21.0, 119.7, 124.4, 129.2, 129.6, 129.9, 131.2, 142.8, 145.0; IR (KBr) (cm^−1^): 3341 (medium, O–H), 1480 (medium C=C), 1342 and 1163 (strong, S=O), 1069, 987, 667, 591, 553; MS (*m/z*) (EI, 70 eV) (relative intensity): 91 (100), 155 (80), 187 (60), 327 (50), 343 (M, 25).

## Results and discussion

**Figure 2:**
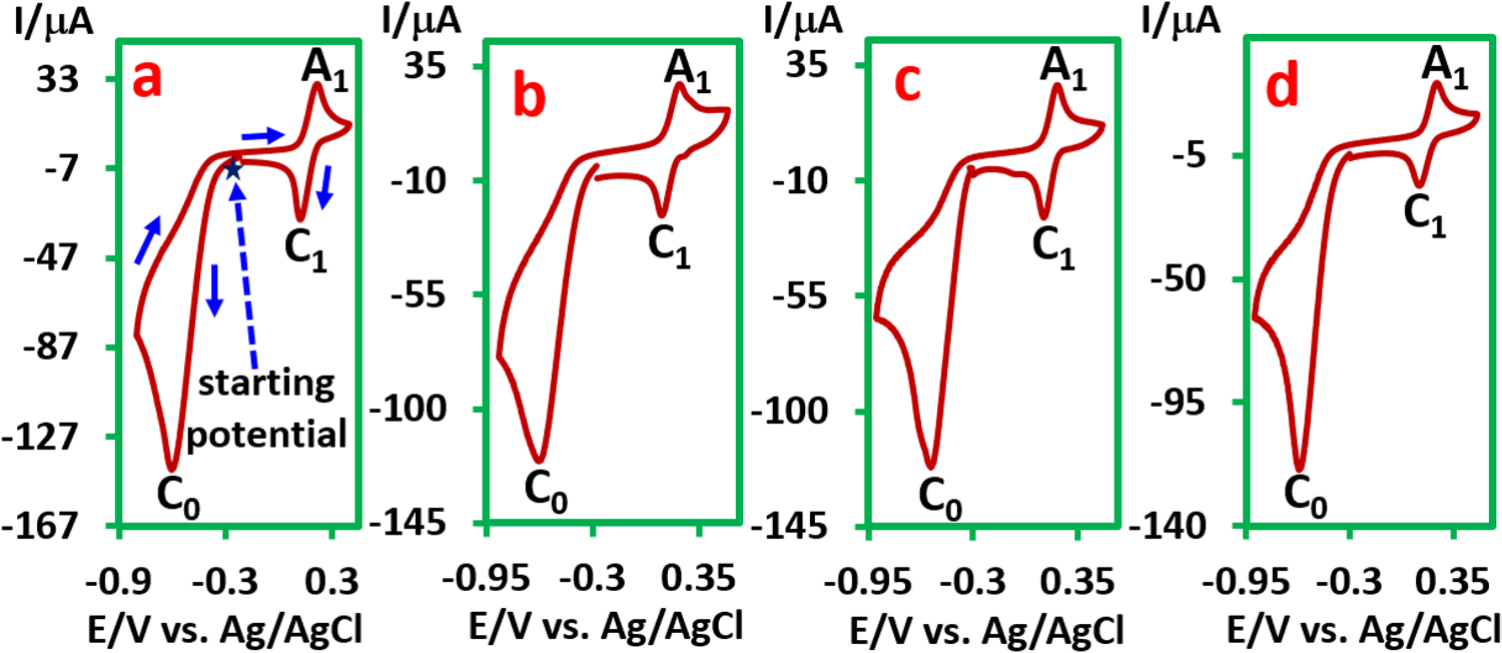
Cyclic voltammograms of 1.0 mM (**a**) **PINB**, (**b**) **PCNB**, (**c**) **PBNB** and (**d**) **OINB** in aqueous phosphate buffer (pH, 2.0, *c* = 0.2 M)/acetonitrile (50/50 v/v) at GC electrode. Scan rate: 100 mV/s at room temperature. This figure was prepared by Microsoft Excel (OFFICE 2013).

Cyclic voltammograms (CVs) of *p*-iodonitrobenzene (**PINB**), *p*-chloronitrobenzene (**PCNB**), *p*-bromonitrobenzene (**PBNB**) and *o*-iodonitrobenzene (**OINB**) in aqueous phosphate buffer (pH, 2.0, *c* = 0.2 M)/acetonitrile (50/50 v/v) are shown in Fig. 2. The voltammograms consist of an irreversible cathodic peak (C_0_) which is correspond to a four-electron reduction of nitro group to hydroxylamine group^36–39^ and a reversible couple (A_1_/C_1_), at a more positive potential.

**Figure 3:**
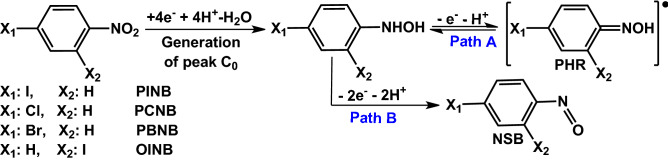
Electrochemical reactions of halonitroarenes. The structures of the compounds were drawn using ChemOffice 12.0 (CambridgeSoft).

The redox behavior of A_1_/C_1_ is shown in Fig. 3. Two pathways are possible for the oxidation of cathodically generated phenylhydroxylamine; (1) one-electron oxidation and the formation of phenylhydroxylamine radical (**PHR**) (path A) and (2) two-electron oxidation and the formation of nitrosobenzene derivatives (**NSB**) (path B).

In order to identify the more probable oxidation pathway, the number of electrons exchanged in the oxidation of *N*-(4-iodophenyl)hydroxylamine was calculated using differential pulse voltammetry (DPV) method (Supplementary Fig. S1), by measuring half-peak width (*W*_1/2_) according to equation^40^:

$$ W_{{{1}/{2}}} = { 3}.{52}RT/nF. $$

**Figure 4:**
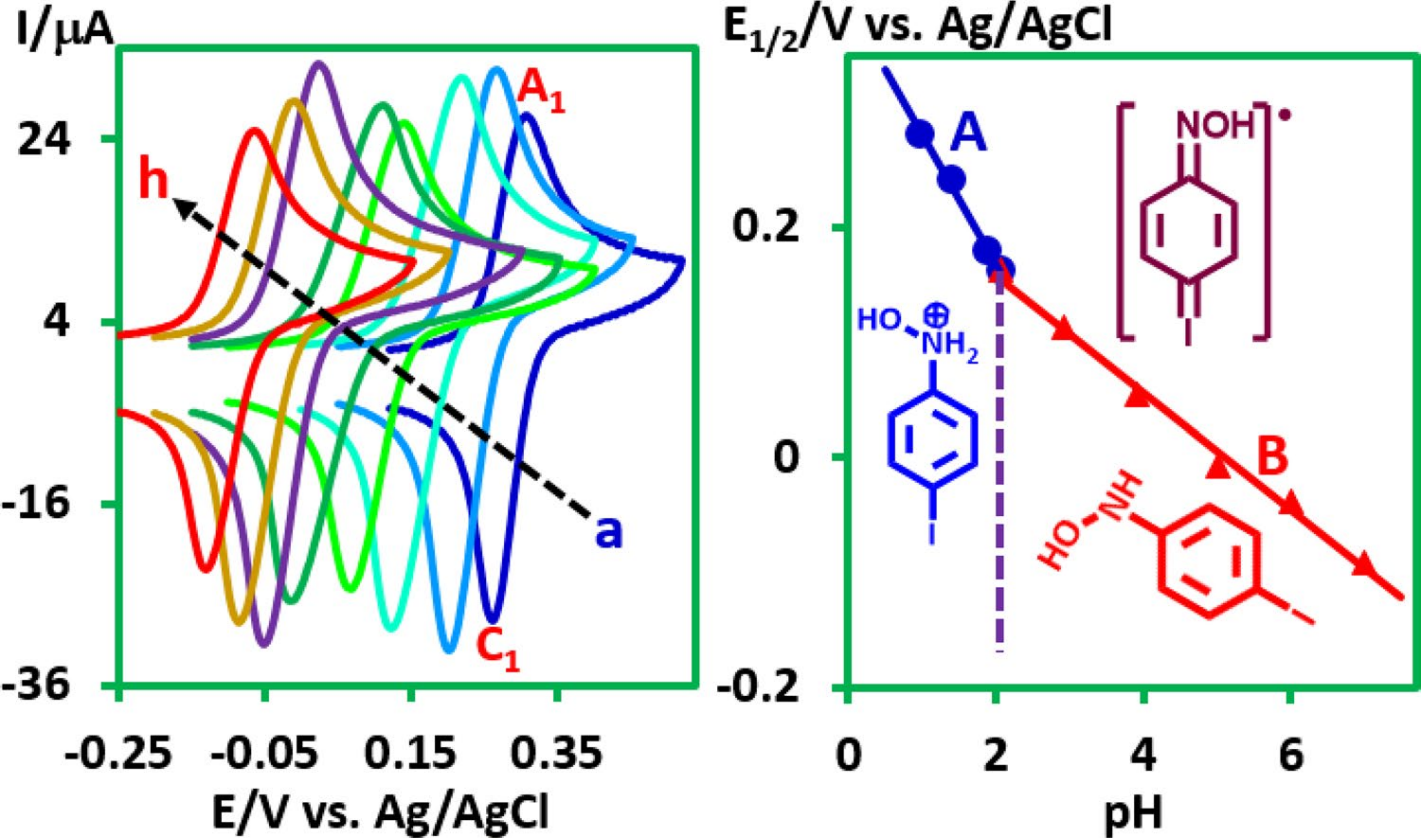
Left: cyclic voltammograms of **PINB** (1.0 mM) in buffer solution with various pH values/acetonitrile (50/50, v/v) mixture. pH values from a to h are: 0.97, 1.40, 1.87, 2.93, 3.90, 5.00, 6.00 and 7.00. Scan rate: 10 mV/s, at glassy carbon electrode, Temperature, 25 ± 1 °C. Right: Pourbaix diagram for **PINB**. This figure was prepared by Microsoft Excel (OFFICE 2013).

**Figure 5:**
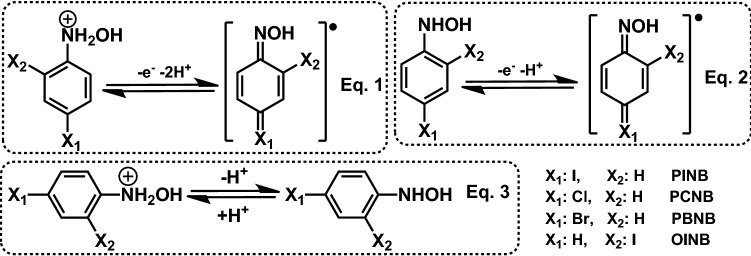
Oxidation/reduction and acid/base behaviors of halophenylhydroxylamines at different pH values. The structures of the compounds were drawn using ChemOffice 12.0 (CambridgeSoft).

The result of this experiment is in agreement with the one-electron transfer in the oxidation step of *N*-(4-iodophenyl)hydroxylamine and confirms the formation of phenylhydroxylamine radical (**PHR**) under our experimental conditions. The structures of **PHR** and **NSB** were optimized at B3LYP/6-311G level of theory by using guassian09W (Supplementary Fig. S2). The relative Gibbs free energies of **PHR** and **NSB** were found to be 15.1 and 0.0 kcal/mol, respectively, which is another confirmation of the formation of **PHR**. The effect of solution pH on the half-wave potential (*E*_1/2_) of *N*-(4-iodophenyl)hydroxylamine (**PIPHA**) and other phenyl hydroxylamines produced from the reduction of **PCNB**, **PBNB**, and **OINB** was evaluated in the range of 1–7 (Fig. 4 and Supplementary Figs. S3–S5). It was found that half-wave potential (*E*_1/2_) of **PINB** shifted to negative values with increasing pH which indicates that proton is involved in the electrode process. The Pourbaix diagram of **PIPHA** is also shown in Fig. 4. It consists of two lines with slopes 113 (line A) and 52 (line B) mV/pH (Fig. 5). The slope of 113 mV/pH is consistent with the theoretical value for a one-electron/two-proton process (118 mV/pH). On the other hand, the slope of 52 mV/pH (line B) is in accordance with the one-electron/one-proton process (59 mV/pH).

Also, the pK_a_ values of protonated **PIPHA**, **PCPHA**, **PBPHA** and **OIPHA** are 2.06, 1.97, 1.97 and 2.10, respectively. Details for other phenylhydroxylamines are shown in Supplementary Figs. S3–S5.

**Figure 6:**
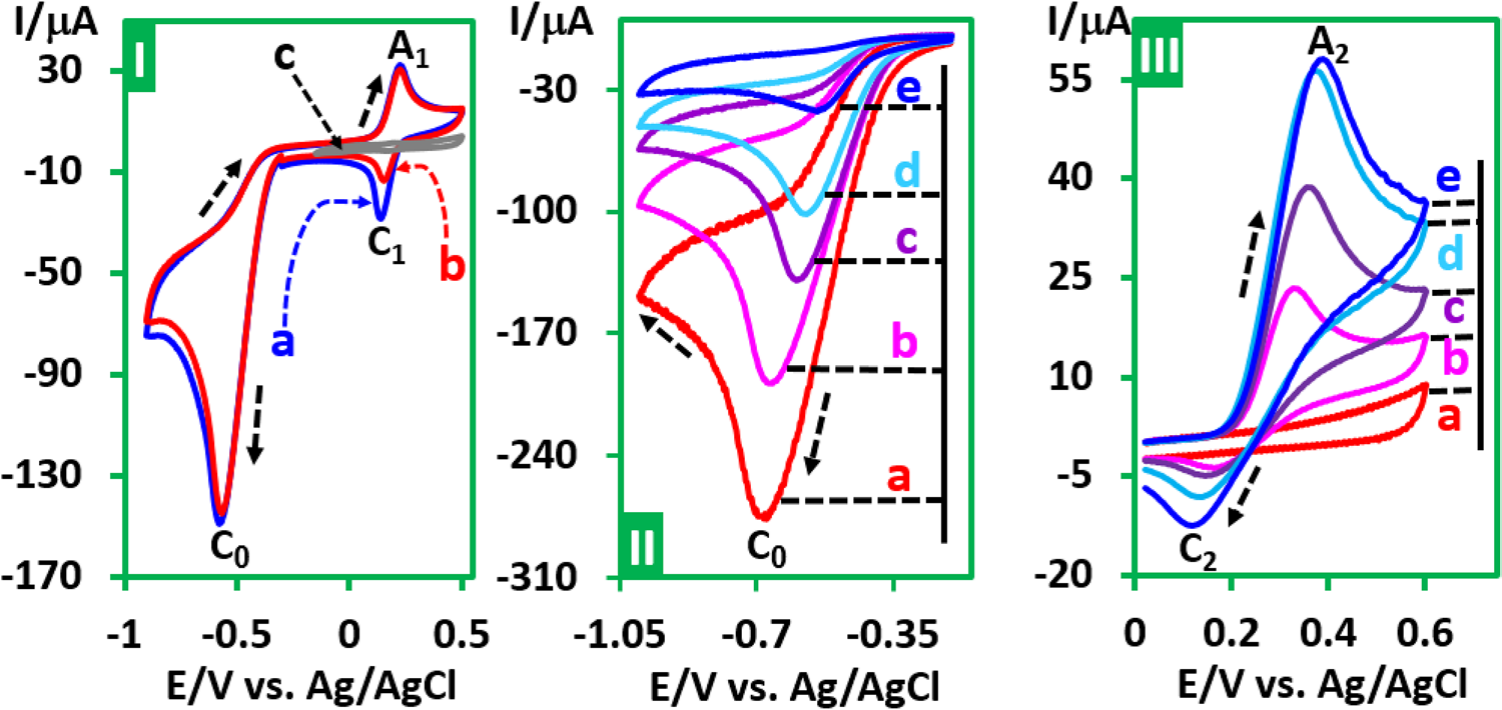
Part I: Cyclic voltammograms of **PINB** (1.0 mM): (**a**) in the absence, (**b**) in the presence of **BSA** (1.0 mM) and (**c**) cyclic voltammogram of **BSA** (1.0 mM). Scan rate: 10 mV/s. Part II. Cyclic voltammograms of **PINB** (0.5 mmol) in the presence of **BSA** (0.5 mmol) during controlled-potential coulometry at − 0.8 V vs. Ag/AgCl. Scan rate: 100 mV/s .Part III: Similar to part II, but when scanning the potential from 0.0 to + 0.60 V. Voltammograms in parts II and III are taken after consumption of: (**a**) 0, (**b**) 50, (**c**) 100, (**d**) 150 and (**e**) 200 C. Solvent: water (phosphate buffer, pH, 2.0, *c* = 0.2 M)/acetonitrile (50/50 v/v) at room temperature. Working electrode: GC electrode. This figure was prepared by Microsoft Excel (OFFICE 2013).

The cyclic voltammogram of **PINB** in the presence of benzenesulfinic acid (**BSA**) as a nucleophile is shown in Fig. 6, part I, curve b. Comparison of this voltammogram with that of in the absence of **BSA** shows that *I*_pC1_ decreases significantly. This decrease confirms the reaction between oxidized hydroxylamine and **BSA**.

Controlled-potential coulometry was performed in a solution containing **PINB** (0.5 mmol) and **BSA** (0.5 mmol) at − 0.8 V versus Ag/AgCl. The electrolysis progress was monitored by cyclic voltammetry (Fig. 6, parts II and III). Part II shows a continuous decrease in cathodic peak C_0_ current over the charge passed due to the reduction of the nitro group to hydroxylamine. On the other hand, part III shows a continuous increase of the peaks A_2_ and C_2_ currents with increasing amounts of charge.

**Figure 7:**
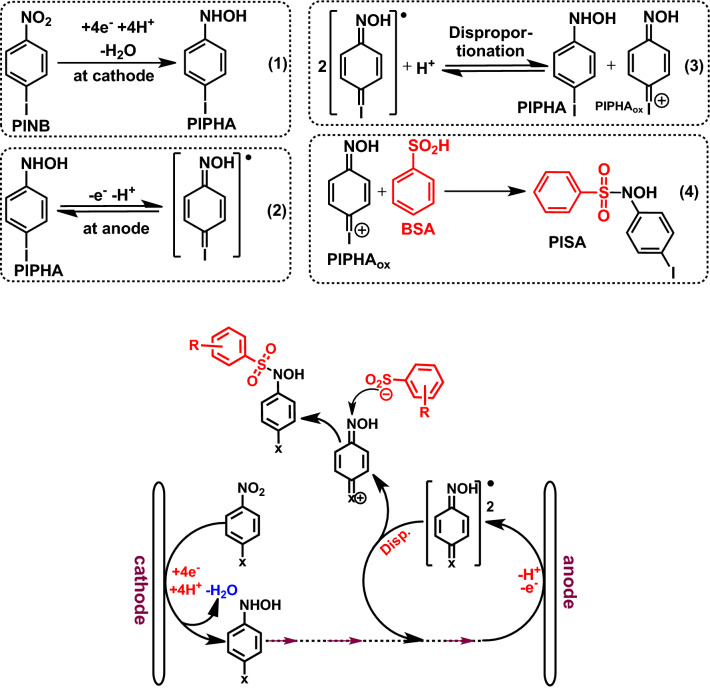
Electrochemical reaction mechanism of **PINB** in the presence of **BSA**. The structures of the compounds were drawn using ChemOffice 12.0 (CambridgeSoft).

The proposed mechanism for the oxidation of **PINB** in the presence of **BSA** is shown in Fig. 7. According to this mechanism, cathodically generated **PIPHA** at the anode surface is oxidized to its corresponding radical. The disproportionation of the **PIPHA** radicals in the next step, to yield the starting **PIPHA** and 4-(hydroxyimino)cyclohexa-2,5-dien-1-ylidene) iodonium (**PIPHA**_**ox**_). The reaction of **PIPHA**_**ox**_ as an acceptor with **BSA** (R = H) affording the corresponding iodosulfonamide (**PISA**).

The optimization of conditions for the electrochemical synthesis of **PISA** by the change in the electrode material, solvent and aqueous solution pH was studied and the results are shown in Table 1. **PISA** was achieved in 75% yield with carbon electrodes in aqueous phosphate buffer (pH, 2.0, *c* = 0.2 M)/acetonitrile mixture (50/50 v/v) (entry 1). DMF as a co-solvent also afforded the product in 86% yield (entry 4). We think that pH is the most important factor for increasing yield, by preventing or minimizing side reactions such as **PIPHA**–**PIPHA**_**ox**_ coupling reaction. At pH 2, **PIPHA** is mostly protonated (Fig. 2) and cannot act as an effective nucleophile to compete with the reaction of **BSA** with **PIPHA**_**ox**_. At pH 2, the use of electrodes other than carbon and solvents other than DMF, reduce yield. With the optimized conditions in hand, we investigated the scope of the reaction using a range of halonitroarenes and sulfinic acids (Table 2). The data presented in Table 2 show that we were able to develop an efficient method to synthesize new halosulfonamides in a one-pot reaction with a 63–86% overall yield.

**Table 1:** Optimization of conditions for the synthesis of **PISA**.


Entry	Anode	Cathode	Organic solvent	Aqueous solution pH^a^	Yield (%)^c^
1	Carbon	Carbon	CH_3_CN	Phosphate Buf. (pH, 2.0)	75
2	Carbon	Carbon	CH_3_CN	Phosphate Buf. (pH, 3.0)	71
3	Carbon	Carbon	CH_3_CN	Phosphate Buf. (pH, 4.0)	52
4	Carbon	Carbon	DMF	Phosphate Buf. (pH, 2.0)	86
5	Carbon	Cu	CH_3_CN	Phosphate Buf. (pH, 2.0)	59
6	Carbon	Fe	CH_3_CN	Phosphate Buf. (pH, 2.0)	65
7	Carbon	SS^b^	CH_3_CN	Phosphate Buf. (pH, 2.0)	52
8	Carbon	Carbon	CH_3_CN	HClO_4_ (0.1 M)	68
9	SS	Carbon	CH_3_CN	Phosphate Buf. (pH, 2.0)	34

The structures of the compounds were drawn using ChemOffice 12.0 (CambridgeSoft).

^a^Phosphate buffer concentration: 0.2 M.

^b^Stainless steel.

^c^Yields are calculated based on the weight of isolated products after work-up.

**Table 2:**
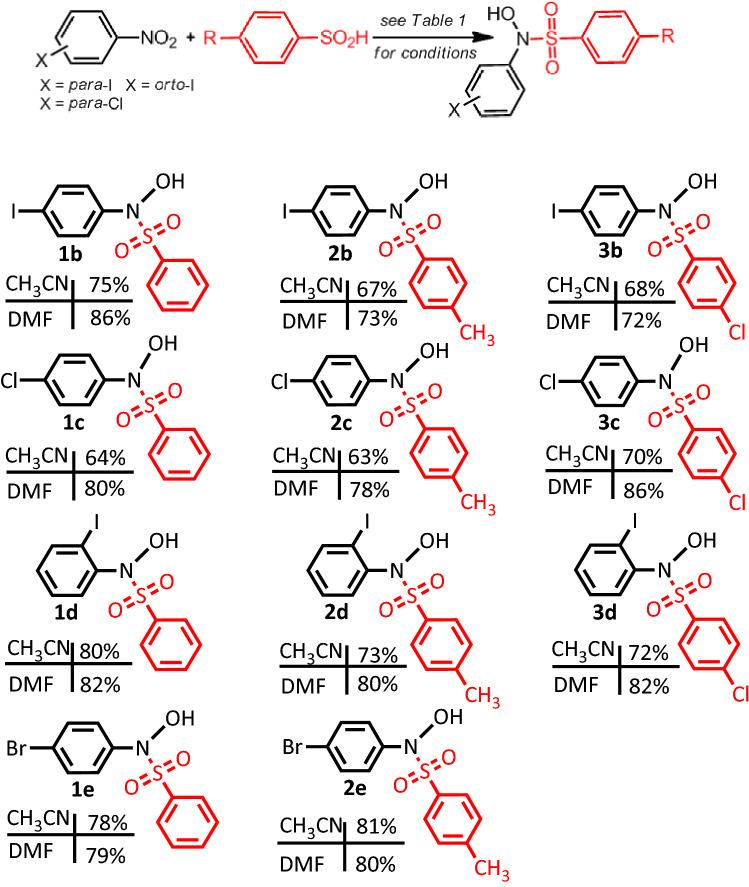
Scope of the halosulfonamide synthesis.

In an undivided cell equipped with four carbon rods as cathode and one carbon rod as anode, a solution (*ca*. 80 mL) of water (phosphate buffer, pH, 2.0, *c* = 0.2 M)/acetonitrile(50/50 v/v) containing halonitrobenzene (0.5 mM) and arylsulfinic acid (0.5 mM) was electrolyzed at − 0.8 V vs. Ag/AgCl. The structures of the compounds were drawn using ChemOffice 12.0 (CambridgeSoft).

As shown in Table 2, a number of halo-*N*-hydroxy sulfonamides have been synthesized in a one-pot process for the first time. No data have been reported so far for the electrochemically synthesis of these types of sulfonamides. The reaction is carried out under mild conditions at room temperature and atmospheric pressure with a high atom economy and without using inorganic (or organic) oxidizing agents or catalysts. Selective electrohydrogenation of halonitroarenes (without removal of halogen) is another achievement of this method. Compared with the literature procedures (Supplementary Table S1) that require amines, metal catalysts, high temperatures, expensive, unstable and toxic reagents, this method operates under mild and sustainable reaction conditions and utilizes low-cost materials which is suitable for scalable production.

## Conclusions

In this study, a ping pong reaction mechanism is proposed, which in its first stage the halonitroarene is reduced at the cathode to related hydroxylamine and then the oxidation of cathodically generated hydroxylamine along with the disproportionation reaction to form the halonium acceptor. The reaction of halonium compound with arylsulfinic acids affording the corresponding halo-*N*-hydroxysulfonamide. In this new strategy, we utilized the primary electrochemical principles for the synthesis of a new type of sulfonamids. These compounds have not been synthesized electrochemically so far and therefore, we were interested in the synthesis of them. In this paper, we also reported the first example of in situ generation and reaction of the unstable hydroxyimino-cyclohexa-dien-ylidenehaloniums intermediates for the synthesis of organic compounds. This method has several unique features which are: (1) Synthesis of halo-*N*-hydroxysulfonamide derivatives. This class of compounds had not yet been synthesized electrochemically. (2) This is the first example of in situ generation of the unstable hydroxyimino-cyclohexa-dien-ylidenehaloniums intermediates without using any metal catalyst and electrochemical detection of them. (3) The reaction mechanism reported for the synthesis of halo-*N*-hydroxysulfonamide compounds (Fig. 7), including cathodic reduction, anodic oxidation, disproportionation, and addition reaction is unique and it has not been reported elsewhere. (5) The electrochemical data reported for halonitrobenzenes, **PINB**, **PCNB**, **PBNB** and **OINB** and also electrogenerated *N*-(halophenyl)hydroxylamines, **PIPHA**, **PCPHA**, **PBPHA** and **OIPHA**, are unique and they have not been reported elsewhere. (6) This protocol describes a detailed procedure for the formation of haloniums intermediates and provides a novel approach for the synthesis of halo-*N*-hydroxysulfonamide derivatives in sustainable and facile conditions.

## Supplementary information

Supplementary Information.
