# Supplementary material for: A new electrochemical strategy for the synthesis of a new type of sulfonamide derivatives
Source: Sci Rep. 2020 Oct 21;10:17904. doi: 10.1038/s41598-020-74733-2 (PMC7577992; doi:10.1038/s41598-020-74733-2)

Supplementary Information

A new Electrochemical Strategy for the Synthesis of a new Type of Sulfonamide Derivatives

Hamed Goljani, Zahra Tavakkoli, Ali Sadatnabi,[] Mahmoud Masoudi-khoram and Davood Nematollahi*

Faculty of Chemistry, Bu-Ali-Sina University, Hamedan 65174, Iran.

nemat@basu.ac.ir.

Table of Contents:

1 Table S1…… Page 5

2 Differential pulse voltammogram of **PINB** …… Page 6

3 Optimized structure of **NSB** and **PHI**…… Page 7

4 Cyclic voltammograms of **OINB** at different pH values…… Page 8

5 Cyclic voltammograms of **PCNB** at different pH values…… Page 9

6 Cyclic voltammograms of **PBNB** at different pH values…… Page 10

7 Oxidation potential and E_{HOMO} of *N*-(4 or 2-halophenyl)hydroxylamine…… Page 11

8 HOMO orbitals structure of *N*-(4 or 2-halophenyl)hydroxylamine…… Page 12

9 Procedure for synthesis of halo-*N*-hydroxysulfonamide derivatives…… Page 13

10 Characterization of products…… Page 14

11 FT-IR spectrum of **1b**…… Page 19

12 ^{1}H NMR spectrum of **1b**…… Page 20

13 Expanded ^{1}H NMR spectrum of **1b**…… Page 21

14 ^{13}C NMR spectrum of **1b**…… Page 22

15 Expanded ^{13}C NMR spectrum of **1b**…… Page 23

16 MS spectrum of **1b**…… Page 24

17 FT-IR spectrum of **2b**…… Page 25

18 ^{1}H NMR spectrum of **2b**…… Page 26

19 Expanded ^{1}H NMR spectrum of **2b**…… Page 27

20 ^{13}C NMR spectrum of **2b**…… Page 28

21 Expanded ^{13}C NMR spectrum of **2b**…… Page 29

22 MS spectrum of **2b**…… Page 30

23 FT-IR spectrum of **3b**…… Page 31

24 ^{1}H NMR spectrum of **3b**…… Page 32

25 Expanded ^{1}H NMR spectrum of **3b**…… Page 33

26 ^{13}C NMR spectrum of **3b**…… Page 34

27 Expanded ^{13}C NMR spectrum of **3b**…… Page 35

28 MS spectrum of **3b**…… Page 36

29	FT-IR spectrum of **1c**…………………………………………………………..	Page 37
30	^{1}H NMR spectrum of **1c**……………………………………………………...	Page 38
31	Expanded ^{1}H NMR spectrum of **1c**………………………………………………	Page 39
32	^{13}C NMR spectrum of **1c**……………………………………………………….	Page 40
33	Expanded ^{13}C NMR spectrum of **1c**……………………………………………..	Page 41
34	MS spectrum of **1c**……………………………………………………………...	Page 42
35	FT-IR spectrum of **2c**…………………………………………………………...	Page 43
36	^{1}H NMR spectrum of **2c**………………………..……………………………….	Page 44
37	Expanded ^{1}H NMR spectrum of **2c**………………………………………………	Page 45
38	^{13}C NMR spectrum of **2c**……………………………………………………….	Page 46
39	Expanded ^{13}C NMR spectrum of **2c**……………………………………………..	Page 47
40	MS spectrum of **2c**……………………………………………………………...	Page 48
41	FT-IR spectrum of **3c**…………………………………………………………...	Page 49
42	^{1}H NMR spectrum of **3c**……………………………………………………...	Page 50
43	Expanded ^{1}H NMR spectrum of **3c**………………………………………………	Page 51
44	^{13}C NMR spectrum of **3c**……………………………………………………….	Page 52
45	Expanded ^{13}C NMR spectrum of **3c**……………………………………………..	Page 53
46	MS spectrum of **3c**………………………………………...………………………	Page 54
47	FT-IR spectrum of **1d**…………………………………………………………...	Page 55
48	^{1}H NMR spectrum of **1d**……………………………………………………..	Page 56
49	Expanded ^{1}H NMR spectrum of **1d**………………………………………………	Page 57
50	^{13}C NMR spectrum of **1d**……………………………………………………….	Page 58
51	Expanded ^{13}C NMR spectrum of **1d**……………………………………………..	Page 59
52	MS spectrum of **1d**……………………………………………………………...	Page 60
53	FT-IR spectrum of **2d**…………………………………………………………...	Page 61
54	^{1}H NMR spectrum of **2d**……………………………………………………..	Page 62
55	Expanded ^{1}H NMR spectrum of **2d**………………………………………………	Page 63
56	^{13}C NMR spectrum of **2d**……………………………………………………….	Page 64
57	Expanded ^{13}C NMR spectrum of **2d**……………………………………………..	Page 65

58 MS spectrum of **2d**…………………………………………………………... Page 66
59 FT-IR spectrum of **3d**………………………………………………………... Page 67
60 ^{1}H NMR spectrum of **3d** Page 68
61 Expanded ^{1}H NMR spectrum of **3d**……………………………………… Page 69
62 ^{13}C NMR spectrum of **3d**………………………………………………… Page 70
63 Expanded ^{13}C NMR spectrum of **3d**…………………………………….. Page 71
64 MS spectrum of **3d**…………………………………………………………... Page 72
65 FT-IR spectrum of **1e**………………………………………………………... Page 73
66 ^{1}H NMR spectrum of 1e Page 74
67 Expanded ^{1}H NMR spectrum of **1e**……………………………………… Page 75
68 ^{13}C NMR spectrum of **1e**………………………………………………… Page 76
69 Expanded ^{13}C NMR spectrum of **1e**…………………………………….. Page 77
70 MS spectrum of **1e**…………………………………………………………... Page 78
71 FT-IR spectrum of **2e**………………………………………………………... Page 79
72 ^{1}H NMR spectrum of **2e**…………………………………………………... Page 80
73 Expanded ^{1}H NMR spectrum of **2e**……………………………………… Page 81
74 ^{13}C NMR spectrum of **2e**………………………………………………… Page 82
75 Expanded ^{13}C NMR spectrum of **2e**…………………………………….. Page 83
76 MS spectrum of **2e**…………………………………………………………... Page 84

Table S1. Comparison of reaction conditions for the synthesis of sulfonamide compounds**.**

Entry	Method	Reactants	Conditions	Yield [%]	Ref.
1	chemical	sulfonic acids + Ph_3-O-PPh_3^{++} $2OTf^-$ +NUH + Et_3N	CH_2Cl_2, normal conditions	88-96	2
2	chemical	Aryl Chlorosulfates + Ar-$B(OH)_2$ + L-Pd(0) + R_1R_2NH	acetone, 50-70 ºC, 12 h	65-95	3
3	chemical	sodium sulfonates + amines + $CuBr_2$ + pyridine	DMSO, 24 h, O_2 (balloon), 100 ºC	61-97	4
4	chemical	NH-1,2,3-triazoles + aryl sulfonates + I_2	EtOAc, rt, 8-24 h	51-94	5
5	chemical	Azoles + sodium sulfonates + *N*-bromosuccinimide	dioxane, 25 ºC, 12 h	52-96	6
6	chemical	sulfonyl hydrazides + *tert*-amines + I_2 +*t*-butyl hydro-peroxide (TBHP)	H_2O, 80 ºC, 8 h	65-85	7
7	chemical	sodium aryl sulfinates+ R_1R_2NH+ ethylene dibromide + air + NaI	PEG-400/H_2O	43-82	9
8	chemical	sodium sulfonates + R_1R_2NH + (*n*-$C_4H_9)_4NBr$ + *m*-chloroperbenzoic acid	THF/MeOH, r.t. 12 h	up to 89	10
9	electrochemical	urazoles + sodium aryl sulfinates	phosphate buffer pH 3, 0.60 V vs. Ag/AgCl	79-91	12
10	electrochemical	sodium sulfonates + amines	I = 20 mA, 1 h, $LiClO_4$	48-98	14
11	electrochemical	sodium sulfonates + R_1R_2NH + NH_4I	graphite anode, Ni cathode, constant current, undivided cell	up to 76	15
12	electrochemical	thiols + amines	$MeNBF_4$ + CH_3CN, r.t., 5 min to 24 h, constant current, flow cell	up to 97	16
13	electrochemical	2-mercaptobenzoxazole + R_1R_2NH	phosphate buffer pH = 8/ethanol, constant current, undivided cell	59-79	17
14	chemical	boronic acids + DABSO + TBAB + *o*-benzoyl hydroxylamines + $Pd(OAc)_2$ + $CuBr_2$ + Na_2CO_3	dioxane/MeOH, 80 ºC, 5 + 12 h	up to 97	18
15	chemical	$Pd(OAc)_2$ + (*t*-$Bu)_3P.HBF_4$ + $K_2S_2O_5$ + TBAB	DMSO, 100 ºC, 18 h	up to 66	19
16	chemical	aryl boronic acids + R_1R_2NH + DABSO + $Cu(OTf)_2$ + Cs_2CO_3 + bipyridine	DMSO, 130 °C, 16 h	up to 77	20
17	electrochemical	halonitroarene + sodium aryl sulfinates	phosphate buffer pH = 2/CH_3CN, undivided cell, -0.8 V vs. Ag/AgCl, carbon anode	up to 86	this work

Differential pulse voltammogram of PINB

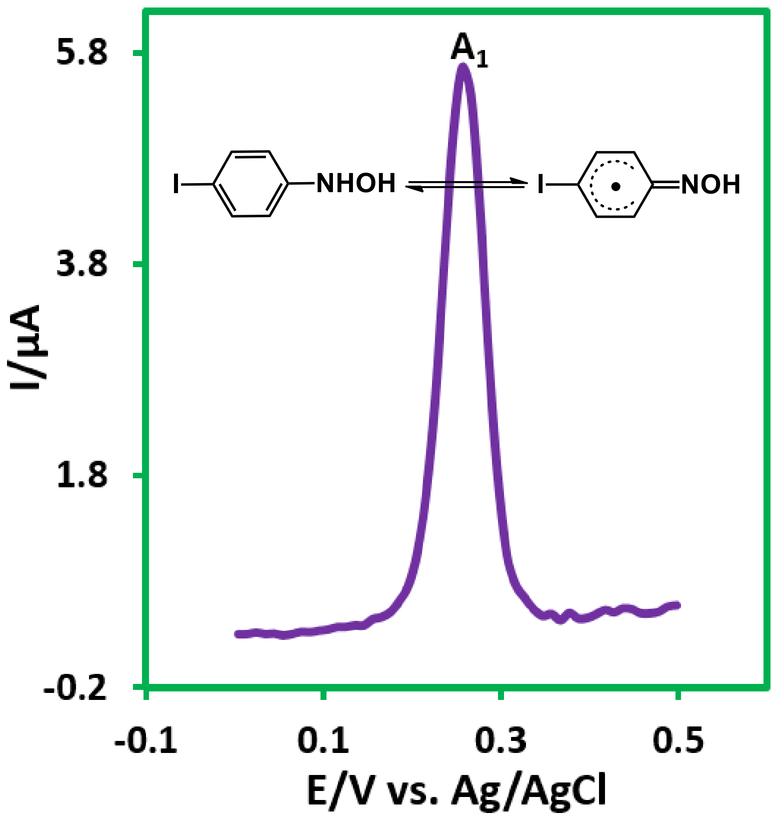

Figure S1 Differential pulse voltammogram of 1 mM **PINB** in aqueous phosphate buffer (pH, 2.0, c = 0.2 M)/acetonitrile (50/50 v/v) at glassy carbon electrode, at room temperature.

Optimized structure of NSB and PHI

NSB

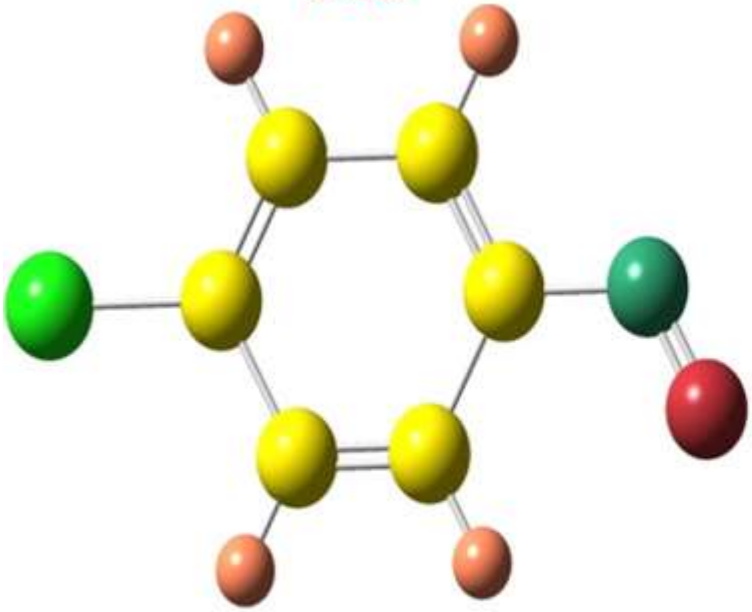

PHI

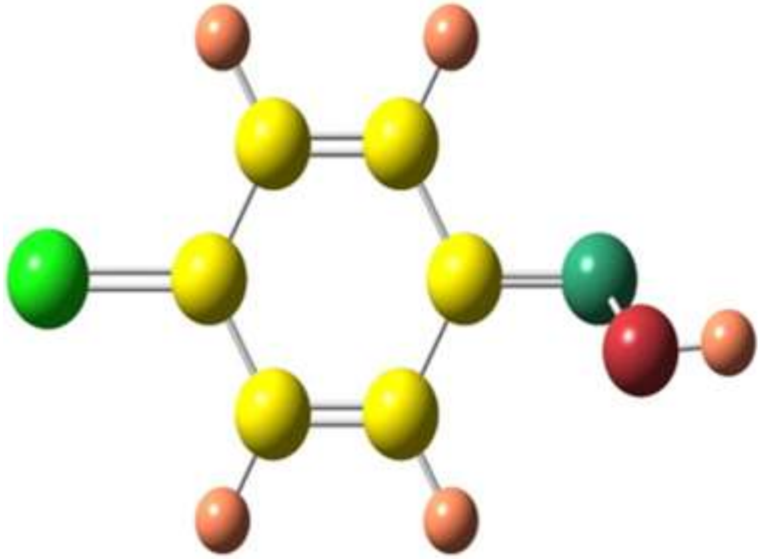

Figure S2 Optimized structure of **NSB** and **PHI** at B3LYP/6-311G level of theory.

Cyclic voltammograms of OINB

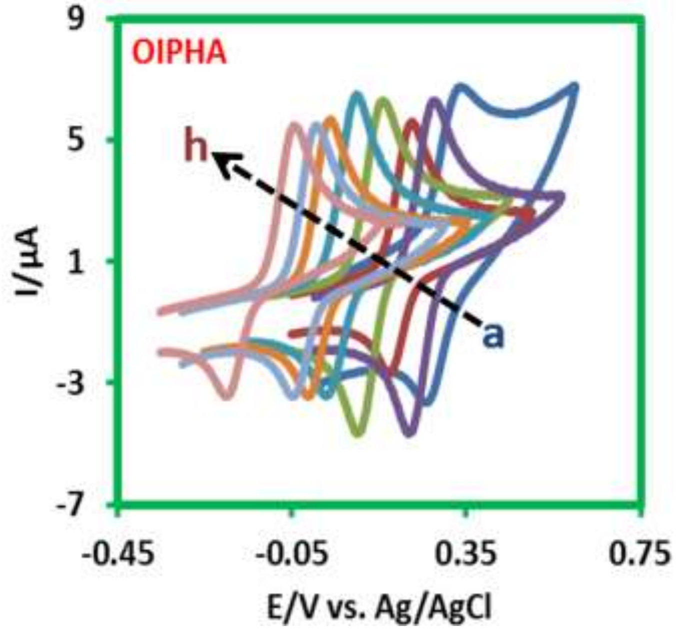

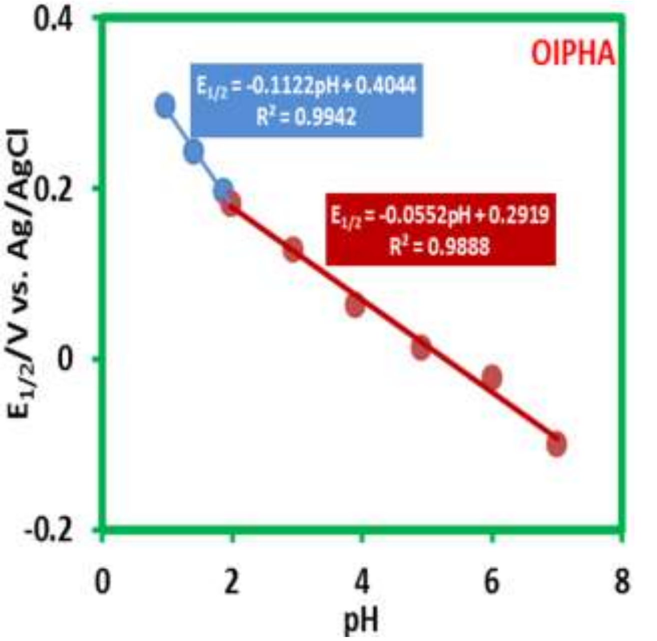

Figure S3. Cyclic voltammograms of **OINB** (1.0 mM) in buffer solution with various pH values/acetonitrile (50/50, v/v) mixture. pHs from a to h are: 0.97, 1.40, 1.87, 2.93, 3.90, 5.00, 6.00 and 7.00. Scan rate: 10 mV/s, at glassy carbon electrode, at room temperature. Below: Pourbaix diagram for

Cyclic voltammograms of PCNB

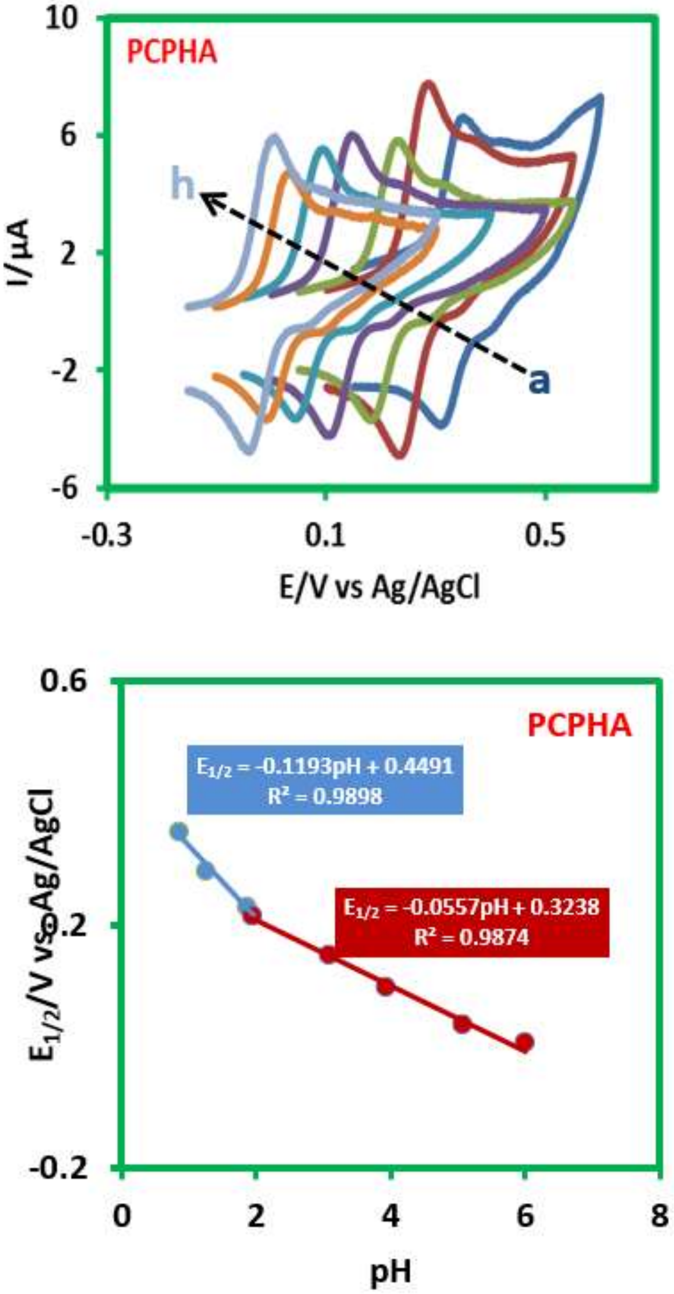

Figure S4. Cyclic voltammograms of **PCNB** (1.0 mM) in buffer solution with various pH values/ acetonitrile (50/50, v/v) mixture. pHs from a to h are: 0.97, 1.40, 1.87, 2.93, 3.90, 5.00, 6.00 and 7.00. Scan rate: 10 mV/s, at glassy carbon electrode, at room temperature. Below: Pourbaix diagram for **PCNB**.

Cyclic voltammograms of PBNB

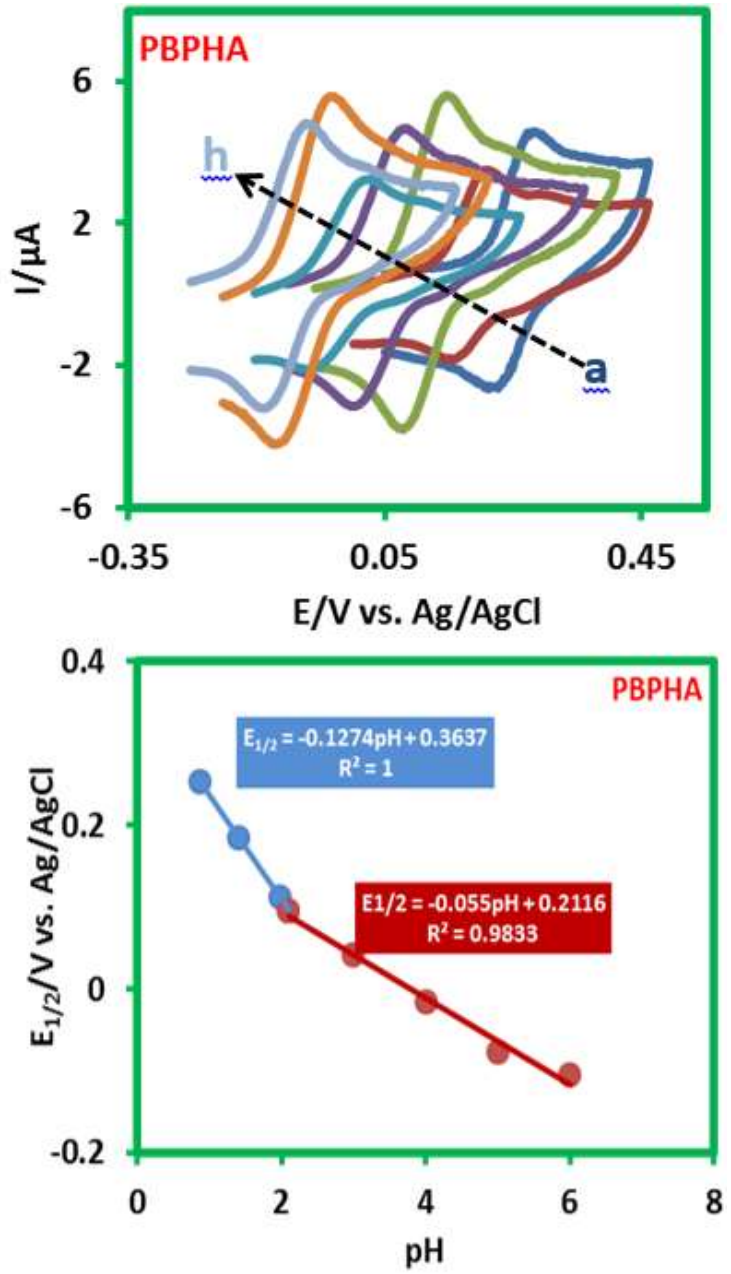

Figure S5. Cyclic voltammograms of **PBNB** (1.0 mM) in buffer solution with various pH values/ acetonitrile (50/50, v/v) mixture. pHs from a to h are: 0.97, 1.40, 1.87, 2.93, 3.90, 5.00, 6.00 and 7.00. Scan rate: 10 mV/s, at glassy carbon electrode, at room temperature. Below: Pourbaix diagram for **PBNB**.

Oxidation potential and E_{HOMO}

The oxidation potentials of aryl halide derivatives are determined by cyclic voltammetry and computational calculation. Table S2 shows that **PCPHA**, chloro substituent in *para* position was oxidized at higher positive potentials because of its higher electronegativity. But bromo substituent (**PBPHA**) was oxidized in the lower positive potential than iodo substituent (**PIPHA**) unexpectedly. For the study of this effect, HOMO orbital energies of these compounds were calculated (Figure S6). Higher HOMO orbital energy of **PIPHA** than **PBPHA**, confirms that oxidation of **PIPHA** is harder than **PBPHA**. Also, higher HOMO orbital energy of iodo compound in *ortho* position (**OIPHA**) in comparison with **PIPHA** proves the higher oxidation potential of **OIPHA**.

Table S2: Oxidation potential and E_{HOMO} of synthesized *N*-(4 or 2-halophenyl)hydroxylamine.

	E_{OX}/V	$-E_{HOMO}$
**PCPHA**	0.344	0.855
**PBPHA**	0.285	0.690
**PIPHA**	0.308	0.708
**OIPHA**	0.340	0.739

HOMO orbitals structure of *N*-(4 or 2-halophenyl)hydroxylamine

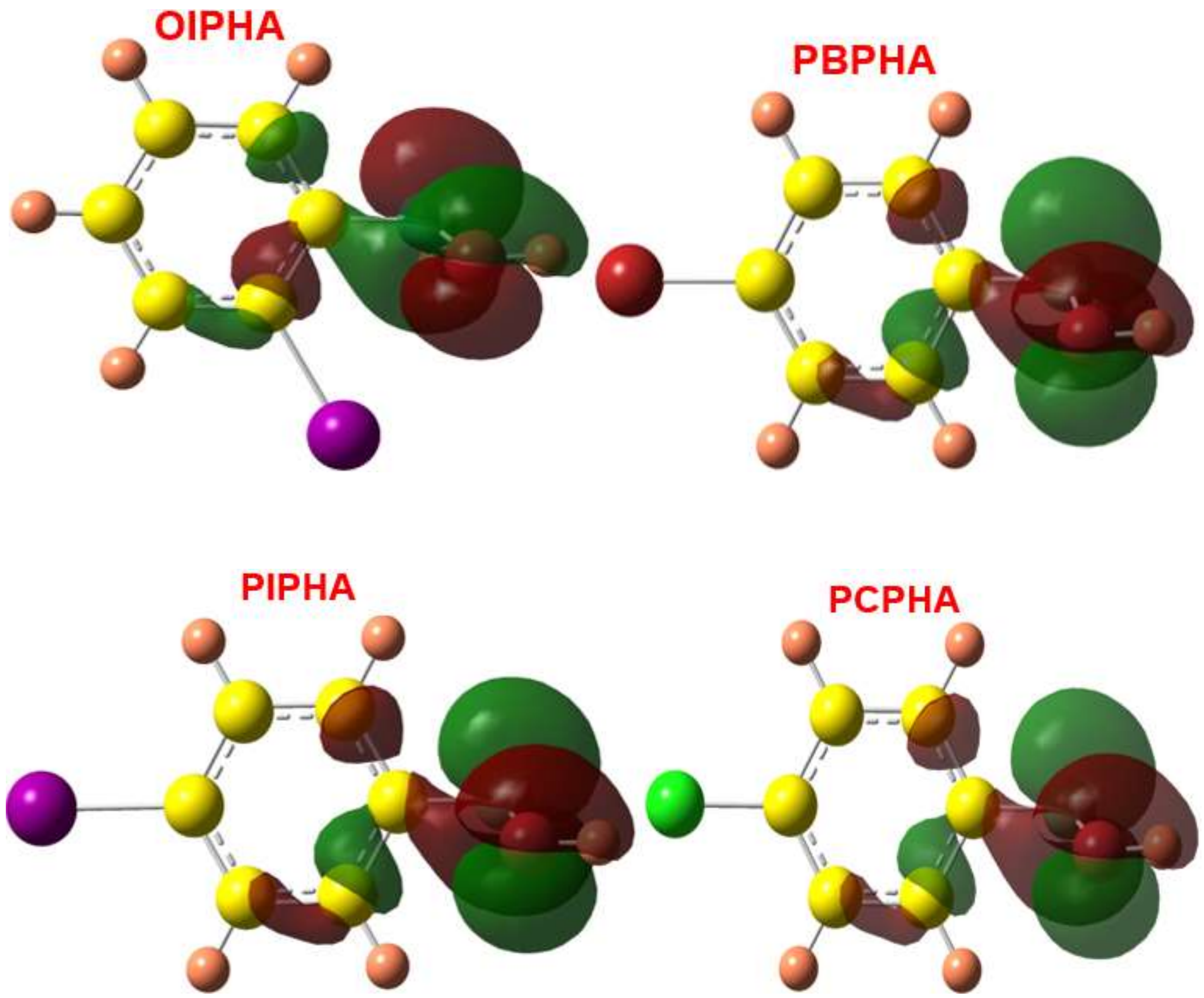

Figure S6: HOMO orbitals structure of **PCPHA, PBPHA, OIPHA** and **PIPHA.**

General procedure for synthesis of 1b-3b, 1c-3c, 1d-3d and 1e-2e

In an undivided cell equipped with four carbon rods as cathode and one carbon rod as anode, a solution (ca. 80 mL) of water (phosphate buffer, pH, 2.0, c = 0.2 M)/acetonitrile or DMF (50/50 v/v) containing **PINB** (0.5 mM) and **BSA** (0.5 mM) was electrolyzed at -0.8 V *vs*. Ag/AgCl. The electrolysis was terminated when the current decayed to 5% of its original value. At the end of the electrolysis, the cell was placed overnight. The precipitated pale yellow was collected by filtration and purified by plate chromatography (n-hexane/ethyl acetate 4/1).

Characterization of products

***N*-hydroxy-*N*-(4-iodophenyl) benzenesulfonamide (1b)**: Pale yellow; MP: 108-110 °C; ^{1}H NMR: δ ppm (500 MHz, CD_3CO-d_3): 6.99 (d, J = 10 Hz , 2H, aromatic), 7.56 (m, 4H, aromatic), 7.66 (d, J = 10 Hz, 2H, aromatic), 7.73 (m, 1H, aromatic), 10.21 (s, 1H, OH); ^{13}C NMR: δ ppm (125 MHz, CD_3CO-d_3: 91.2, 124.5, 128.6 , 129.5, 132.8, 134.0, 137.3, 143.0; IR (KBr) (cm^{-1}): 3334 (medium, O-H), 1477 (medium C=C), 1340 and 1165 (strong, S=O), 1084, 999, 685, 593, 572; MS (*m/z*) (EI, 70 EV) (relative intensity): 218 (100), 77 (100), 359 (80), 141 (50), 374 (M, 25).

***N*-hydroxy-*N*-(4-iodophenyl)-4-methylbenzenesulfonamide (2b)**: Pale yellow; MP: 157-158 °C; ^{1}H NMR: δ ppm (500 MHz, CD_3CO-d_3): 2.42 (s, 3H, methyl), 6.99 (d, J=10, 2H, aromatic), 7.35 (d, J = 10 Hz, 2H, aromatic), 7.43 (d, J = 10 Hz, 2H, aromatic), 7.66 (d, J = 10 Hz, 2H, aromatic), 10.30 (s, 1H, OH); ^{13}C NMR: δ ppm (125 MHz, CD_3CO-d_3: 20.7, 91.0, 124.5, 128.6 , 129.1, 129.5, 130.0, 137.2, 138.1, 143.1, 145.0; IR (KBr) (cm^{-1}): 3351 (medium, O-H), 1596 (medium C=C), 1475, 1337 and 1161 (strong, S=O), 1088, 990, 713, 664, 586, 548; MS (*m/z*) (EI, 70 EV) (relative intensity): 91 (100), 155 (85), 262 (25), 139 (35), 388 (M-1, 40).

4-Chloro-*N*-hydroxy-*N*-(4-iodophenyl)benzenesulfonamide (3b): Pale yellow; MP: 154-156 °C; ^{1}H NMR: δ ppm (500 MHz, CD_3CO-d_3): 7.01 (d, J=10, 2H, aromatic), 7.55 (d, J = 10 Hz, 2H, aromatic), 7.61 (d, J = 10 Hz, 2H, aromatic), 7.68 (d, J = 10 Hz, 2H, aromatic), 10.38 (s, 1H, OH); ^{13}C NMR: δ ppm (125 MHz, CD_3CO-d_3: 91.5, 124.6, 124.9 , 128.9, 131.2, 137.4, 138.9, 140.0, 142.6; IR (KBr) (cm^{-1}): 3357

(medium, O-H), 1571 (medium C=C), 1476, 1344 and 1165 (strong, S=O), 1091, 992, 829, 759, 620, 557; MS (*m/z*) (EI, 70 EV) (relative intensity): 111 (100), 175 (90), 282 (25), 393 (15), 408 (M-1, 40).

***N*-(4-chlorophenyl)-*N*-hydroxybenzenesulfonamide (1c)**: Pale yellow; MP: 102-104 ℃; ^{1}H NMR: δ ppm (500 MHz, CD_3CO-d_3): 7.18 (d, J = 10 Hz , 2H, aromatic), 7.33 (d, J=10, 2H, aromatic), 7.57 (m, 4H, aromatic), 7.73 (m, 1H, aromatic), 10.29 (s, 1H, OH); ^{13}C NMR: δ ppm (125 MHz, CD_3CO-d_3: 124.2, 128.2 , 128.5, 129.5, 132.0, 132.8, 134.0, 141.8; IR (KBr) (cm^{-1}): 3335 (medium, O-H), 1584 (medium C=C), 1481, 1348 and 1179 (strong, S=O), 1087, 1015, 831, 737, 608, 559, 577; MS (*m/z*) (EI, 70 EV) (relative intensity): 142 (100), 77 (70), 111 (45), 267 (10), 283 (M, 15).

***N*-(4-chlorophenyl)-*N*-hydroxy-4-methylbenzenesulfonamide (2c)**: Pale yellow; MP: 129-130 °C; ^{1}H NMR: δ ppm (500 MHz, CD_3CO-d_3): 2.42 (S, 3H, methyl), 7.19 (d, J=10, 2H, aromatic), 7.32 (d, J = 10 Hz, 2H, aromatic), 7.35 (d, J = 10 Hz, 2H, aromatic), 7.43 (d, J = 10 Hz, 2H, aromatic), 10.23 (s, 1H, OH); ^{13}C NMR: δ ppm (125 MHz, CD_3CO-d_3: 20.5, 124.1, 128.2 , 129.1, 129.5, 129.9, 131.8, 141.9, 145.0; IR (KBr) (cm^{-1}): 3346 (medium, O-H), 2926 (weak, CH_3) 1595 (medium C=C), 1482, 1342 and 1163 (strong, S=O), 1086, 889, 831, 814, 731, 671, 555; MS (*m/z*) (EI, 70 EV) (relative intensity): 91 (100), 142 (90), 111 (60), 65 (60), 281(30), 297 (M-1, 15).

4-Chloro-*N*-(4-chlorophenyl)-*N*-hydroxybenzenesulfonamide (3c): Pale yellow; MP: 142-146 ℃; ^{1}H NMR: δ ppm (500 MHz, CD_3CO-d_3): 7.20 (d, J=10, 2H,

aromatic), 7.35 (d, J = 10 Hz, 2H, aromatic), 7.55 (d, J = 10 Hz, 2H, aromatic), 7.61 (d, J = 10 Hz, 2H, aromatic), 10.39 (s, 1H, OH); ^{13}C NMR: δ ppm (125 MHz, CD_3CO-d_3: 124.2, 128.4, 128.9 , 131.2, 131.4, 132.2, 140.0, 141.5; IR (KBr) (cm^{-1}): 3349 (medium, O-H), 1574 (medium C=C), 1482, 1350 and 1182 (strong, S=O), 1090, 1013, 834, 760, 638, 589, 562; MS (*m/z*) (EI, 70 EV) (relative intensity): 111 (100), 175 (85), 142 (25), 301 (10), 316 (M-1, 20).

***N*-hydroxy-*N*-(2-iodophenyl)benzenesulfonamide (1d)**: Pale yellow; MP: 142-144 ℃; ^{1}H NMR: δ ppm (500 MHz, CD_3CO-d_3): 6.78 (d, J = 10 Hz , 1H, aromatic), 7.11 (t, 1H, aromatic), 7.30 (t, 1H, aromatic), 7.68 (m, 2H, aromatic), 7.81 (m, 3H, aromatic), 7.98 (d, J=10 Hz, 1H, aromatic), 10.19 (s, 1H, OH); ^{13}C NMR: δ ppm (125 MHz, CD_3CO-d_3: 99.0, 126.3, 128.5 , 128.7, 130.1, 130.3, 134.3, 139.6, 144.5; IR (KBr) (cm^{-1}): 3329 (medium, O-H), 1451 (medium C=C), 1350 and 1175 (strong, S=O), 1087, 908, 767, 692, 571; MS (*m/z*) (EI, 70 EV) (relative intensity): 224 (100), 203 (80), 218 (40), 359 (40), 375 (M, 25).

***N*-hydroxy-*N*-(2-iodophenyl)-4-methylbenzenesulfonamide (2d)**: Pale yellow; MP: 162-164 ℃; ^{1}H NMR: δ ppm (500 MHz, CD_3CO-d_3): 2.49 (S, 3H, methyl), 7.1 (t, 1H, aromatic), 7.29 (t, 1H, aromatic), 7.45 (d, J = 10 Hz, 2H, aromatic), 7.68 (d, J = 10 Hz, 2H, aromatic), 7.94 (d, J = 10 Hz, 2H, aromatic), 10.19 (s, 1H, OH); ^{13}C NMR: δ ppm (125 MHz, CD_3CO-d_3: 99.0, 126.2, 128.5 , 129.2, 130.2, 131.3, 139.5, 144.6, 145.1; IR (KBr) (cm^{-1}): 3337 (medium, O-H), 1463 (medium C=C), 1344 and 1165 (strong,

S=O), 1087, 769, 710, 665, 577; MS (*m/z*) (EI, 70 EV) (relative intensity): 223 (100), 203 (60), 91 (70), 373 (20), 389 (M, 25).

4-Chloro-*N*-hydroxy-*N*-(2-iodophenyl)benzenesulfonamide (3d): Pale yellow; MP: 154-156 °C; ^{1}H NMR: δ ppm (500 MHz, CD_3CO-d_3): 6.84 (d, J = 10 Hz , 1H, aromatic), 7.13 (t, 1H, aromatic), 7.34 (t, 1H, aromatic), 7.71 (d, J=10, 2H, aromatic), 7.81 (d, J=10, 2H, aromatic), 7.96 (d, J = 10 Hz , 1H, aromatic), 10.19 (s, 1H, OH); ^{13}C NMR: δ ppm (125 MHz, CD_3CO-d_3): 99.0, 126.1, 128.7 , 129.0, 130.4, 131.8, 132.8, 139.6, 140.1, 144.2; IR (KBr) (cm^{-1}): 3350 (medium, O-H), 1464 (medium C=C), 1348 and 1172 (strong, S=O), 1091, 764, 707, 640, 574; MS (*m/z*) (EI, 70 EV) (relative intensity): 223 (100), 203 (75), 111(60), 393 (20), 409 (M, 15).

***N*-(4-bromophenyl)-*N*-hydroxybenzenesulfonamide (1e)**: Pale yellow; MP: 92-94 °C; ^{1}H NMR: δ ppm (500 MHz, CD_3CO-d_3): 7.14 (d, J = 10 Hz , 2H, aromatic), 7.51 (d, J = 10 Hz, 2H, aromatic), 7.58 (m, 4H, aromatic),7.75 (m, 1H, aromatic), 10.31 (s, 1H, OH); ^{13}C NMR: δ ppm (125 MHz, CD_3CO-d_3): 119.9, 124.5, 128.6, 129.5, 131.3, 132.8, 134.0, 142.3; IR (KBr) (cm^{-1}): 3341 (medium, O-H), 1480 (medium C=C), 1342 and 1163 (strong, S=O), 1069, 886, 720, 667, 591; MS (*m/z*) (EI, 70 EV) (relative intensity): 77 (100), 141 (40), 51 (50), 311 (10), 326 (M, 25).

***N*-(4-bromophenyl)-*N*-hydroxy-4-methylbenzenesulfonamide (2e)**: Pale yellow; MP: 113-114 °C; ^{1}H NMR: δ ppm (500 MHz, CD_3CO-d_3): 2.44 (S, 3H, methyl), 7.15 (d, J=10, 2H, aromatic), 7.37 (d, J = 10 Hz, 2H, aromatic), 7.45 (d, J = 10 Hz, 2H,

aromatic), 7.49 (d, *J* = 10 Hz, 2H, aromatic), 10.24 (s, 1H, OH);^{13}C NMR: δ ppm (125 MHz, CD_3CO-d_3: 21.0, 119.7, 124.4, 129.2, 129.6, 129.9, 131.2, 142.8, 145.0; IR (KBr) (cm^{-1}): 3341 (medium, O-H), 1480 (medium C=C), 1342 and 1163 (strong, S=O), 1069, 987, 667, 591, 553; MS (*m/z*) (EI, 70 EV) (relative intensity): 91 (100), 155 (80), 187 (60), 327 (50), 343 (M, 25).

FT-IR spectrum of 1b

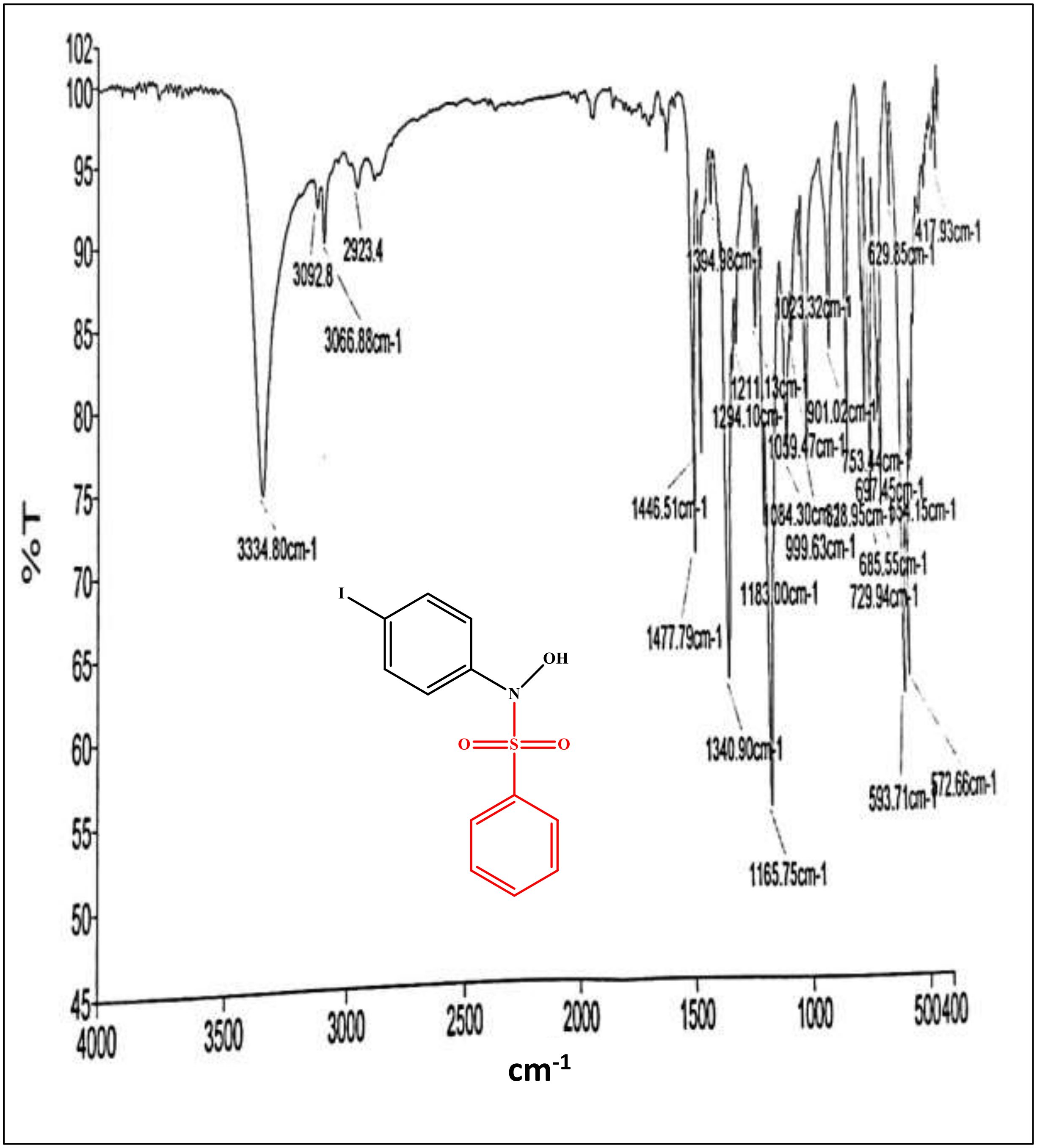

^{1}H NMR spectrum of 1b

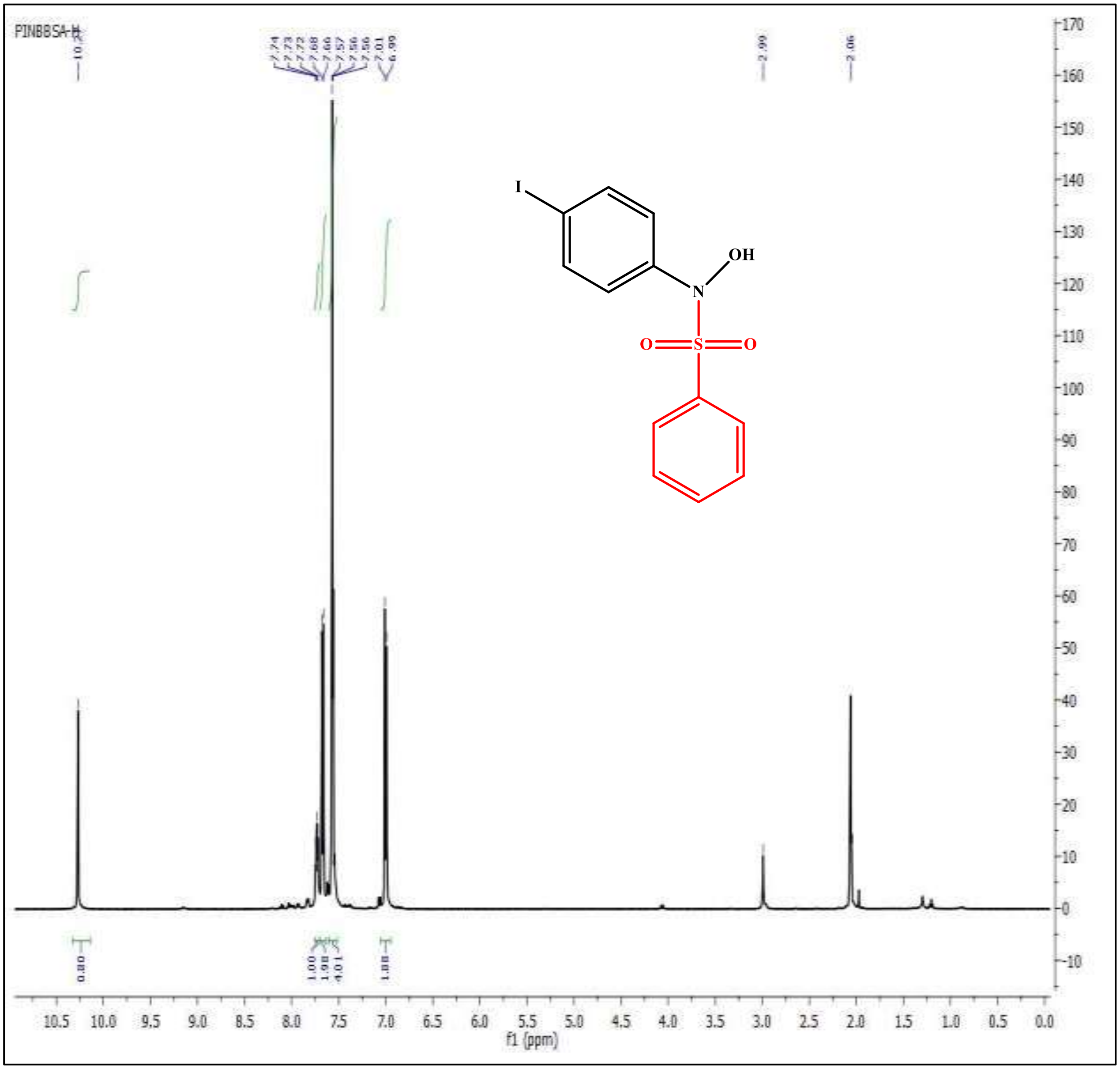

Expanded ¹H NMR spectrum of 1b

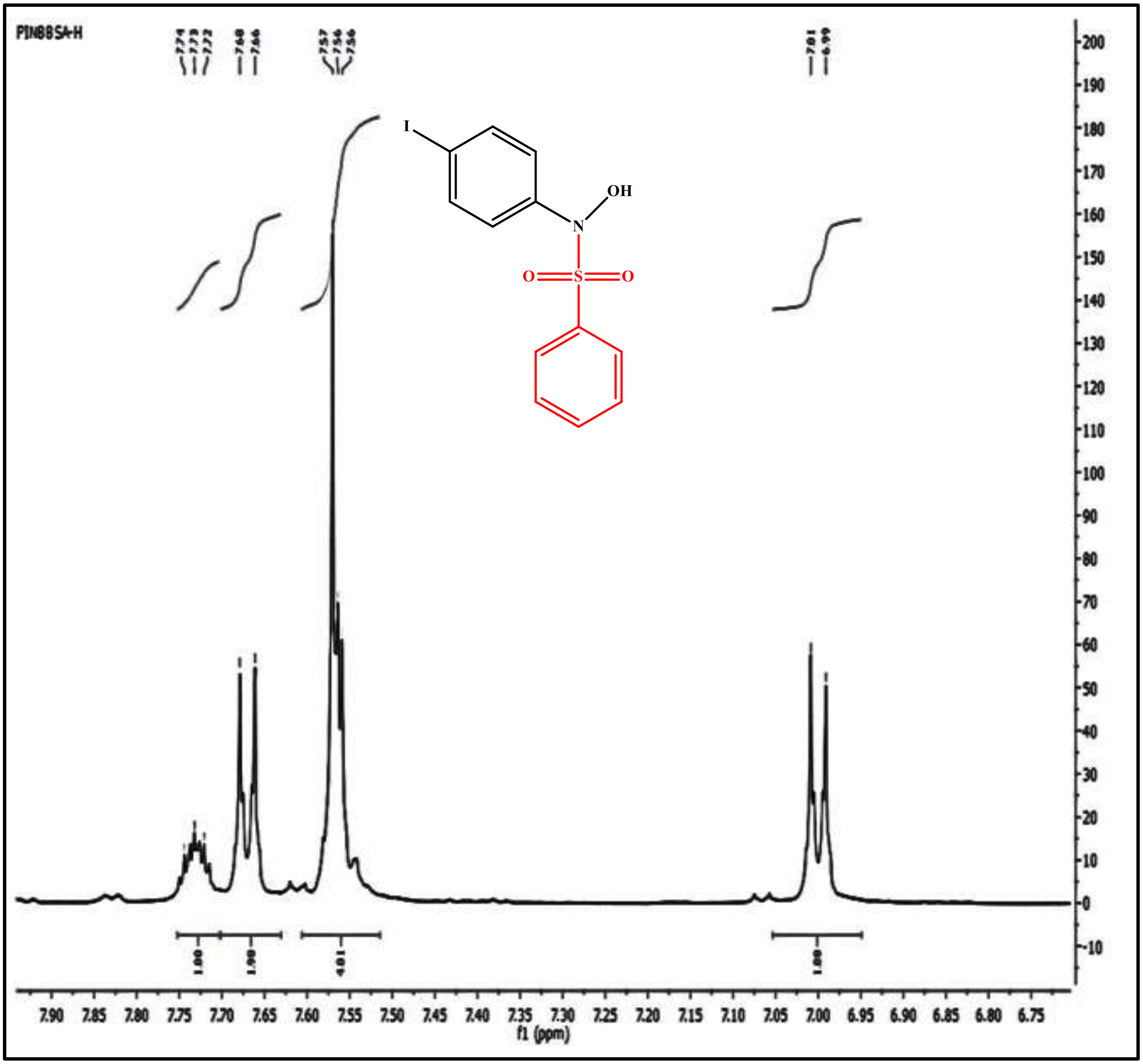

^{13}C NMR spectrum of 1b

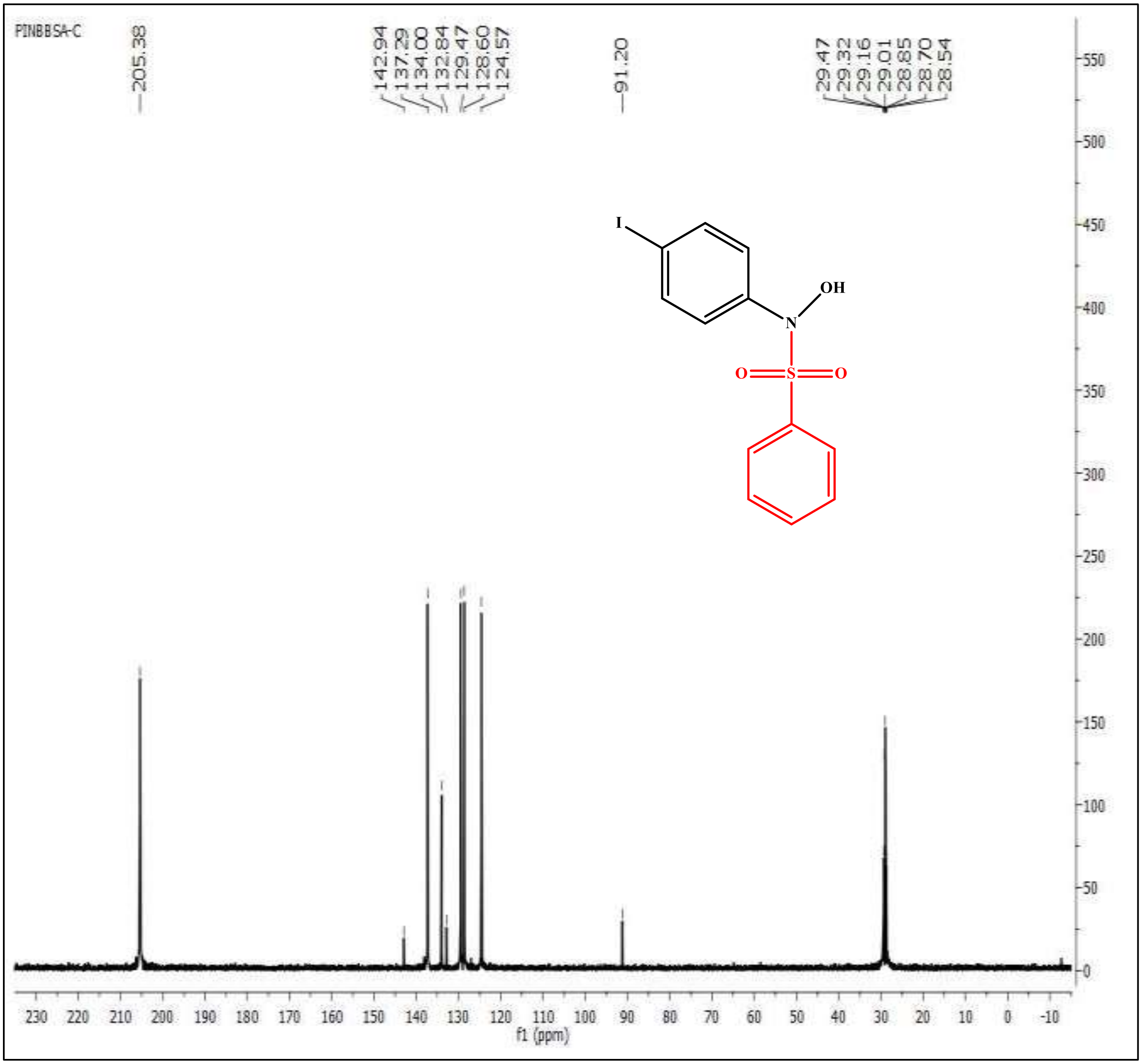

Expanded ^{13}C NMR spectrum of 1b

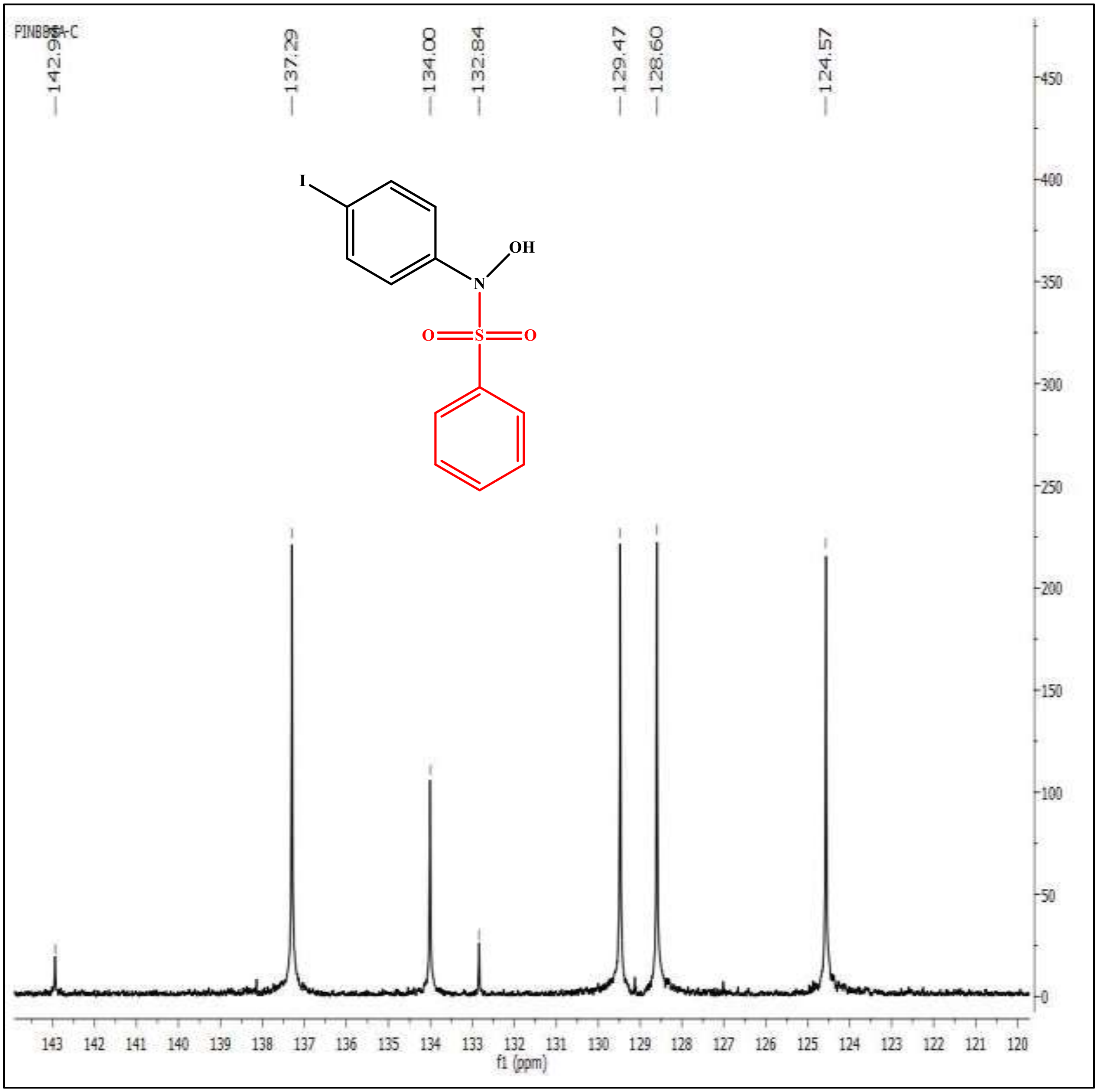

MS spectrum of 1b

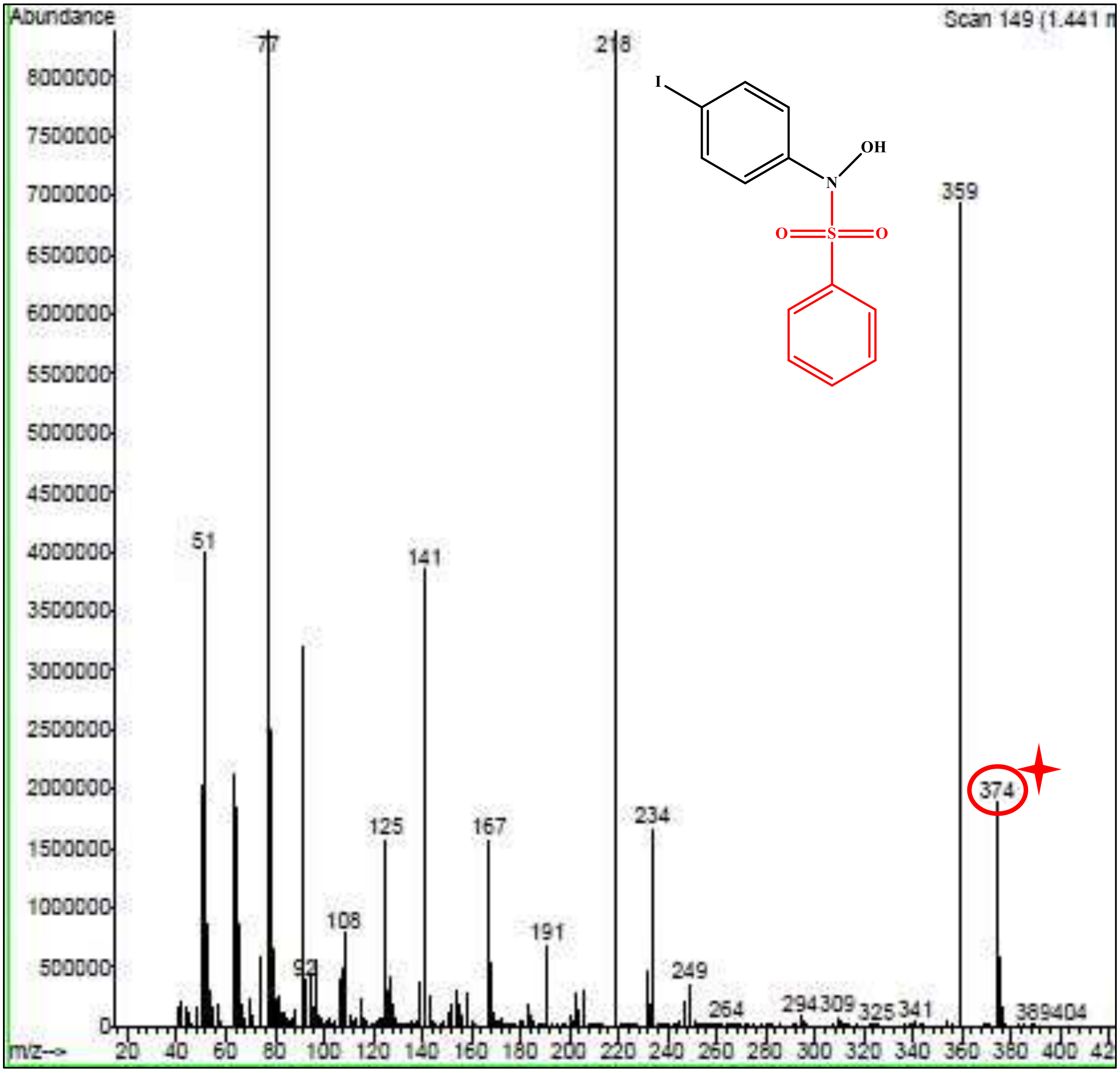

FT-IR spectrum of 2b

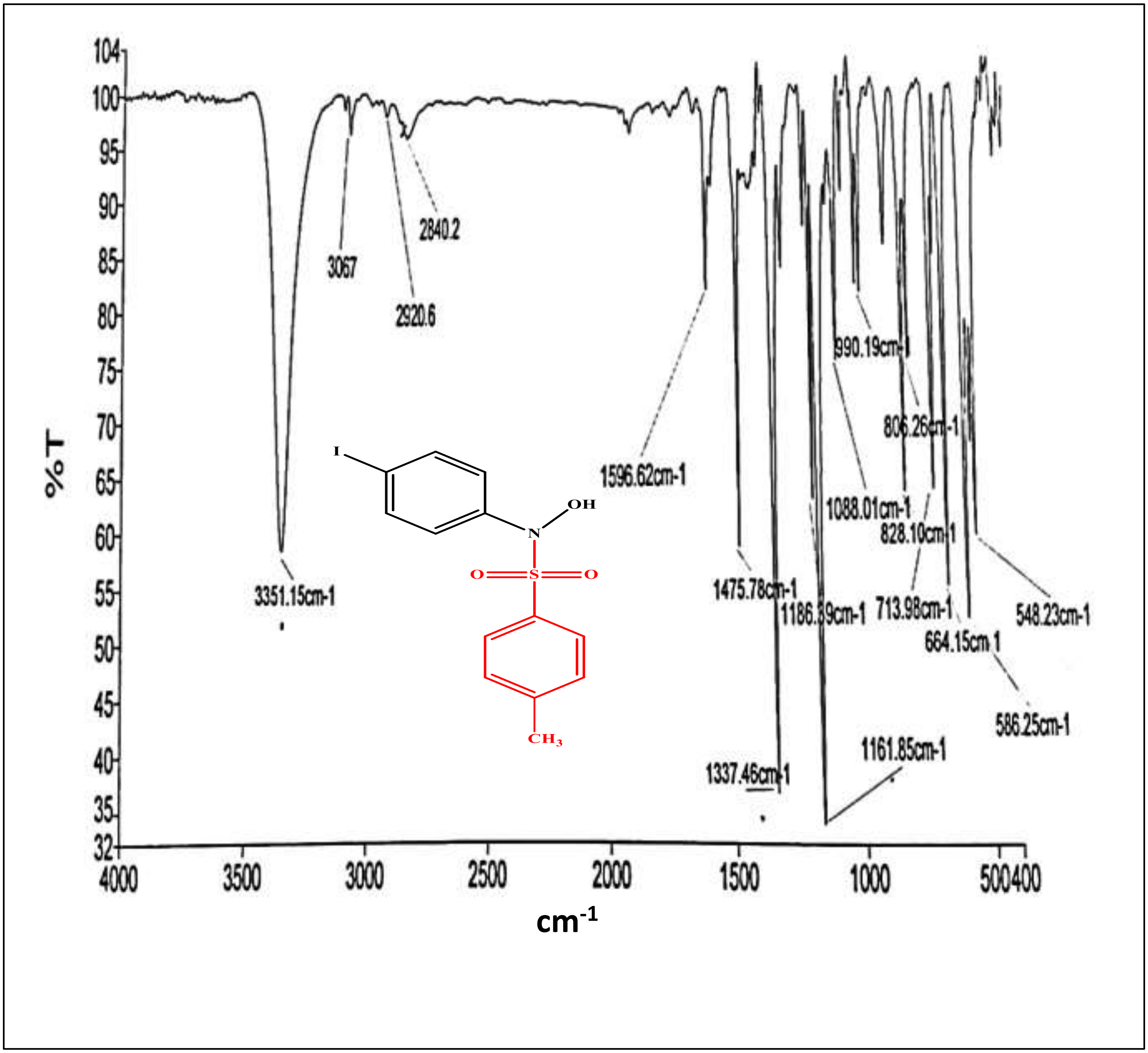

^{1}H NMR spectrum of 2b

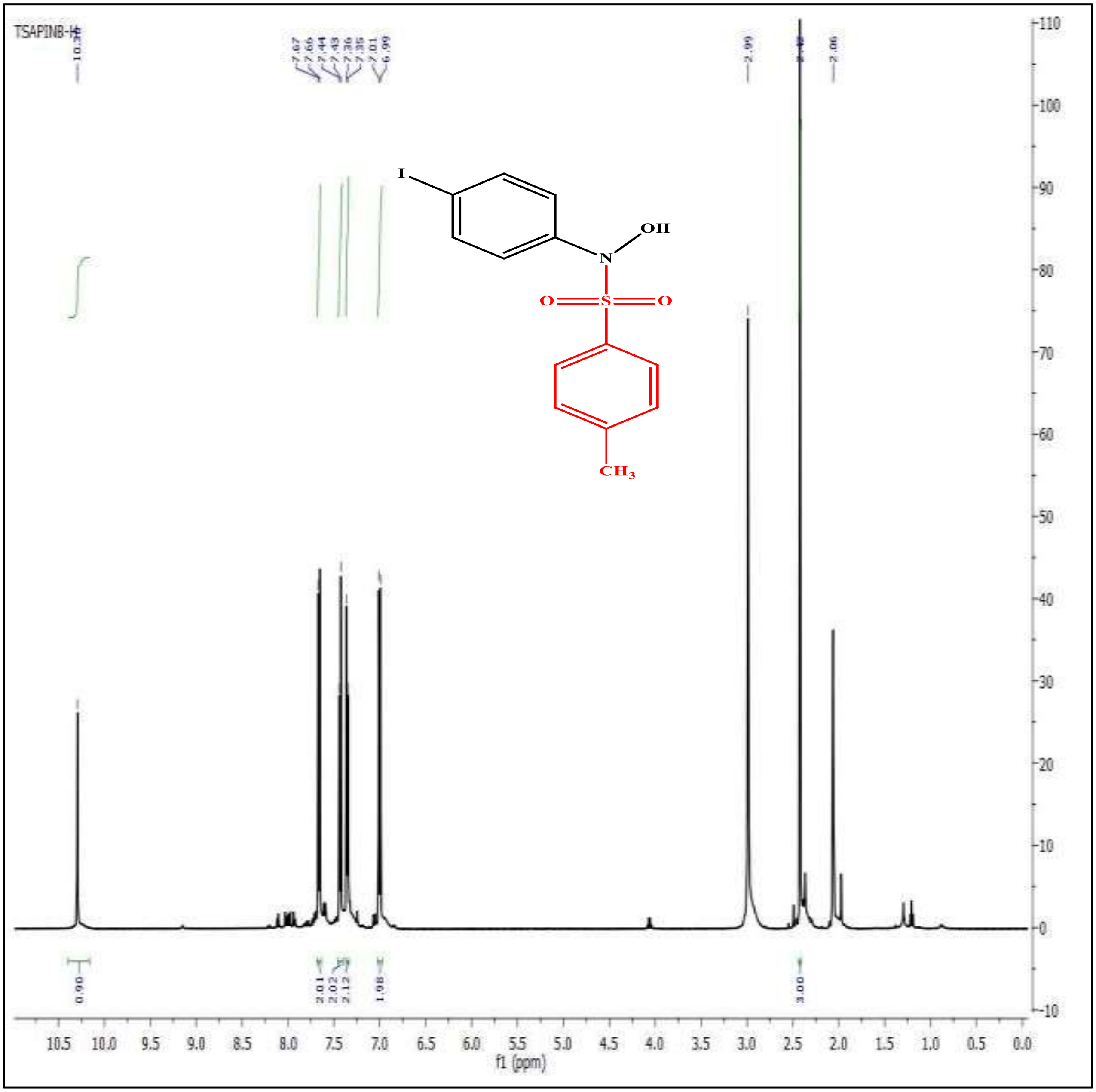

Expanded ^{1}H NMR spectrum of 2b

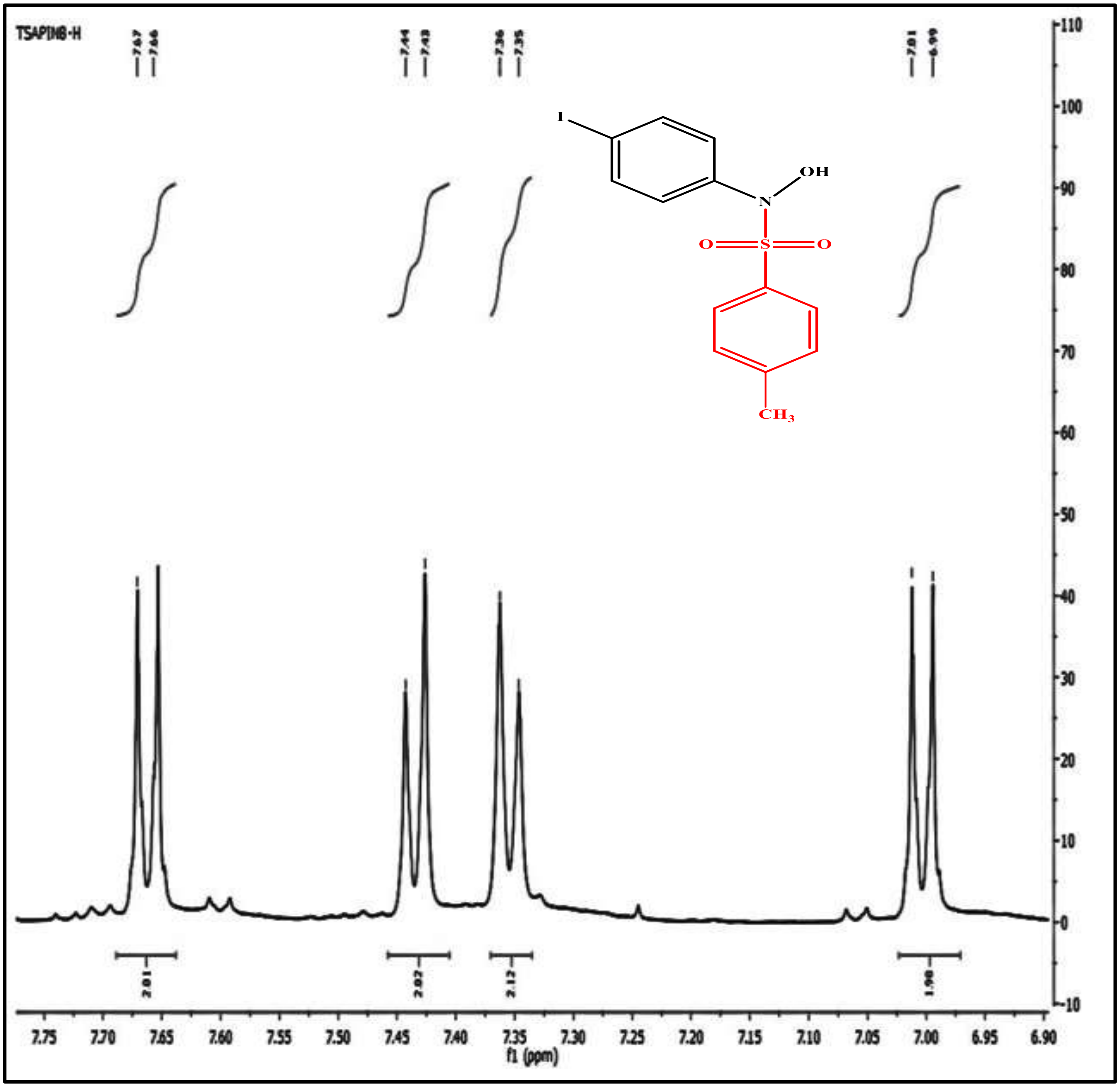

^{13}C NMR spectrum of 2b

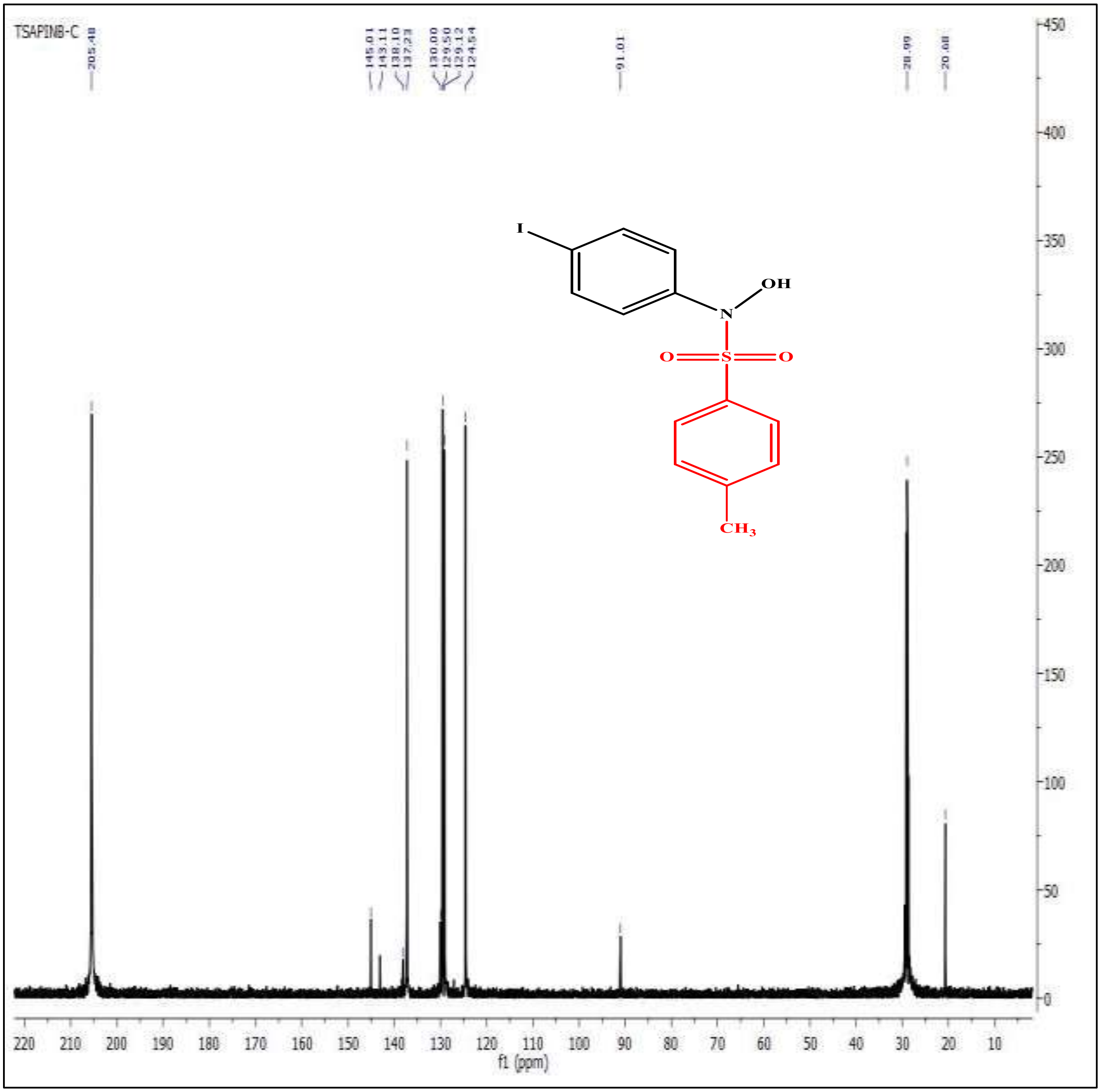

Expanded ^{13}C NMR spectrum of 2b

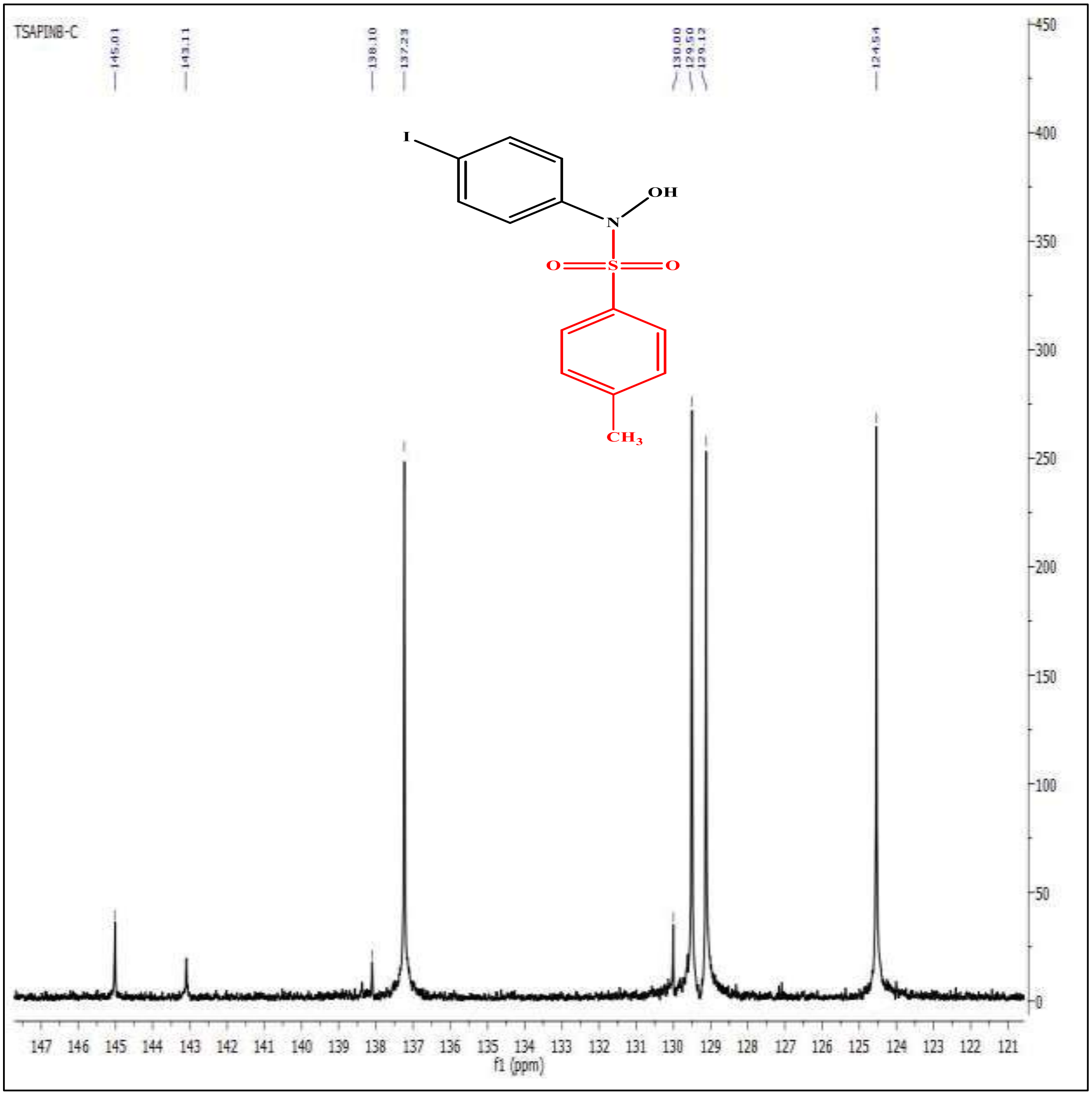

MS spectrum of 2b

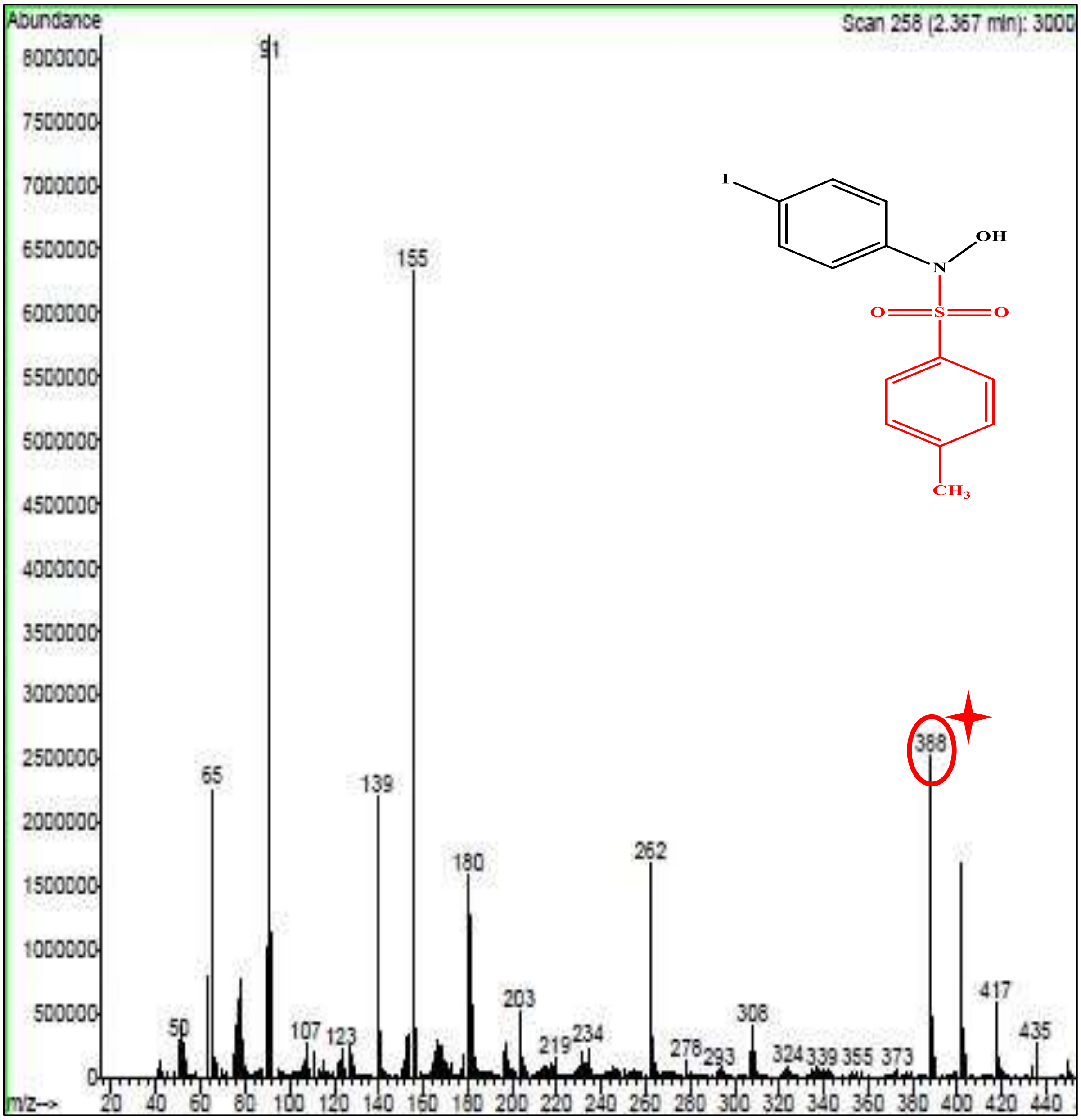

FT-IR spectrum of 3b

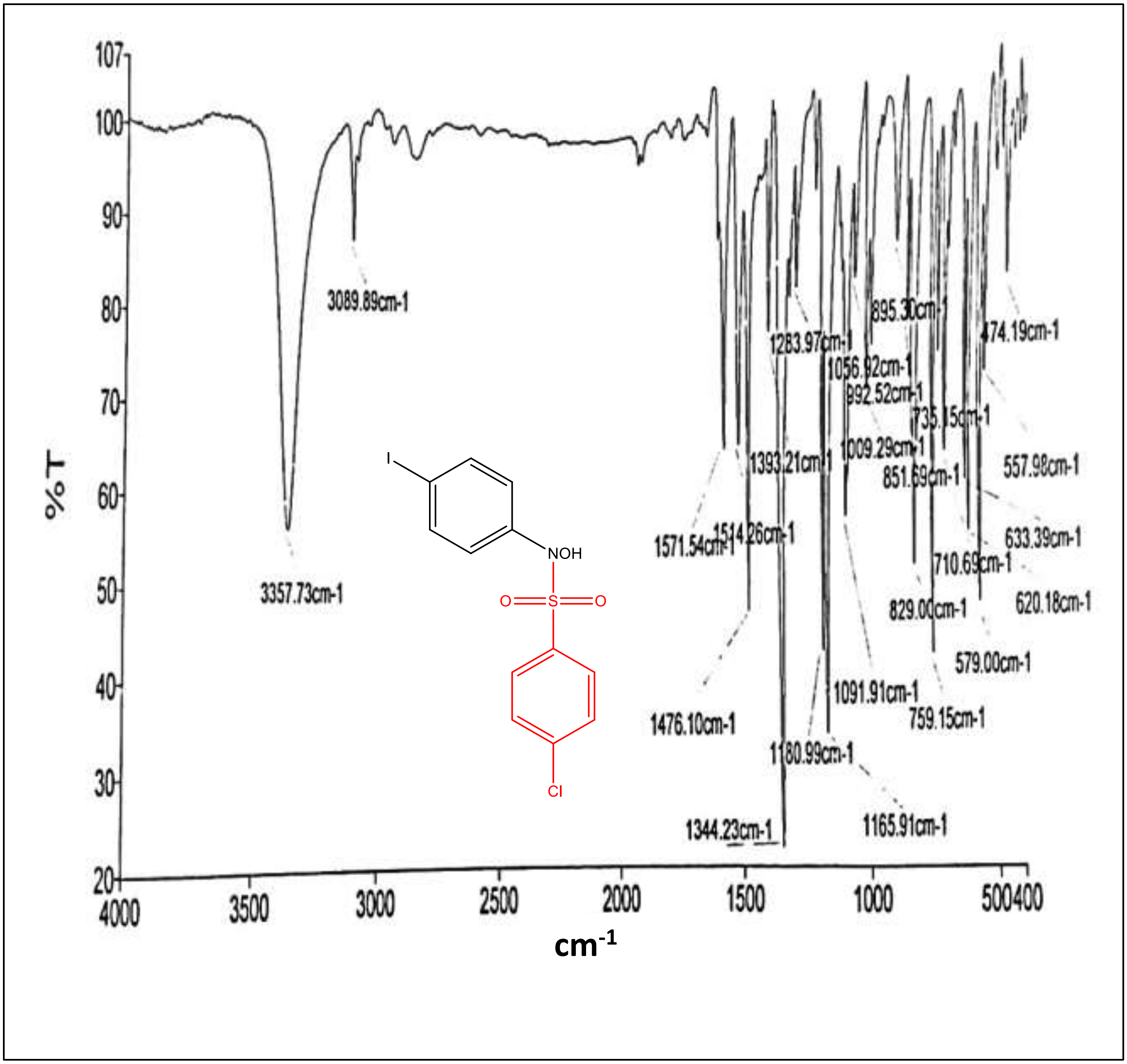

^{1}H NMR spectrum of 3b

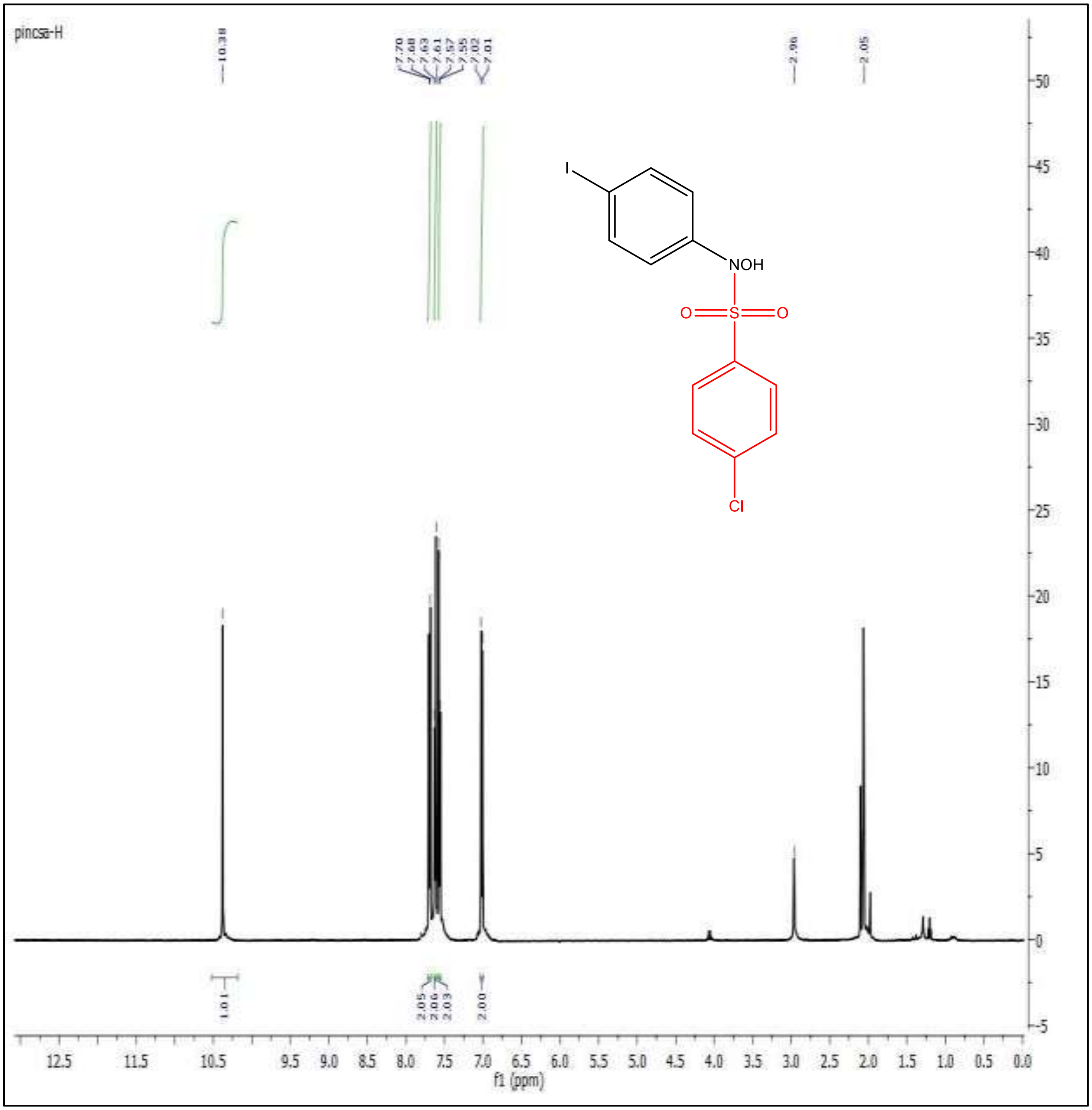

Expanded ^{1}H NMR spectrum of 3b

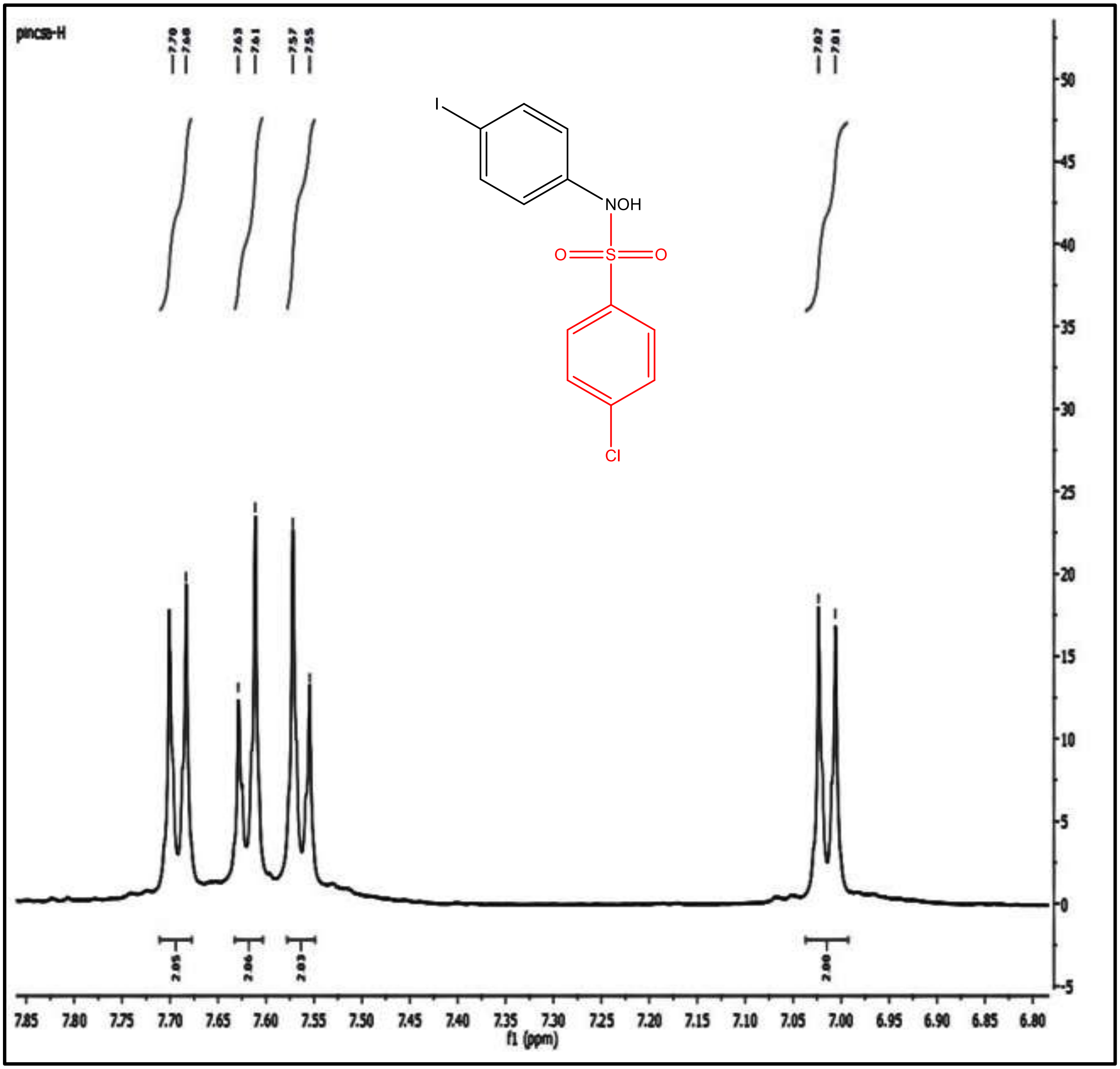

^{13}C NMR spectrum of 3b

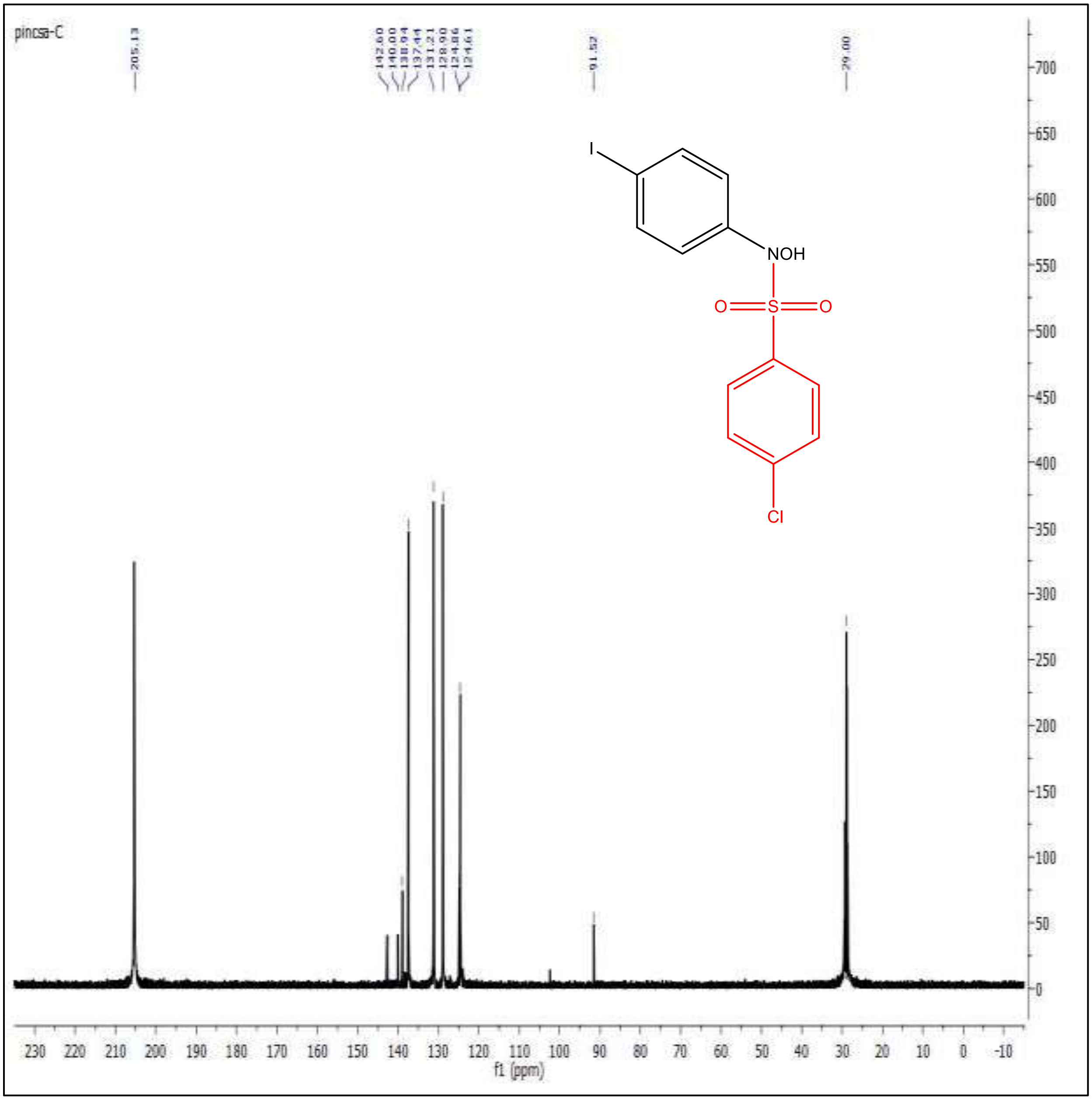

Expanded ^{13}C NMR spectrum of 3b

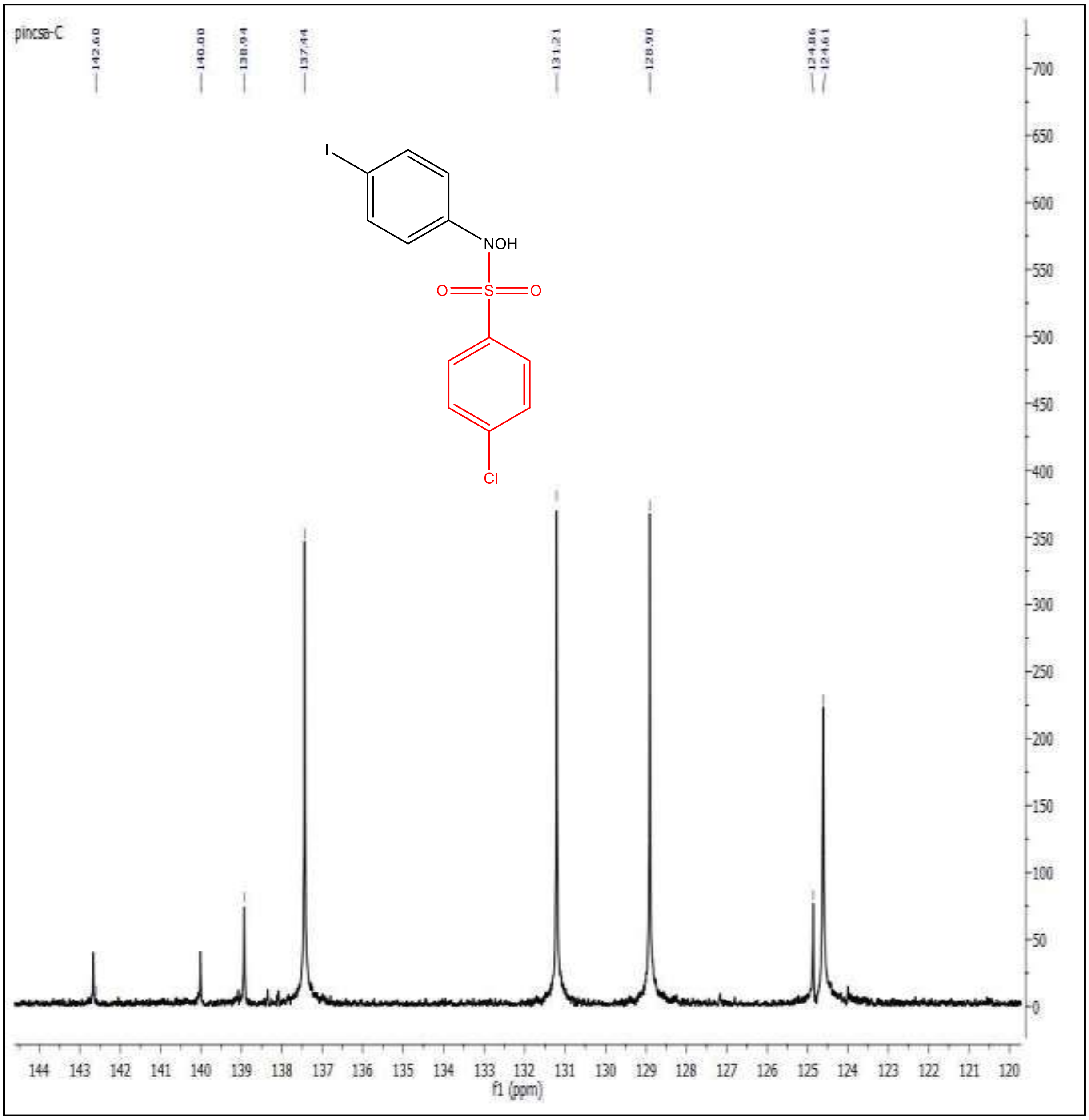

MS spectrum of 3b

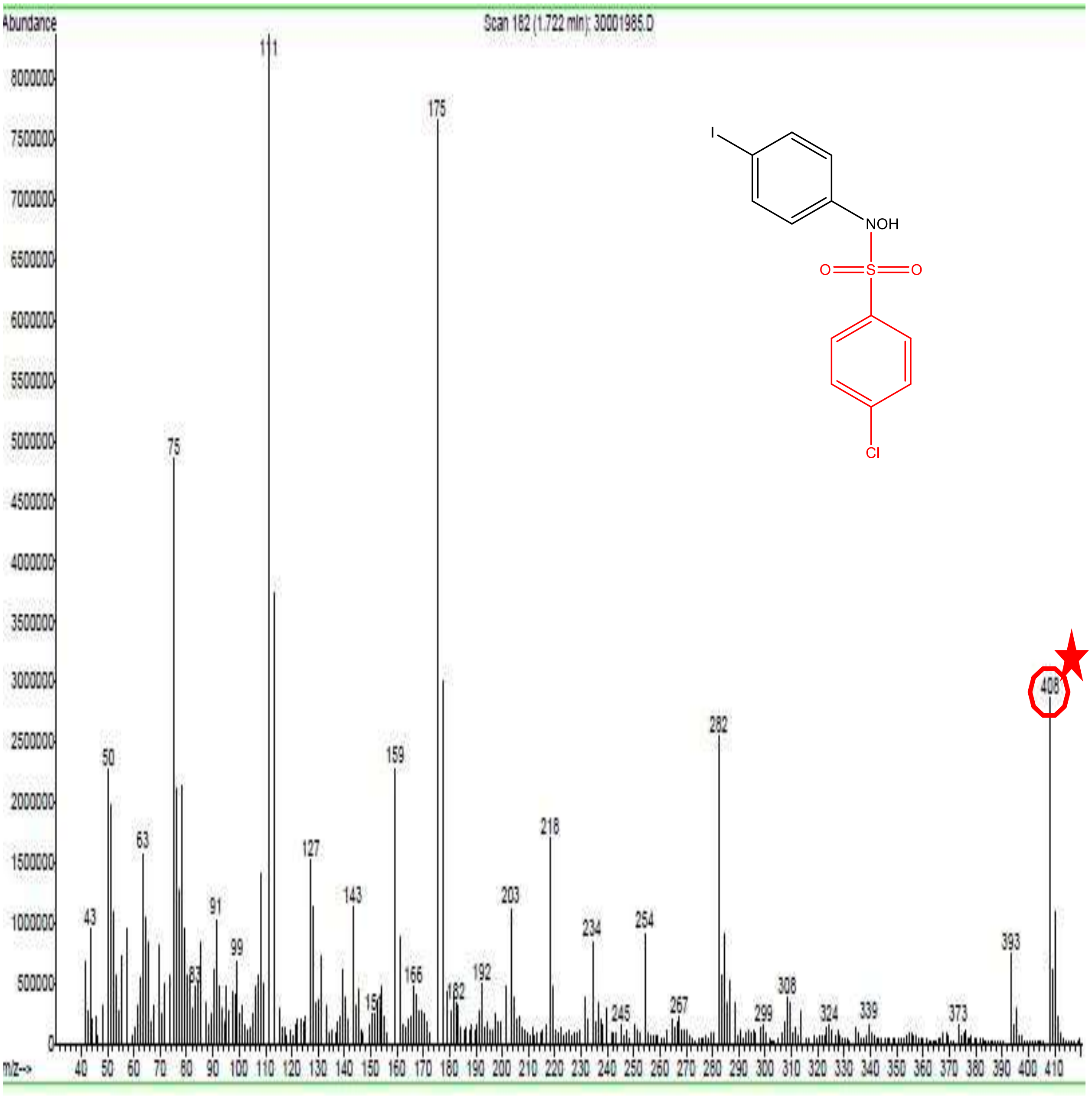

FT-IR spectrum of 1c

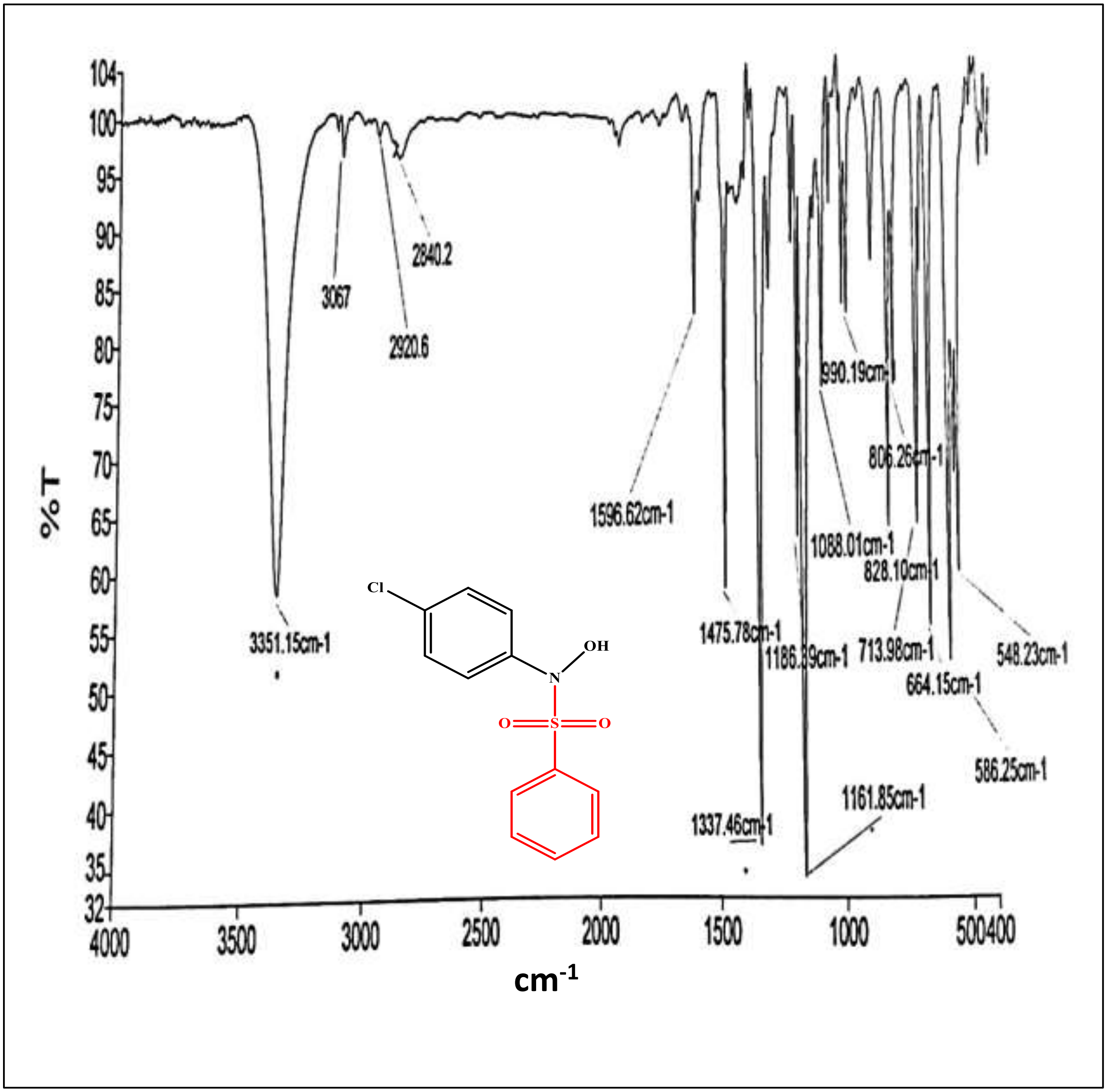

^{1}H NMR spectrum of 1c

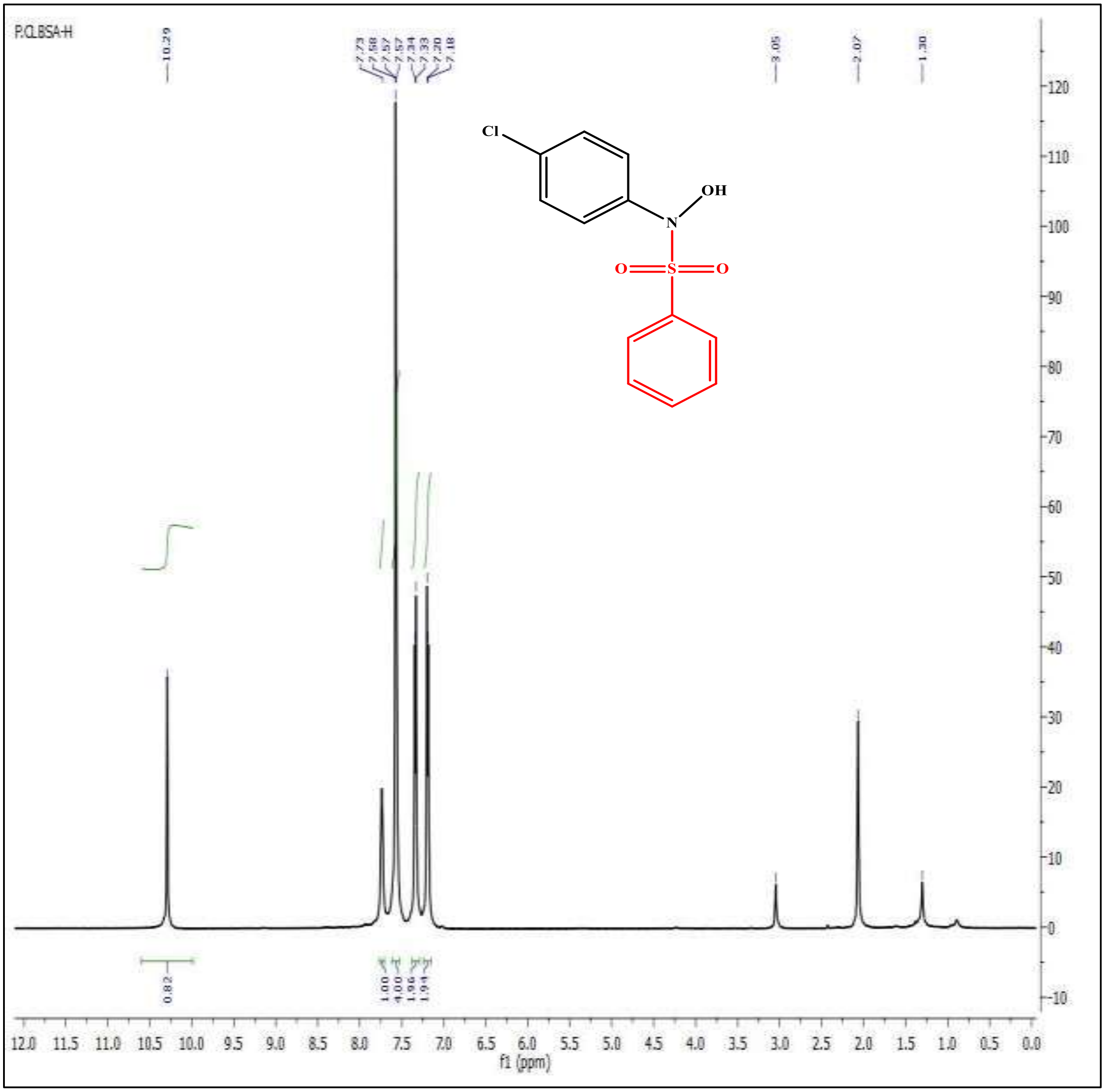

Expanded ^{1}H NMR spectrum of 1c

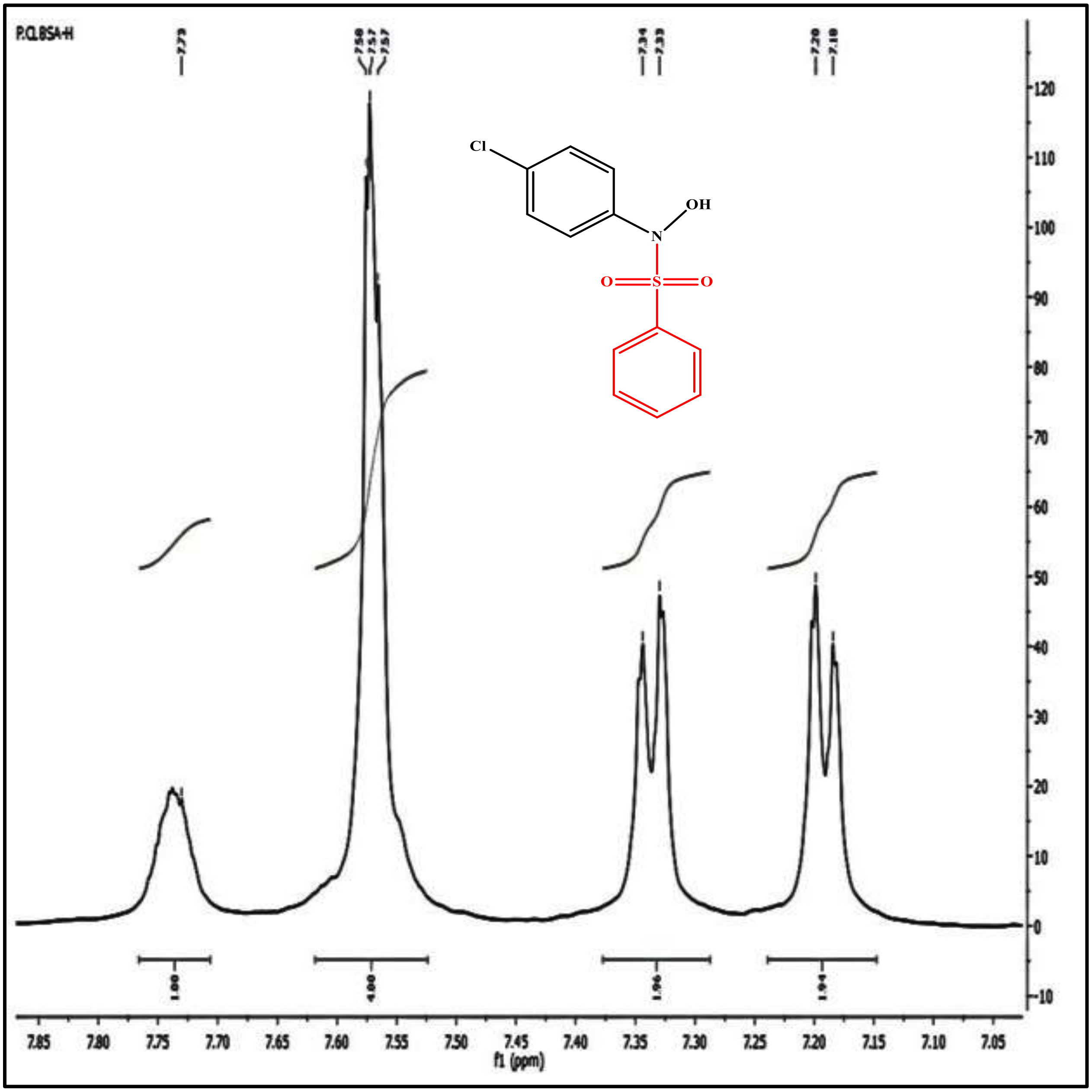

^{13}C NMR spectrum of 1c

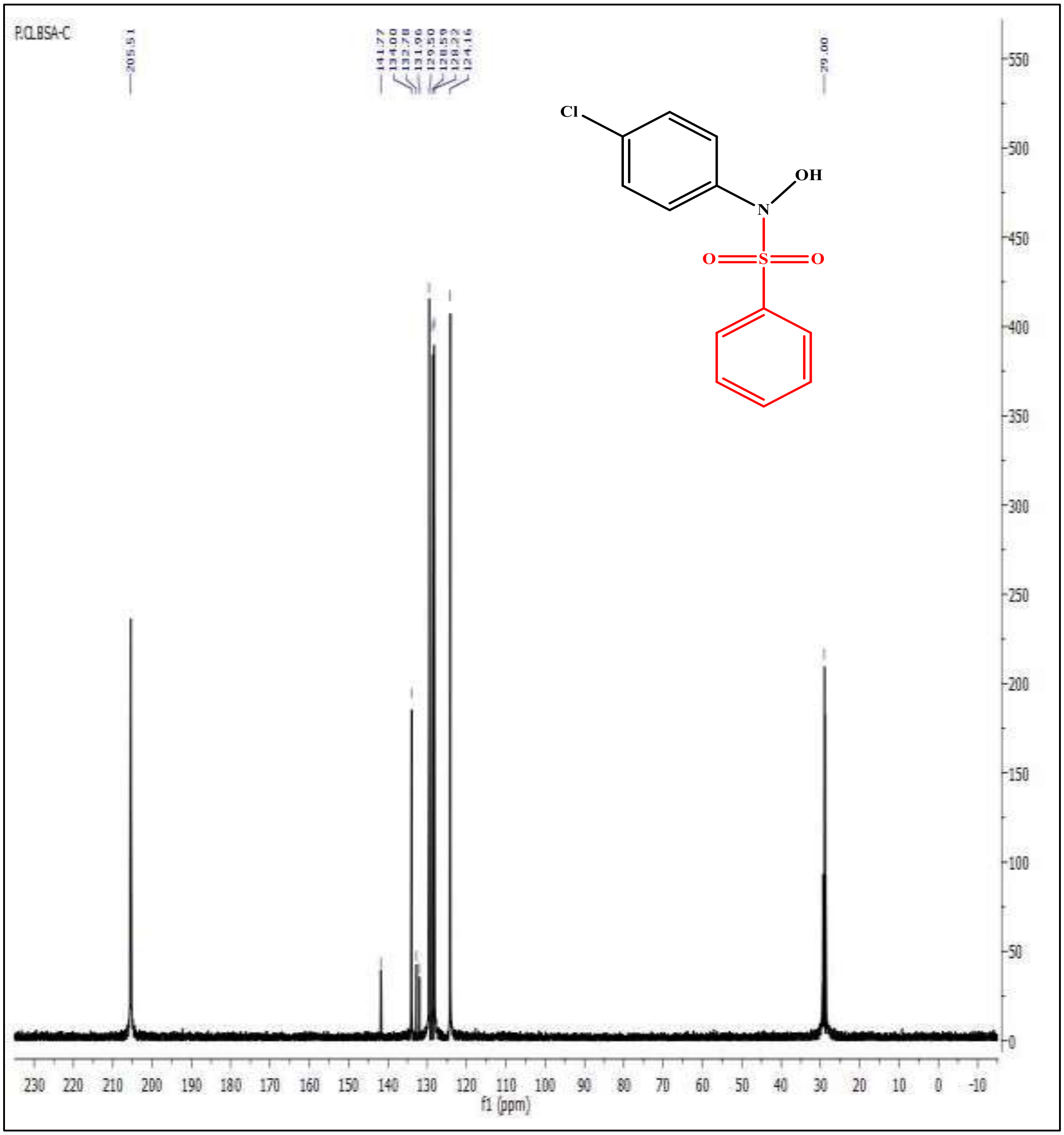

Expanded ^{13}C NMR spectrum of 1c

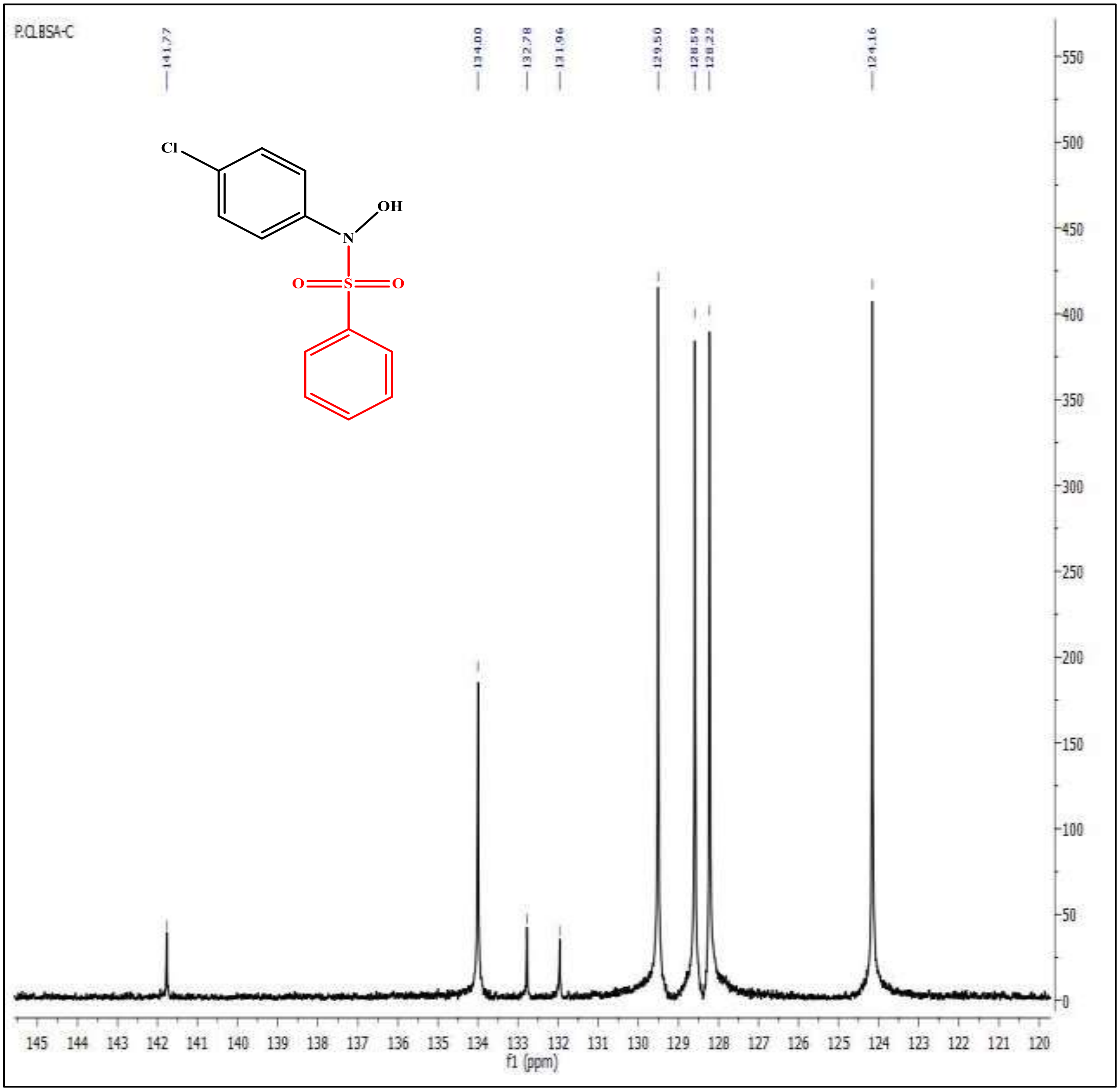

MS spectrum of 1c

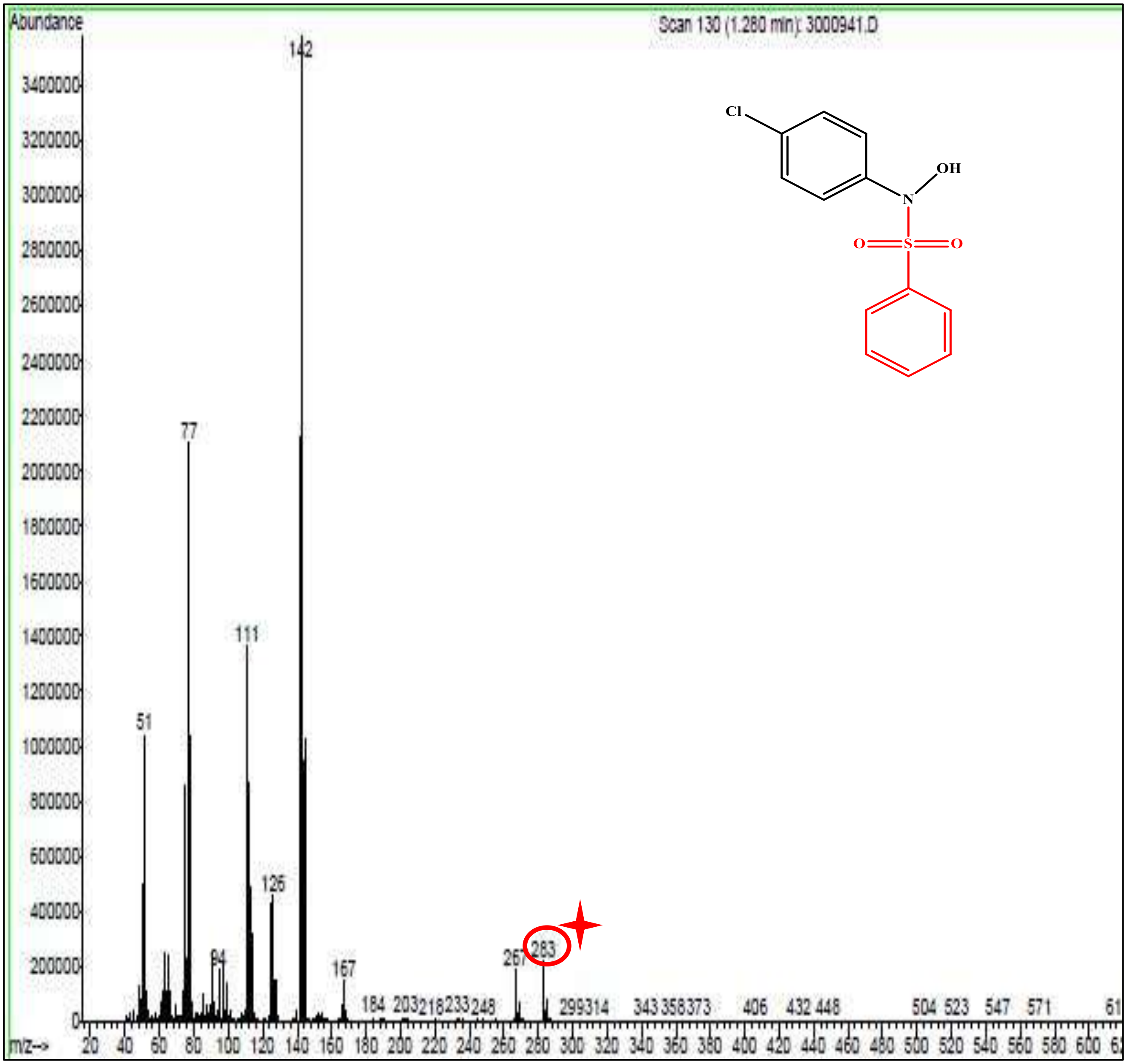

FT-IR spectrum of 2c

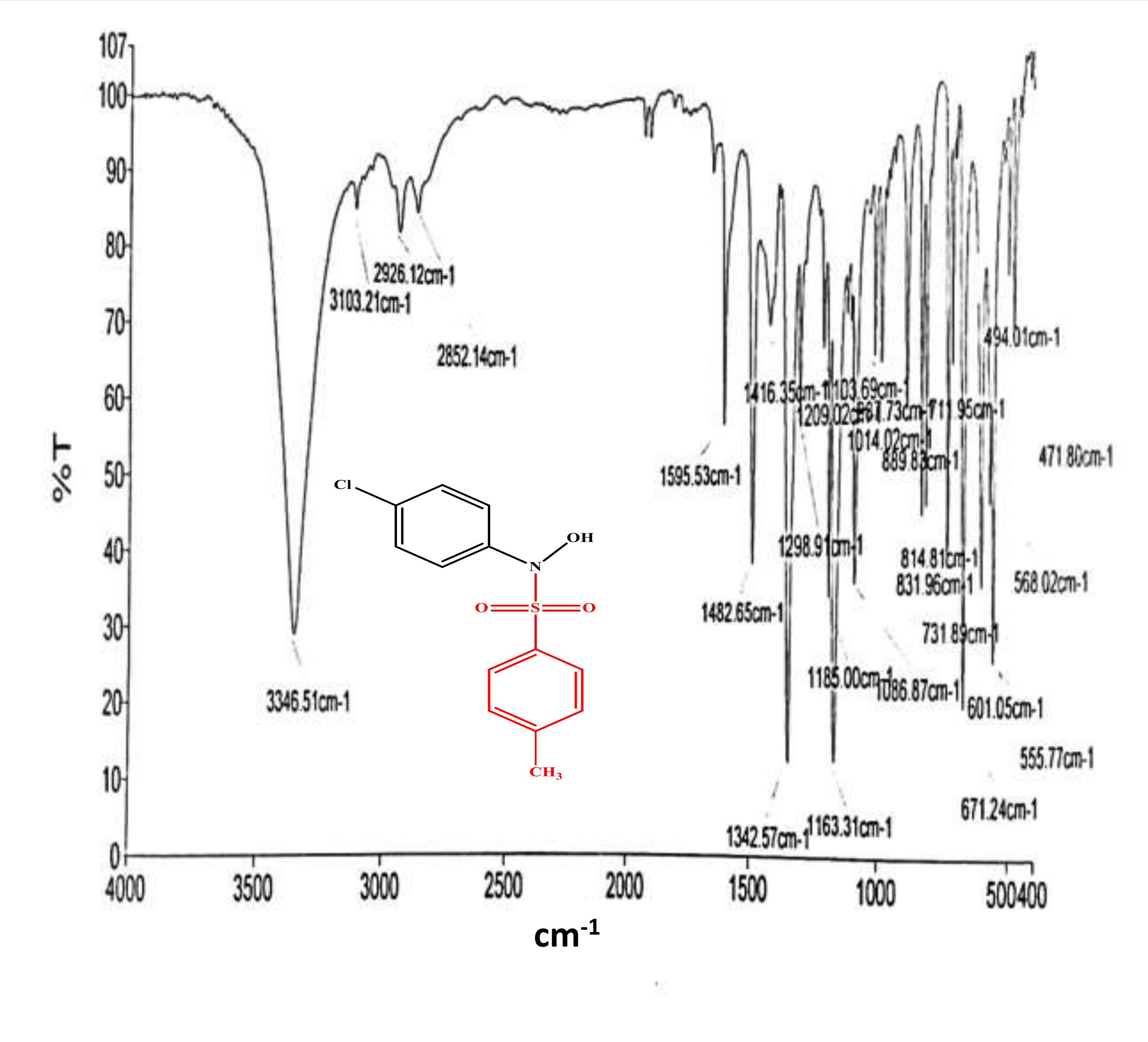

^{1}H NMR spectrum of 2c

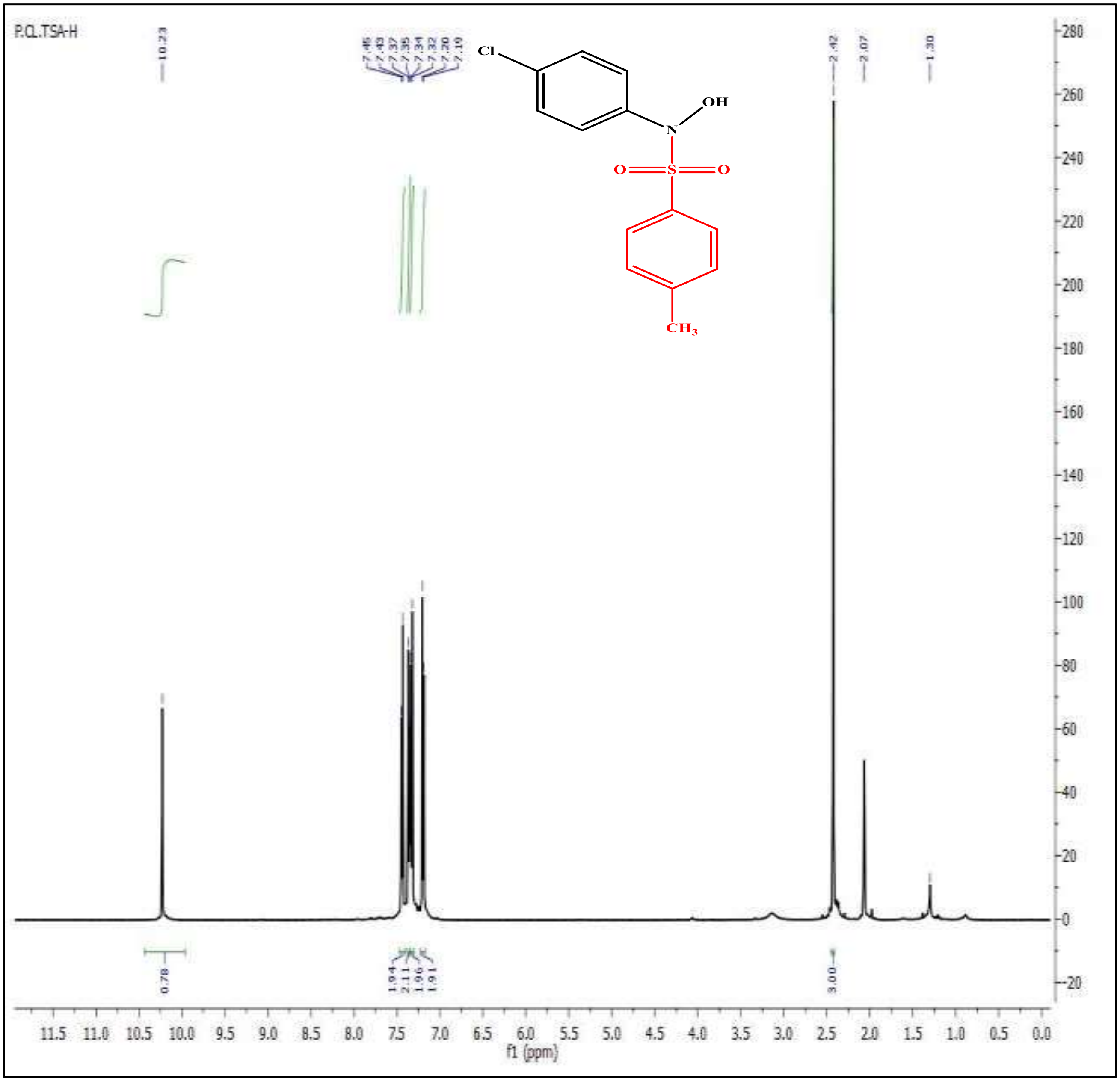

Expanded ^{1}H NMR spectrum of 2c

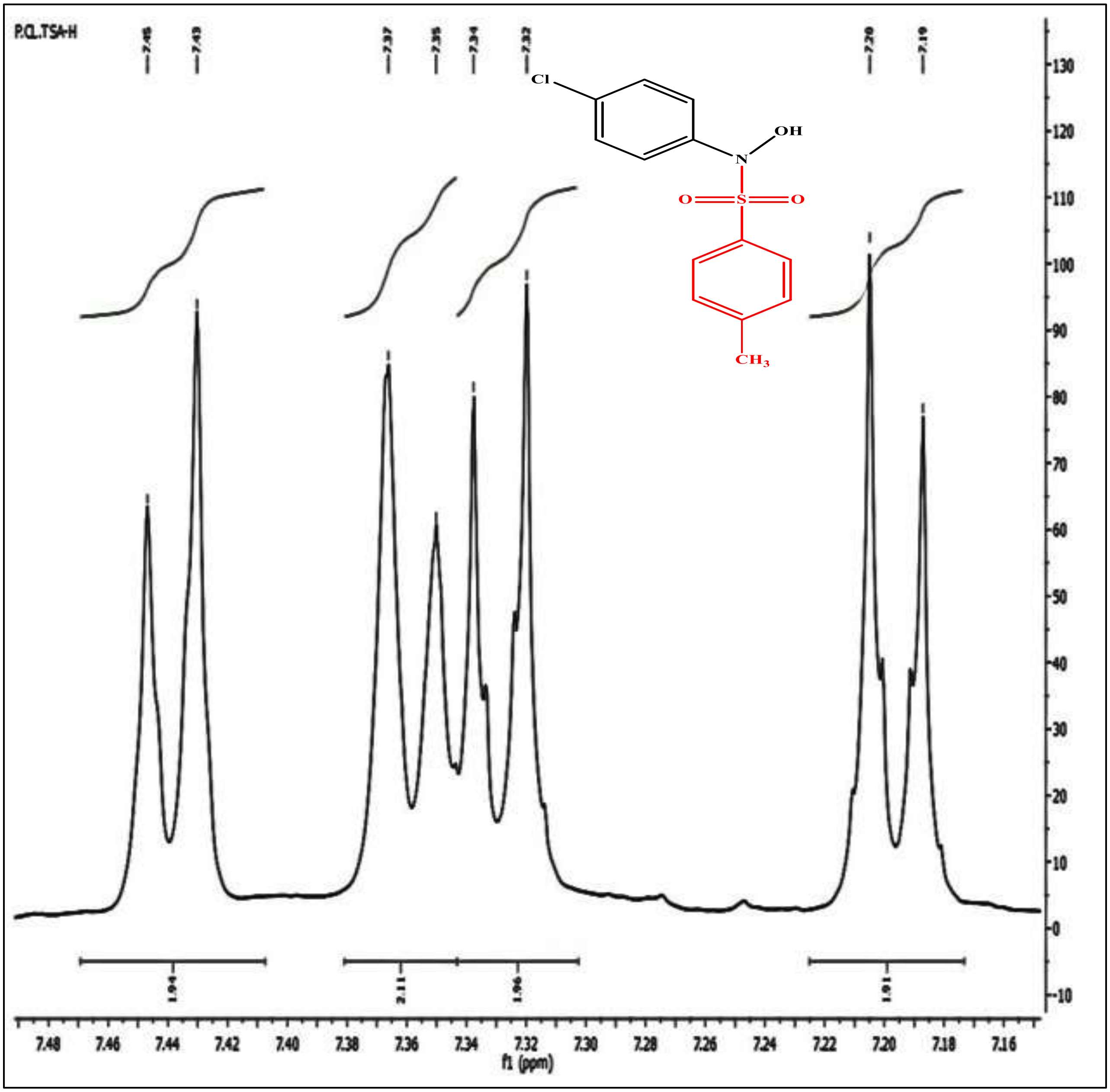

^{13}C NMR spectrum of 2c

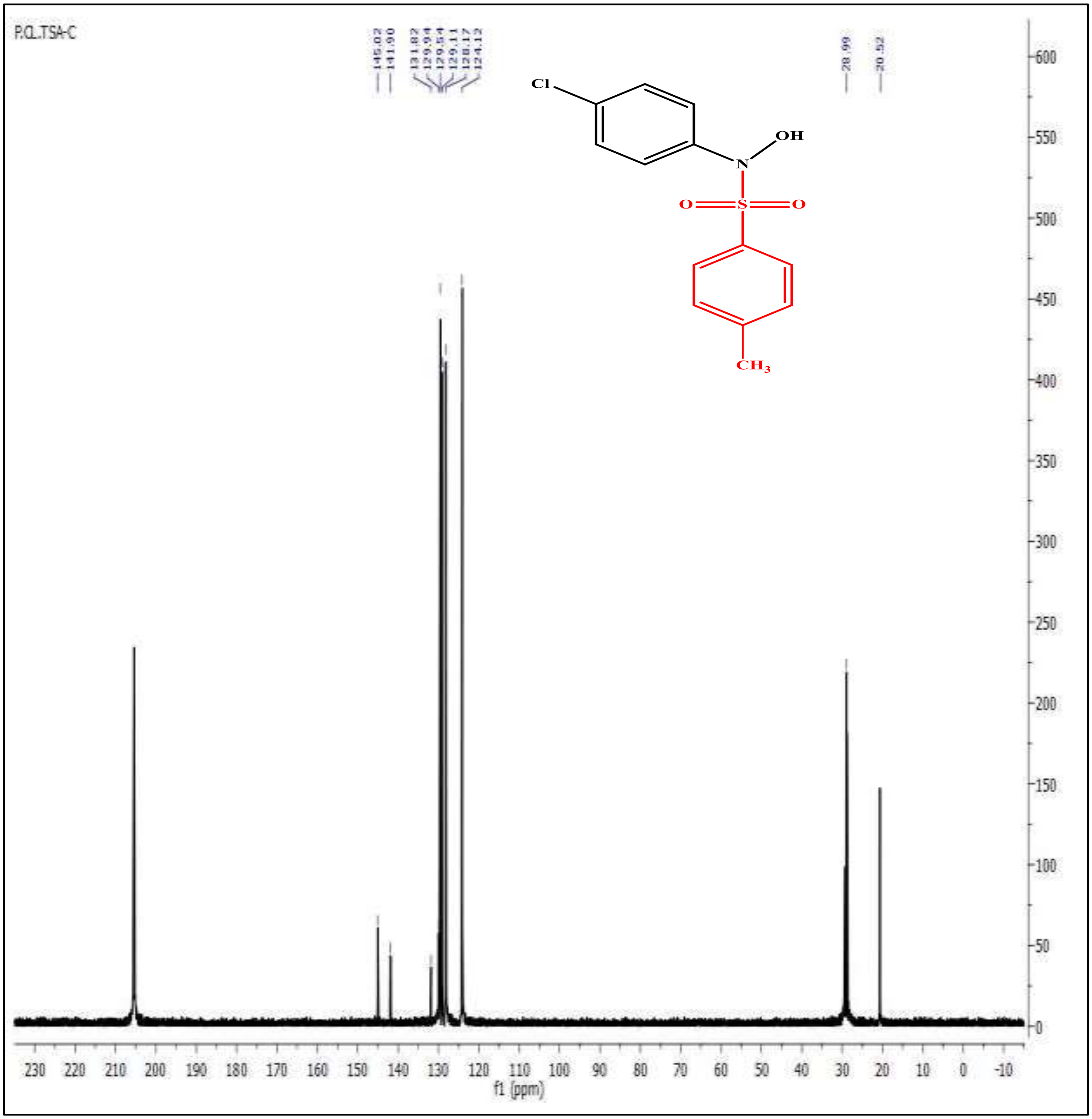

Expanded ^{13}C NMR spectrum of 3c

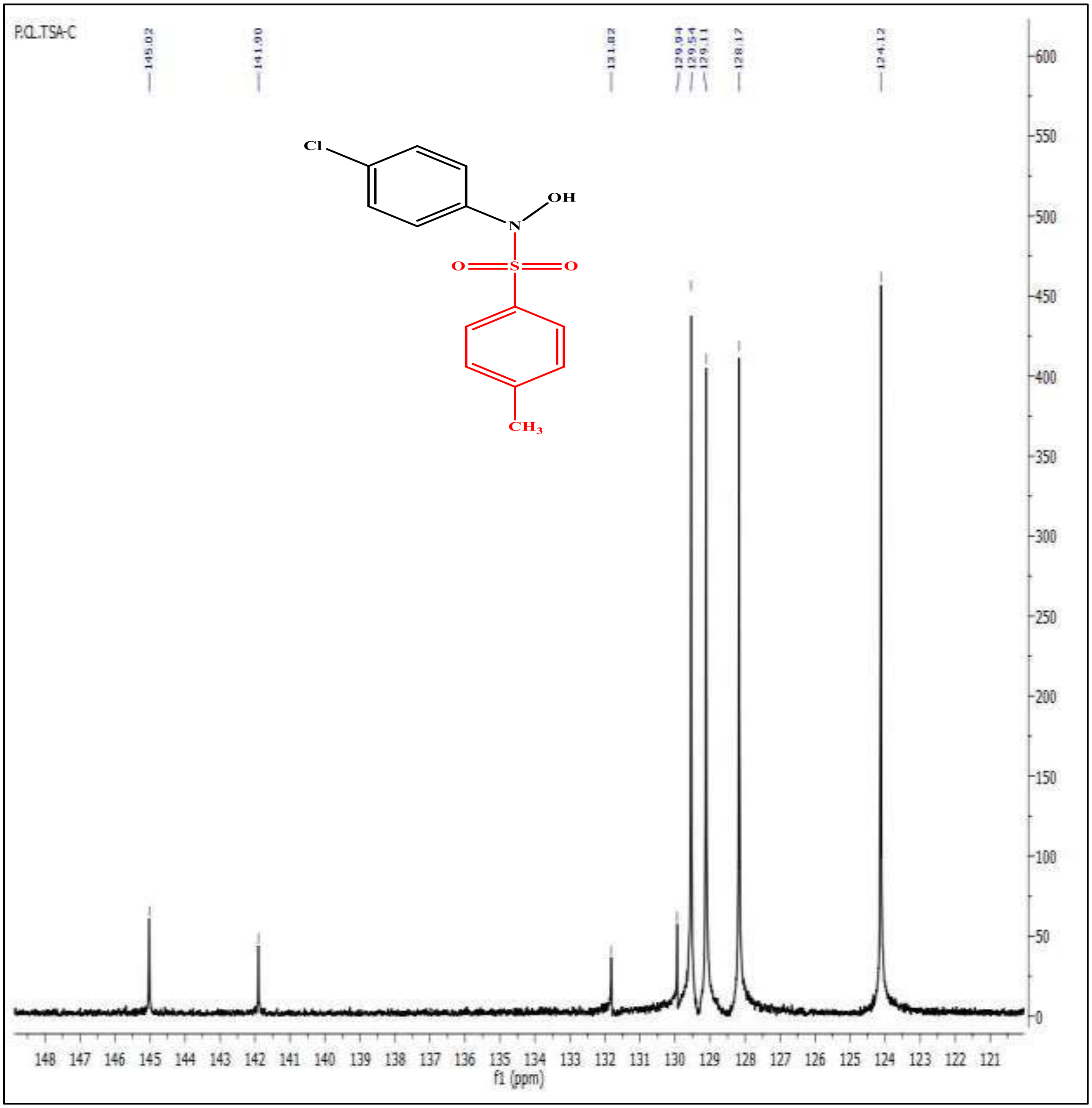

MS spectrum of 2c

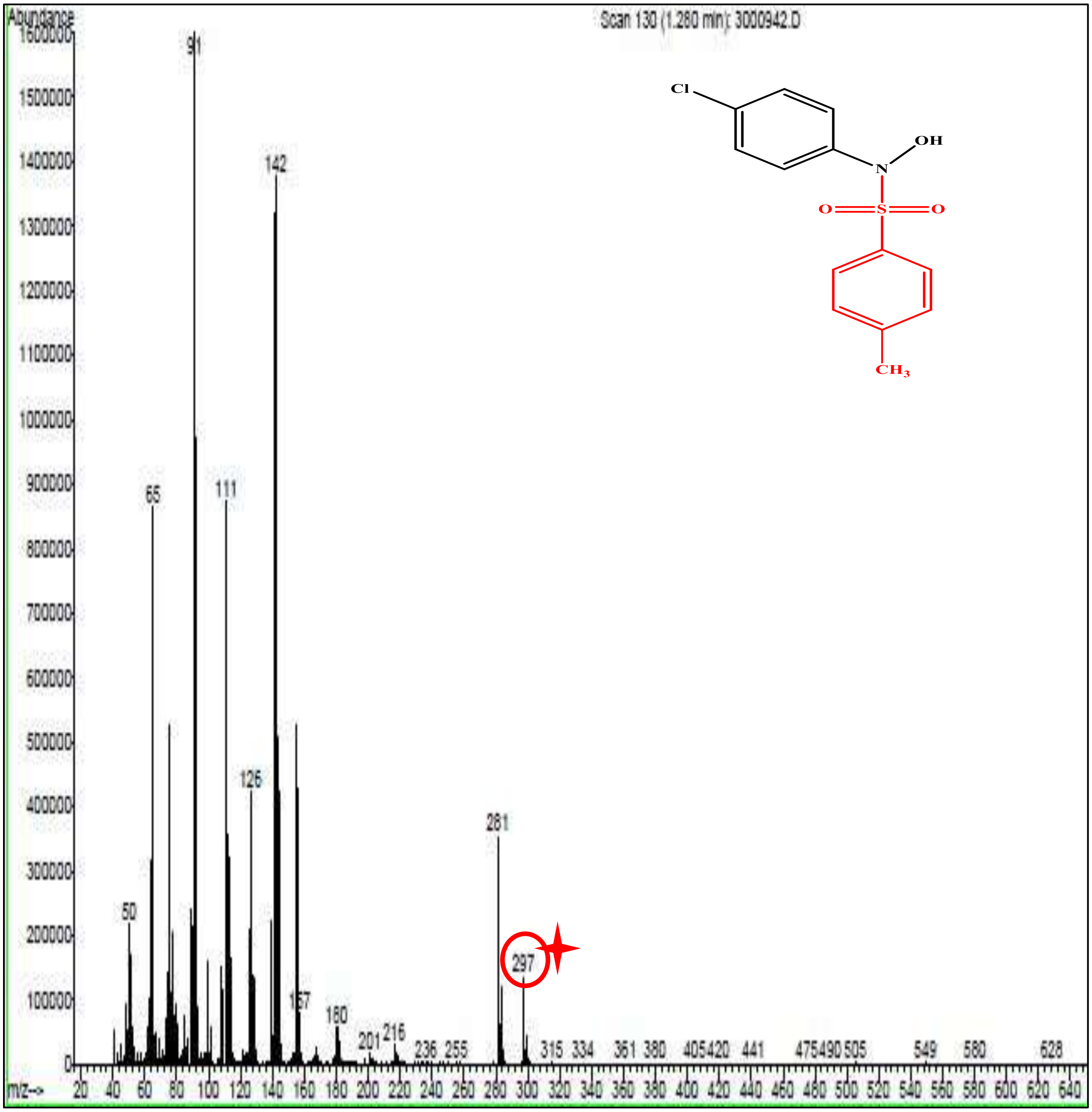

FT-IR spectrum of 3c

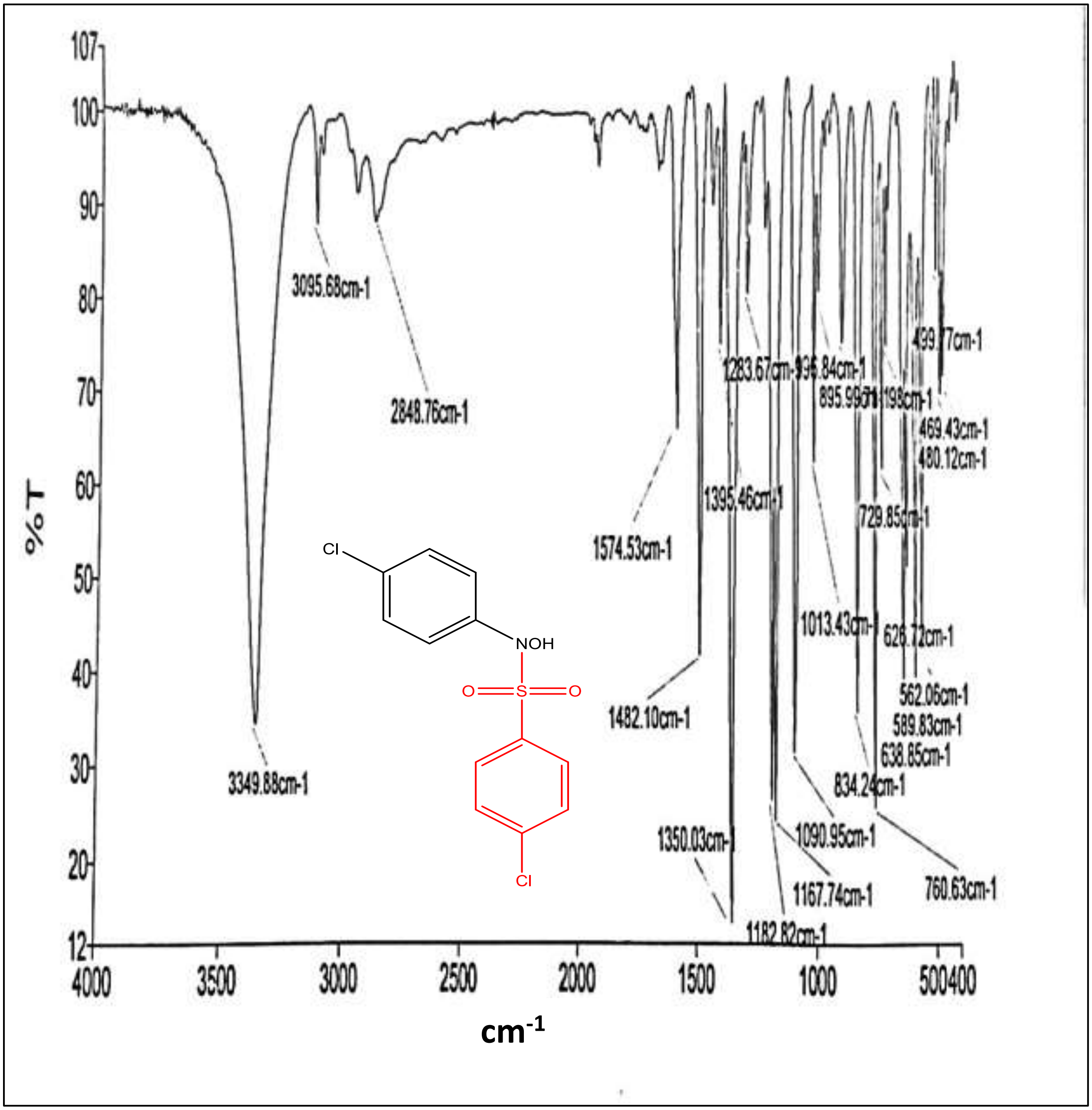

^{1}H NMR spectrum of 3c

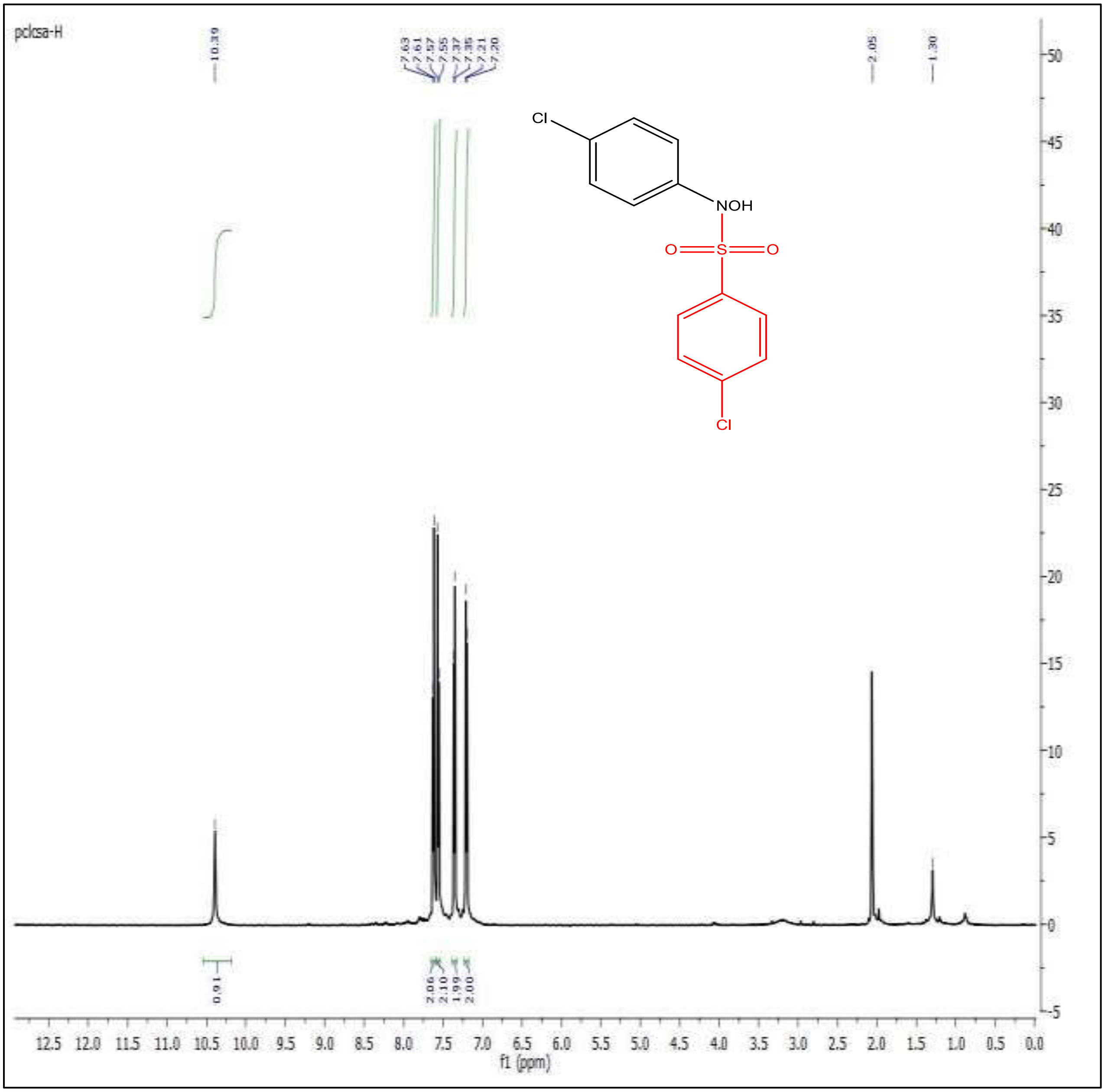

Expanded ^{1}H NMR spectrum of 3c

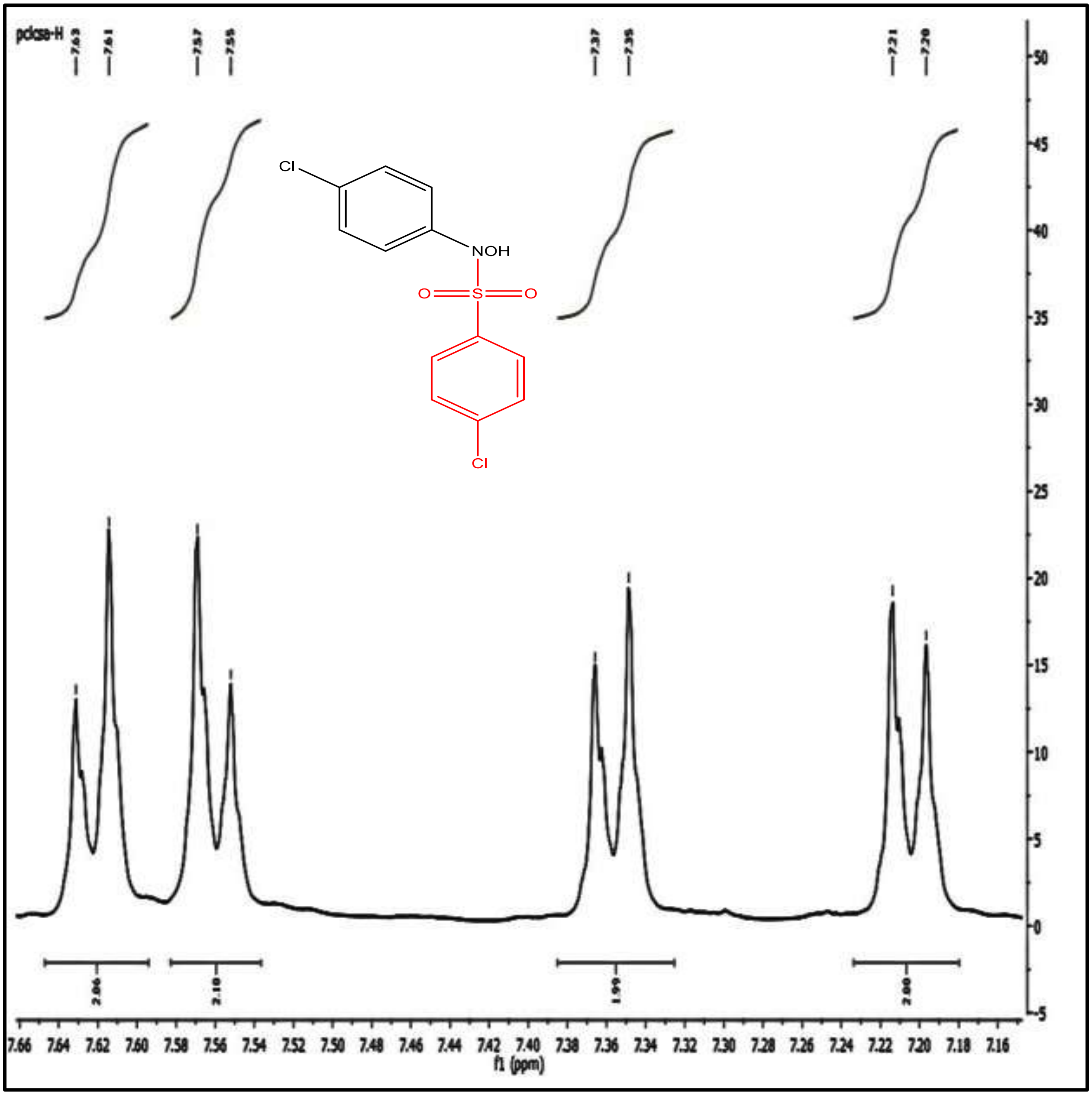

^{13}C NMR spectrum of 3c

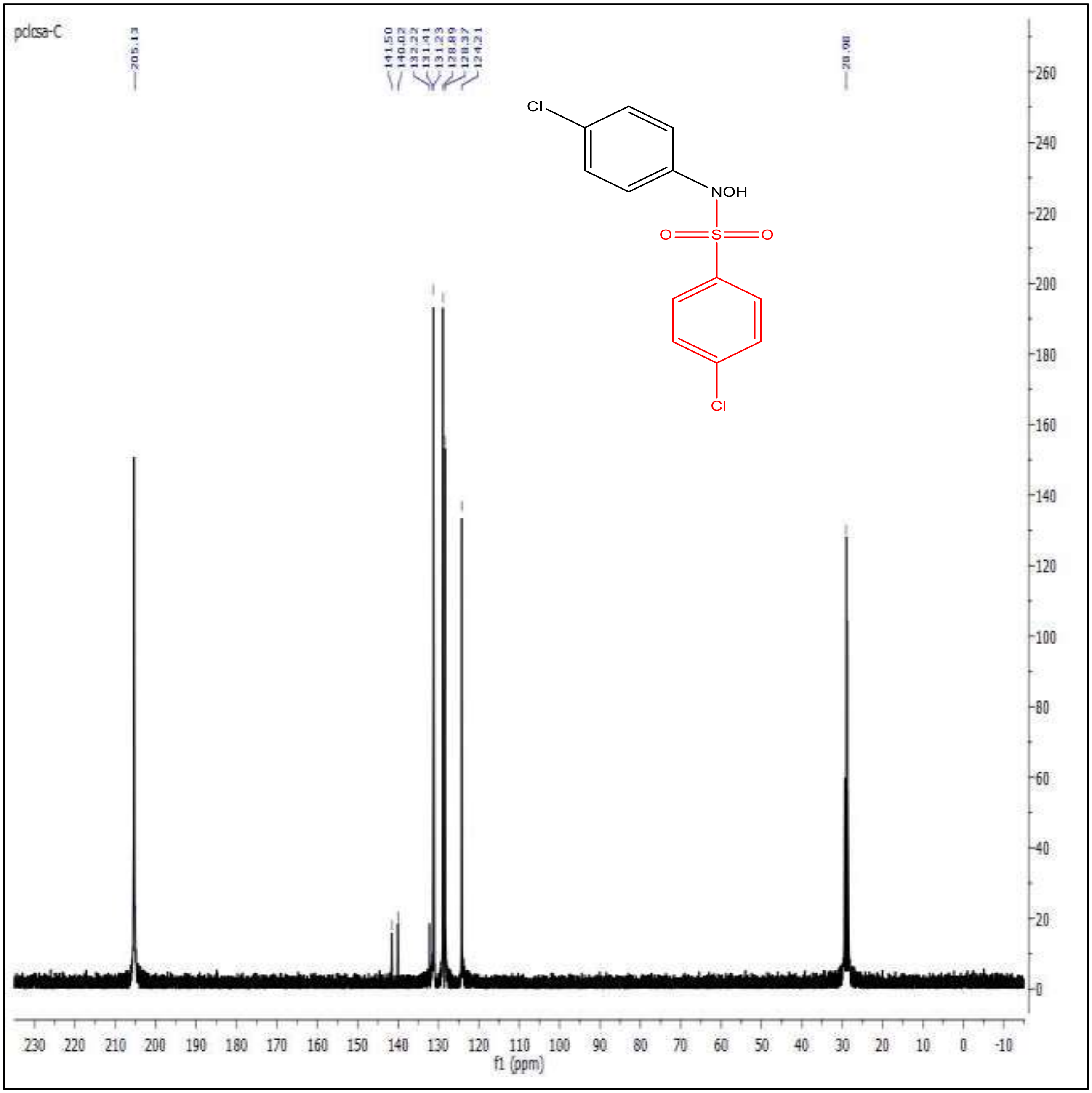

Expanded ^{13}C NMR spectrum of 3c

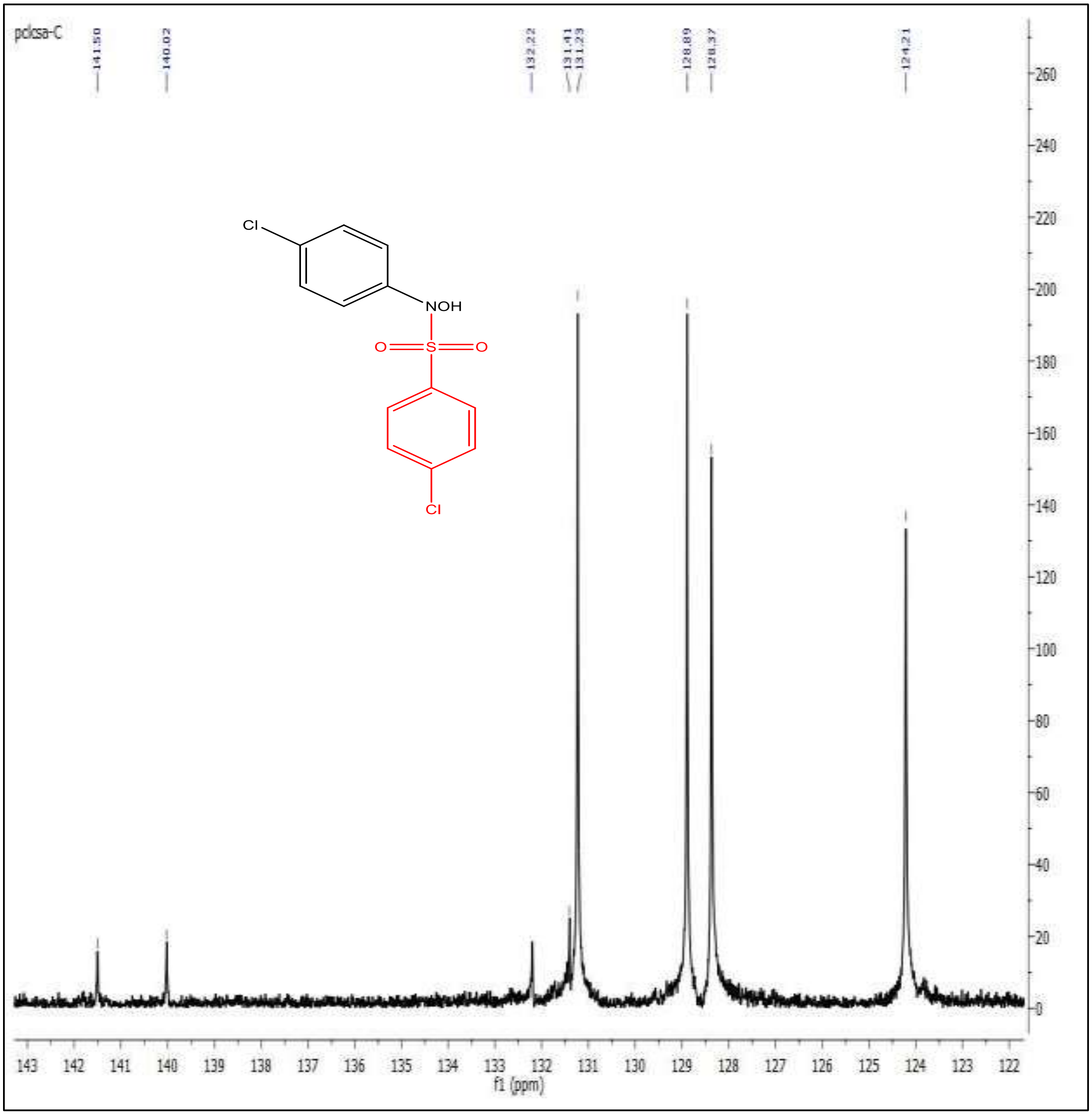

MS spectrum of 3c

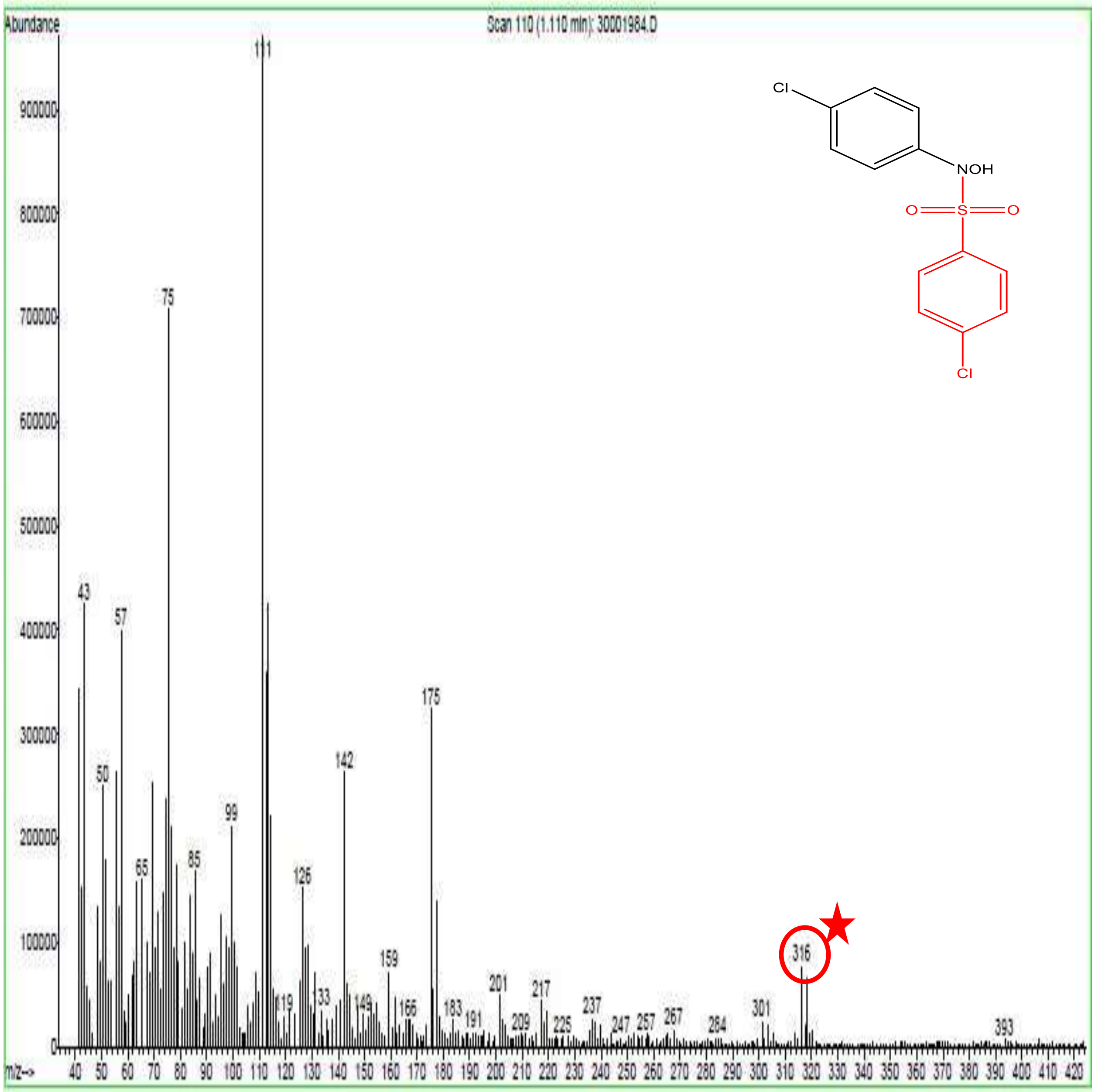

FT-IR spectrum of 1d

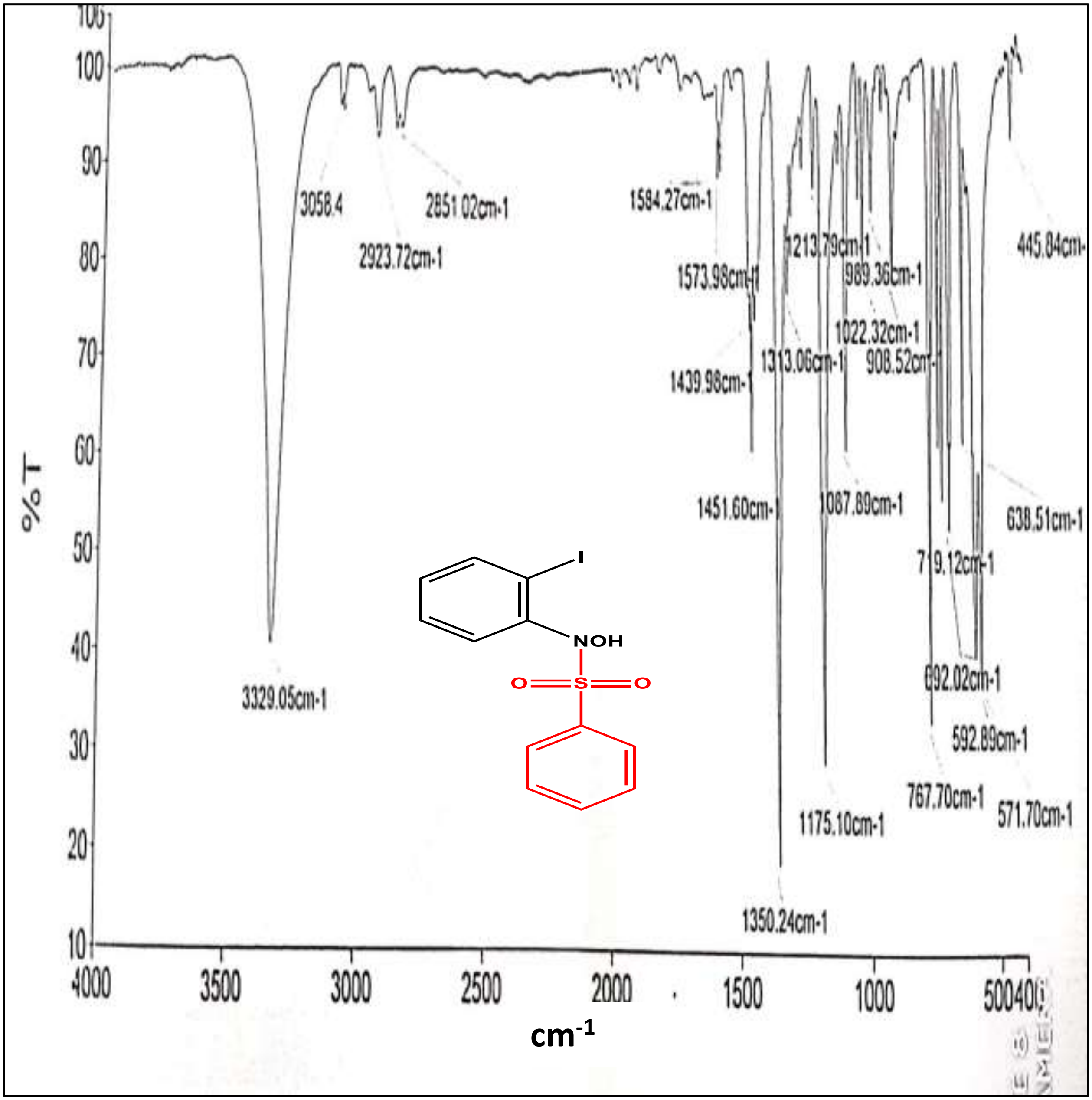

^{1}H NMR spectrum of 1d

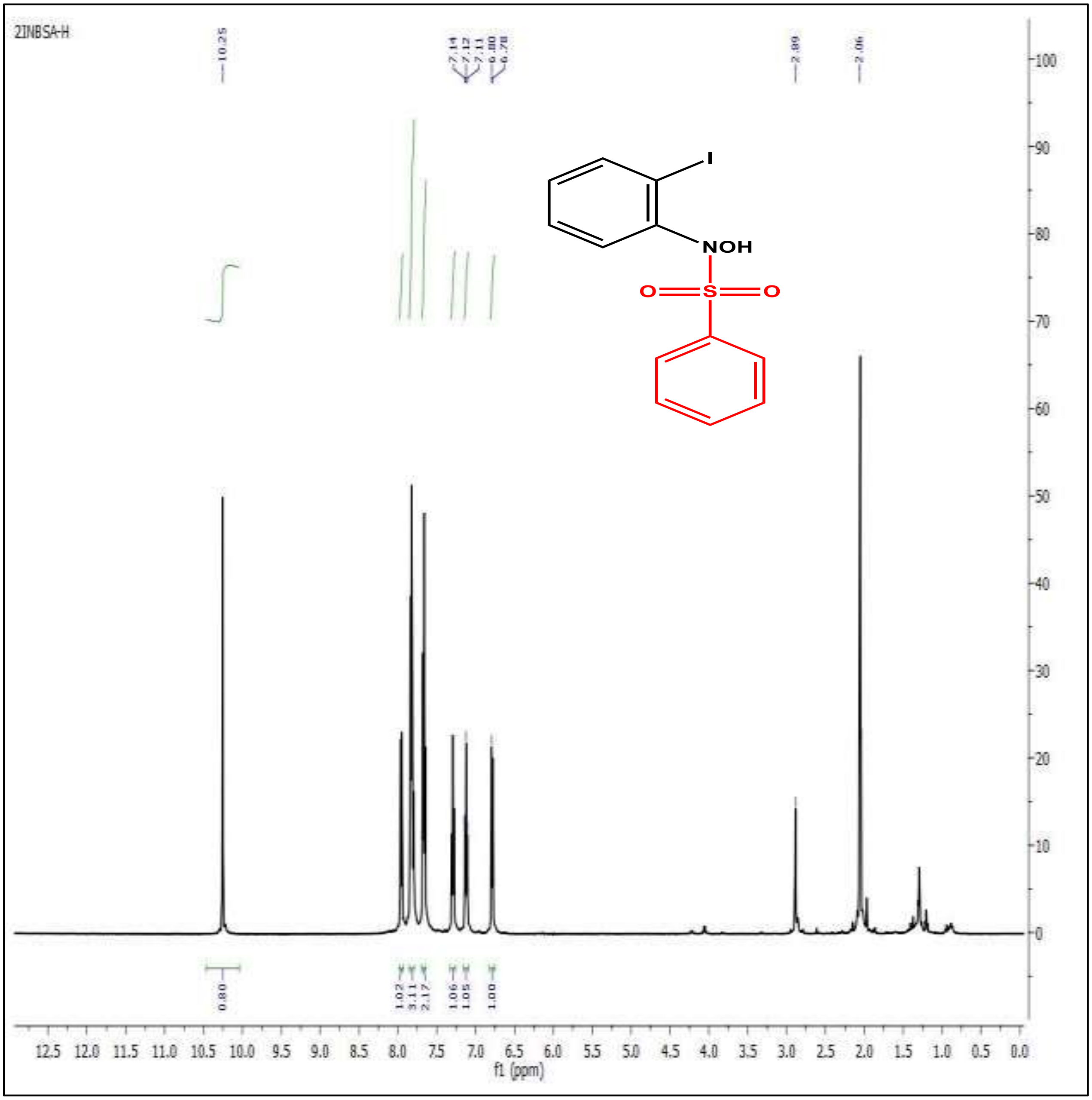

Expanded ^{1}H NMR spectrum of 1d

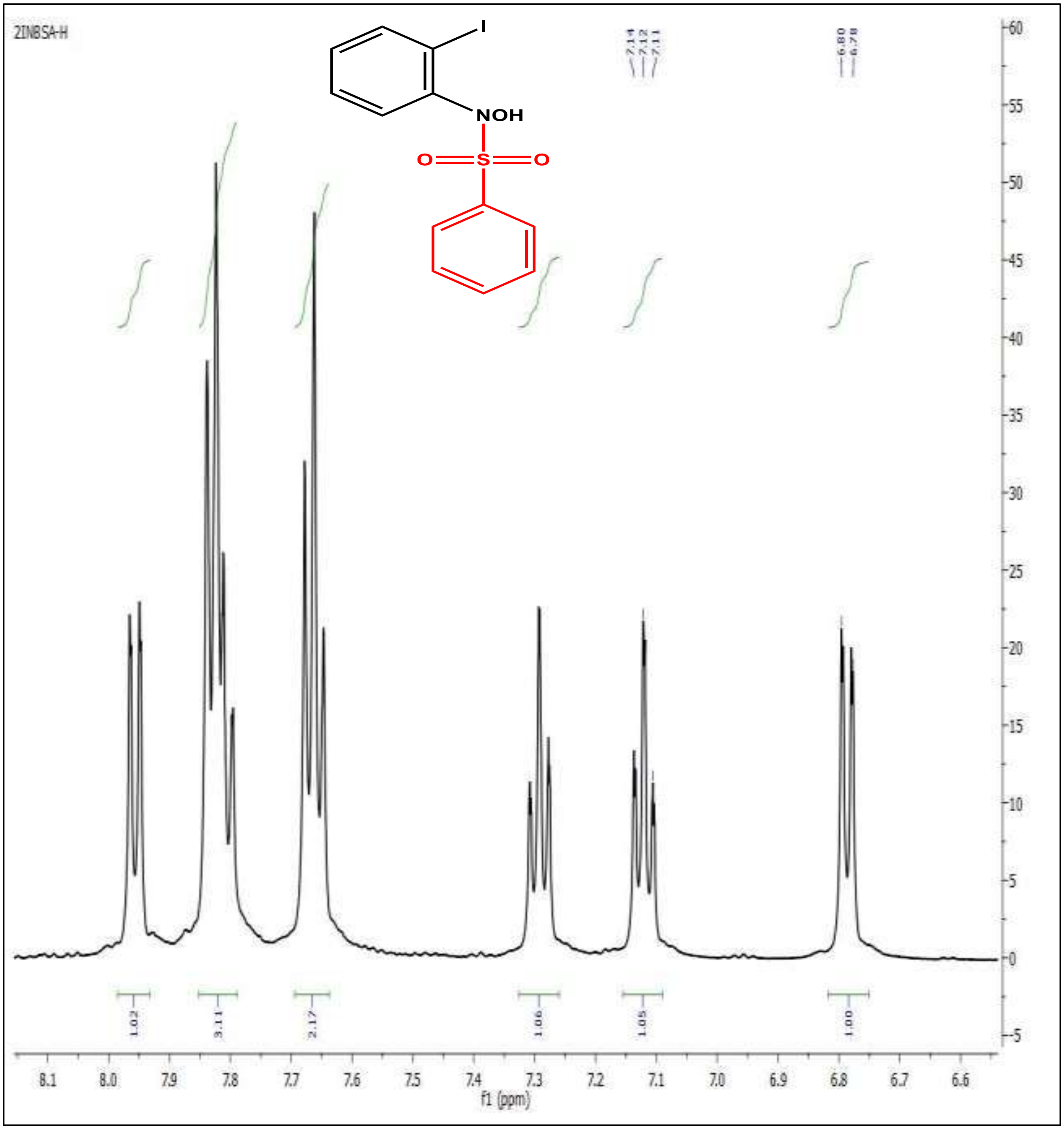

^{13}C NMR spectrum of 1d

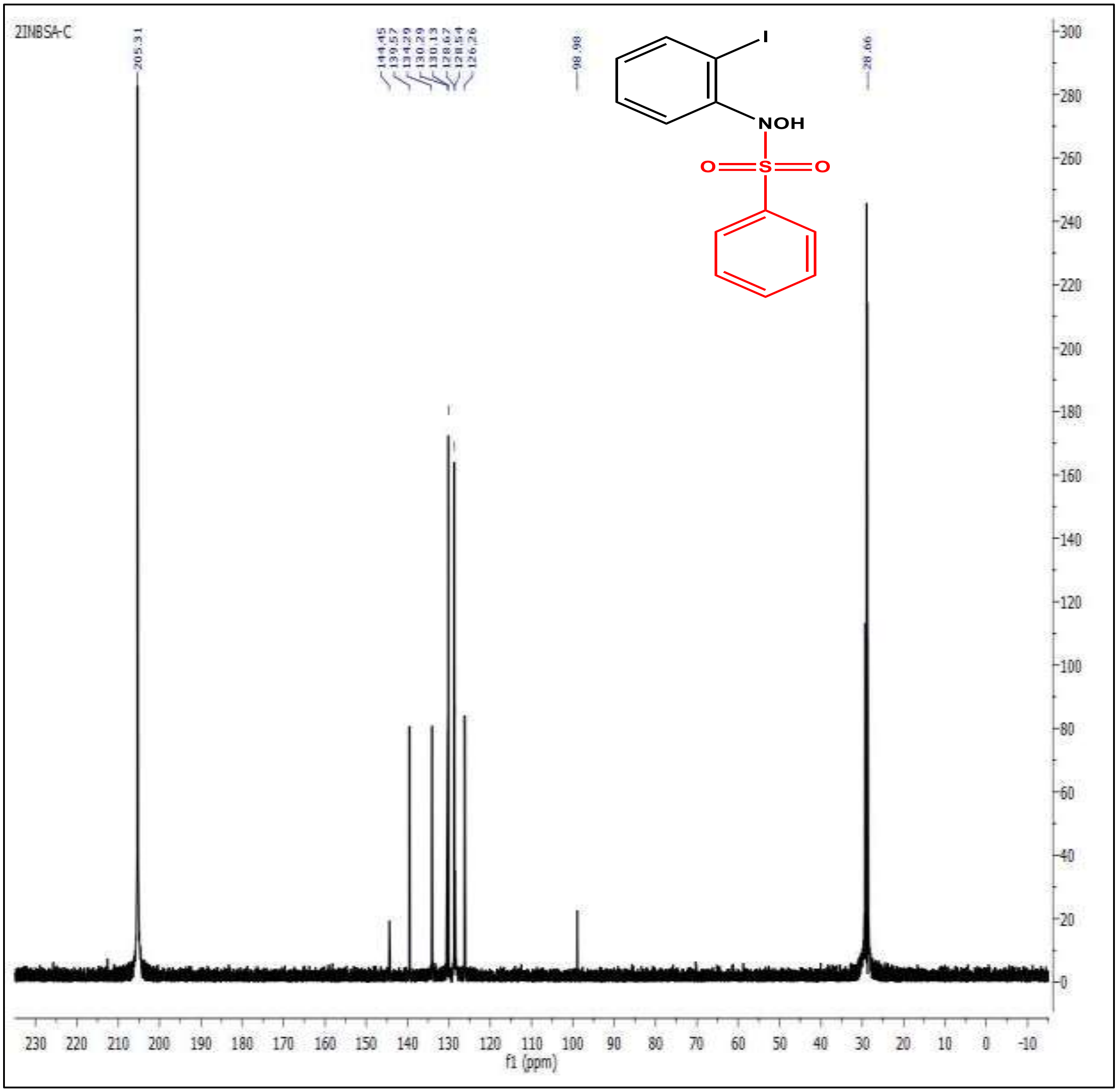

Expanded ^{13}C NMR spectrum of 1d

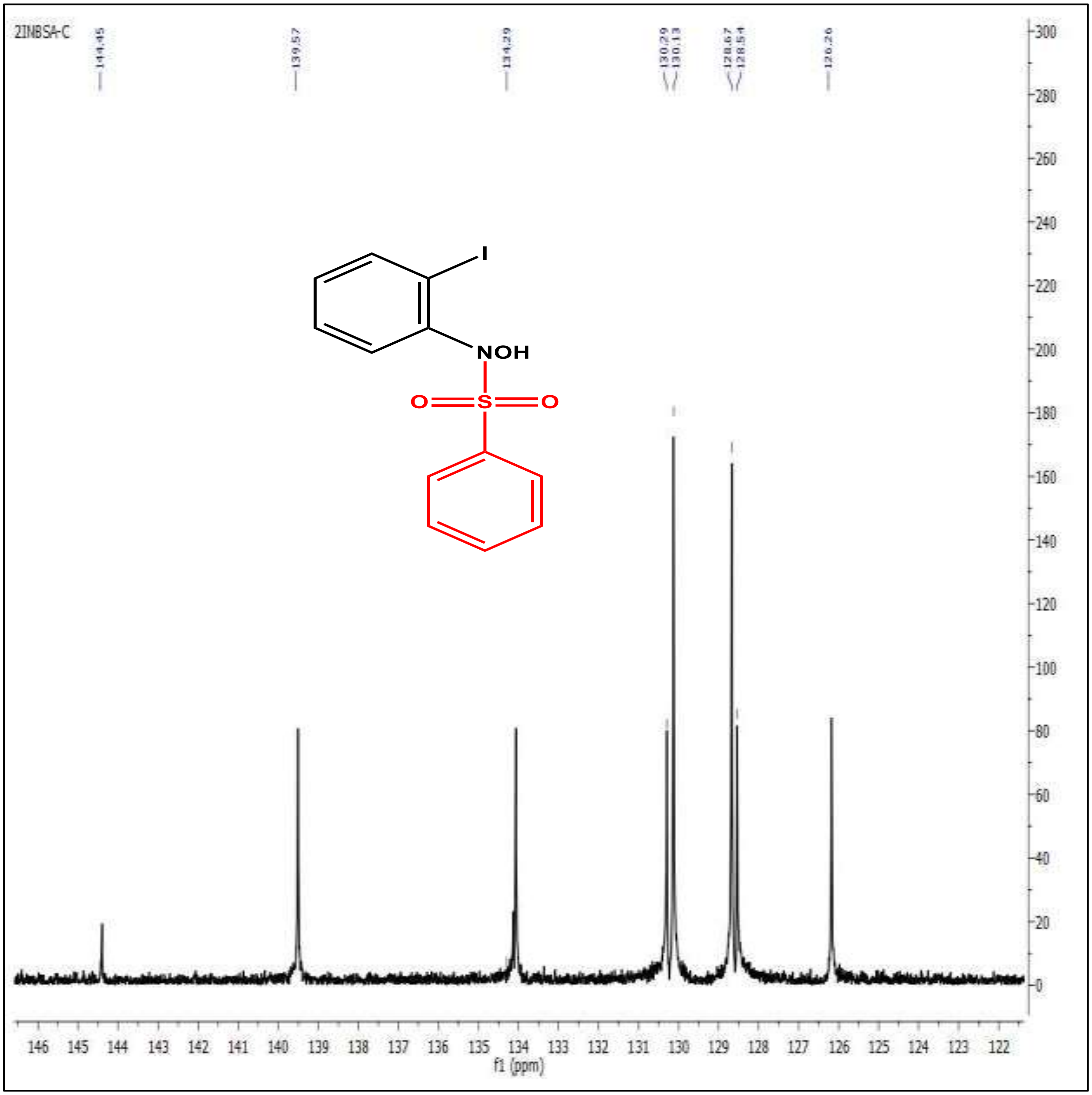

MS spectrum of 1d

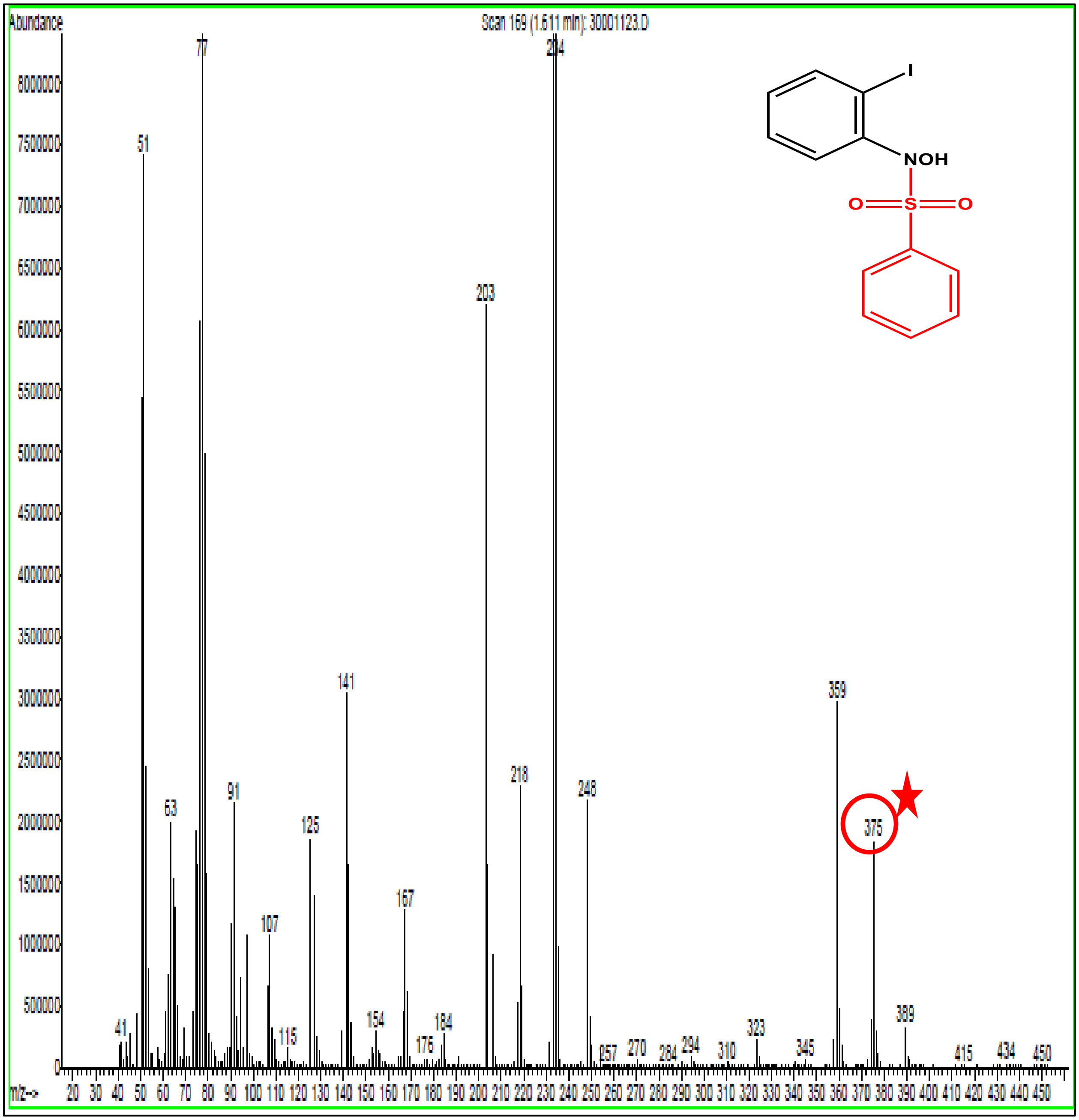

FT-IR spectrum of 2d

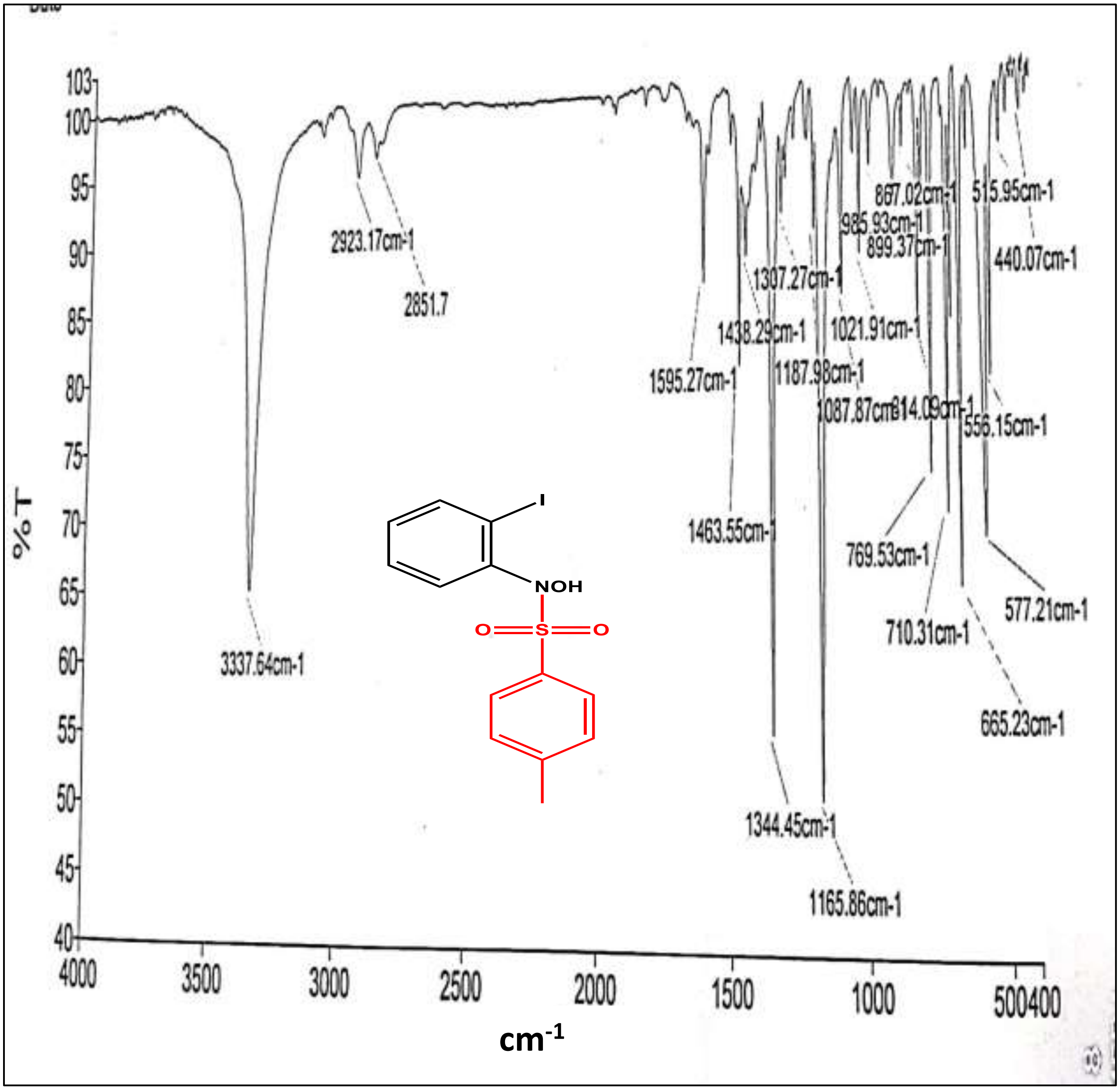

^{1}H NMR spectrum of 2d

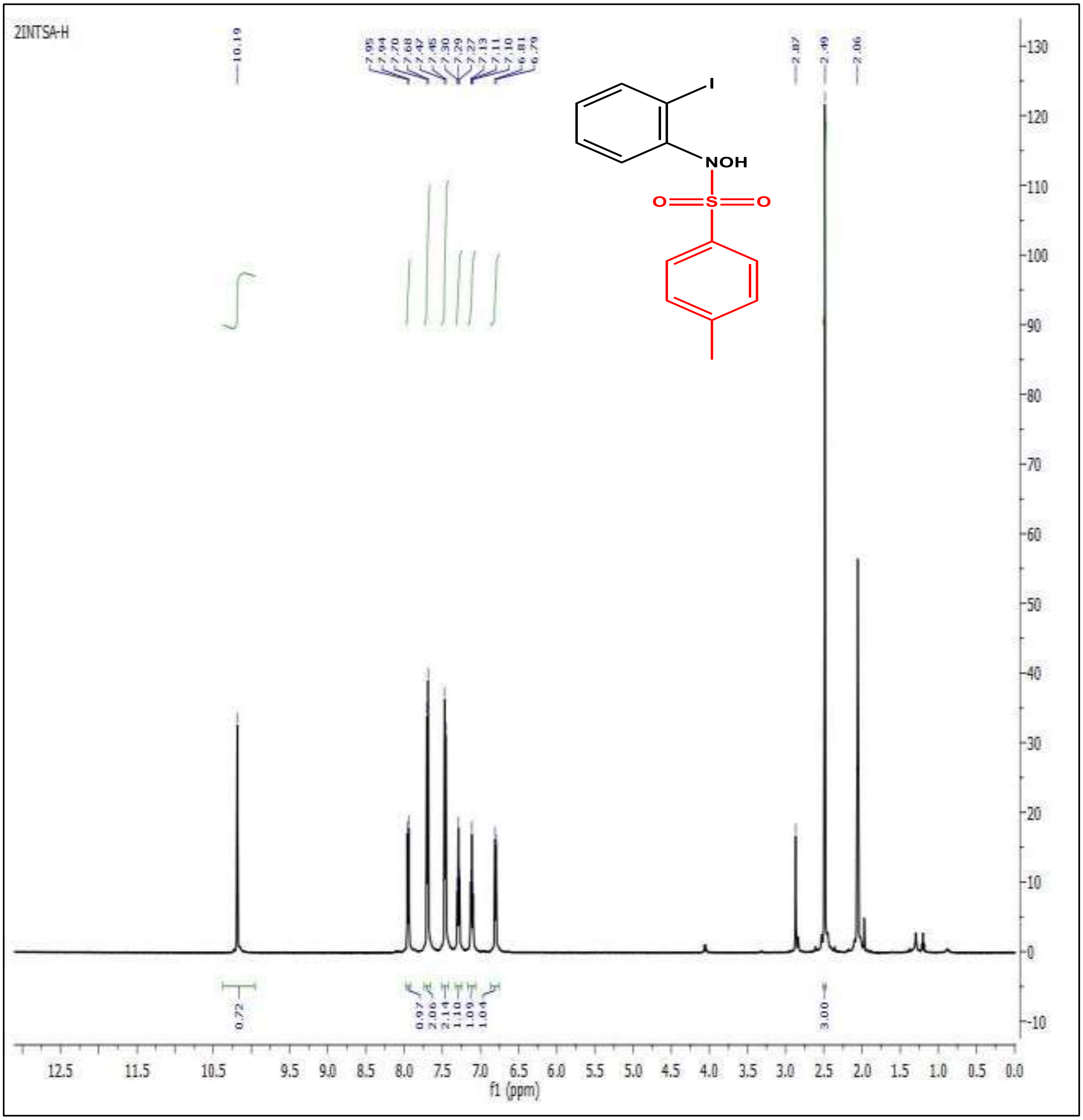

Expanded ^{1}H NMR spectrum of 2d

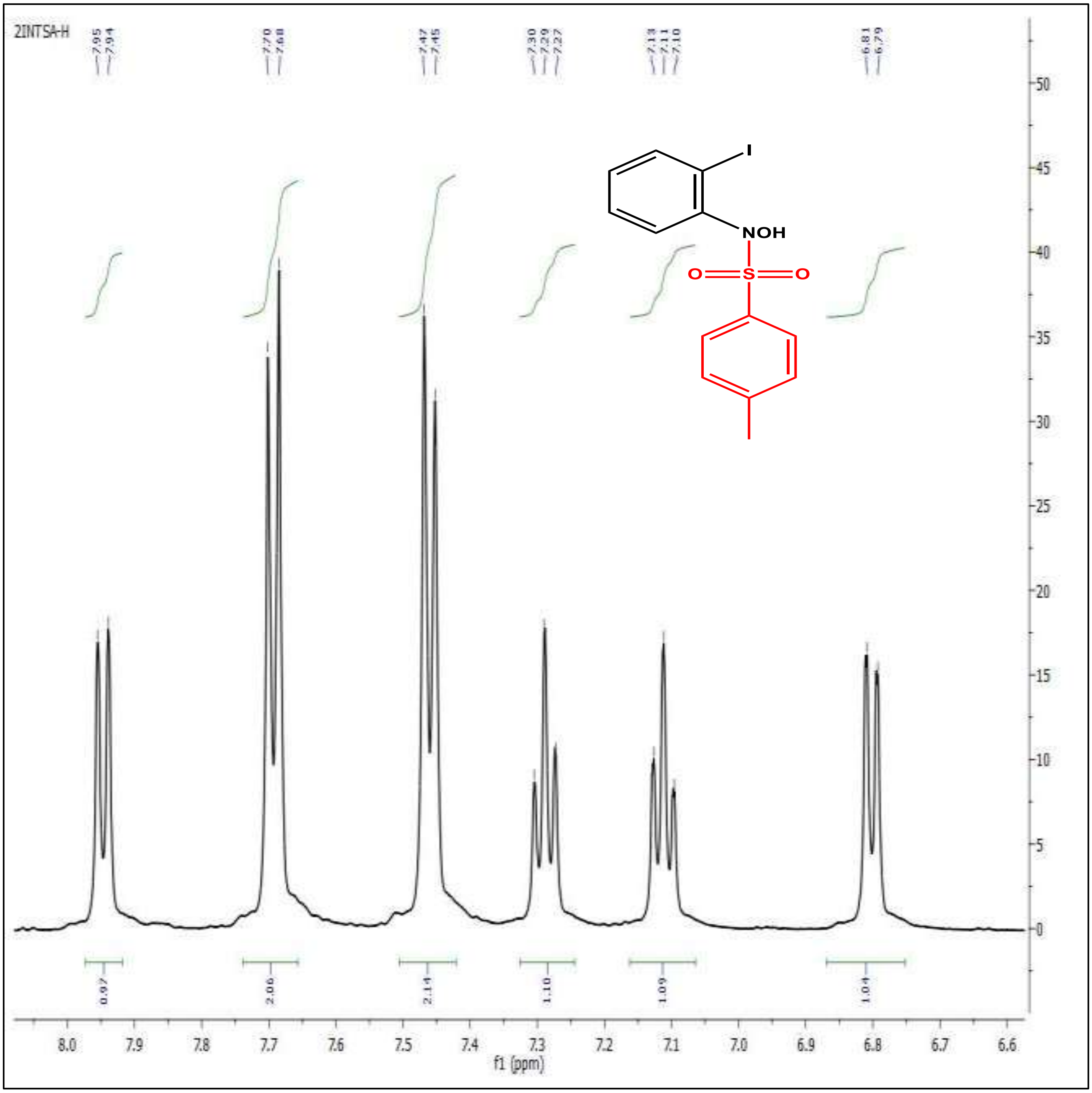

^{13}C NMR spectrum of 2d

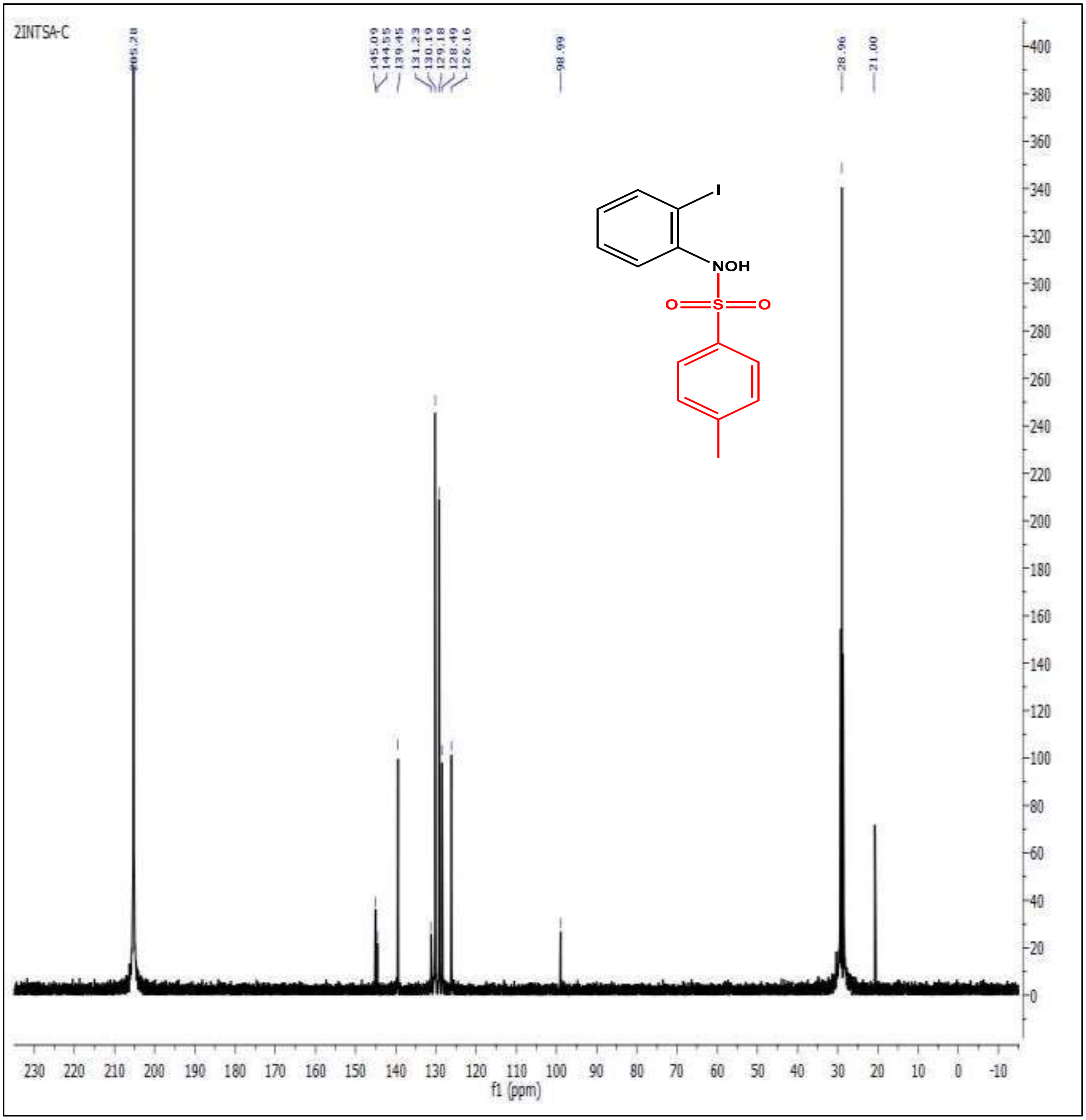

Expanded ^{13}C NMR spectrum of 2d

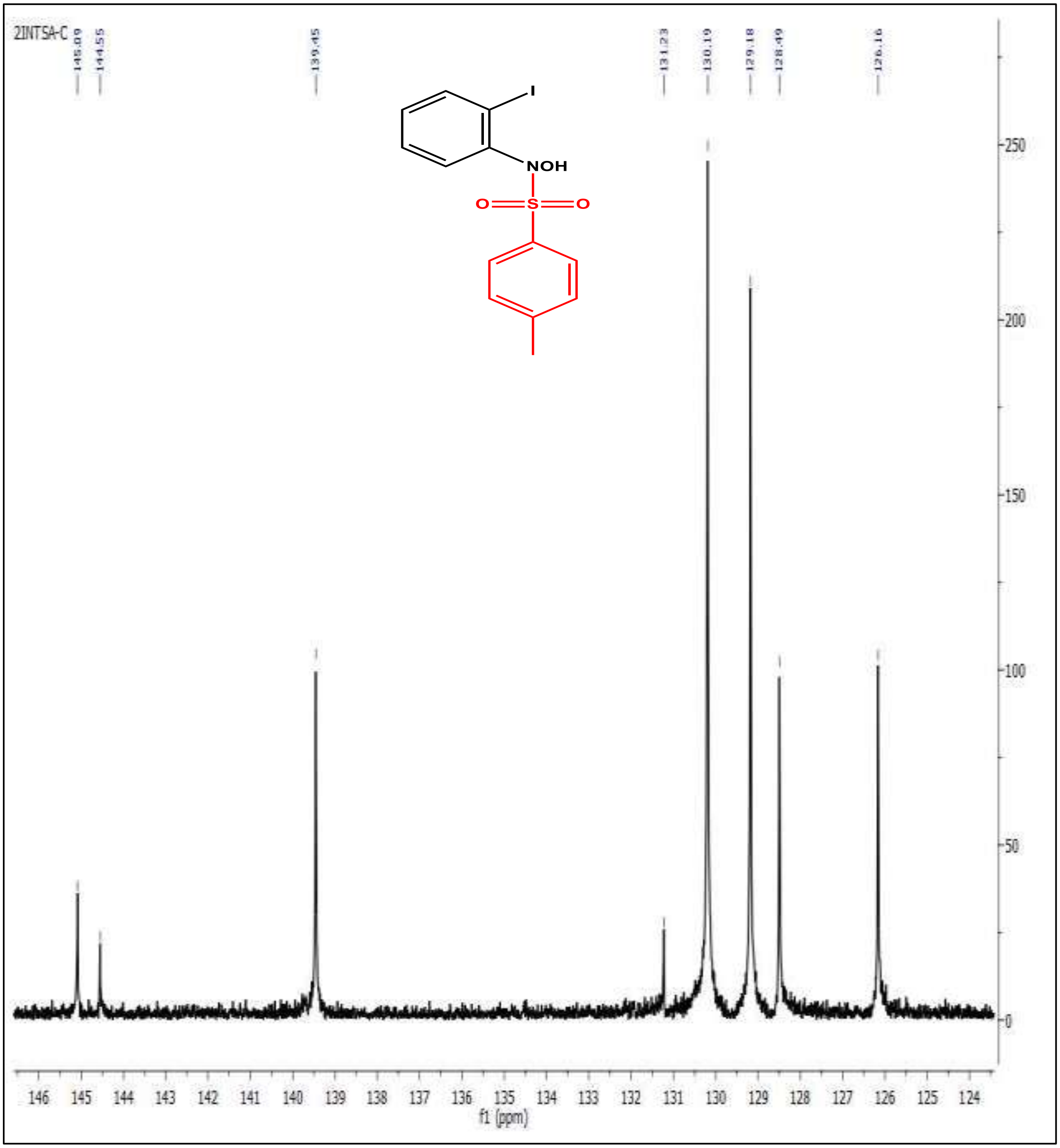

MS spectrum of 2d

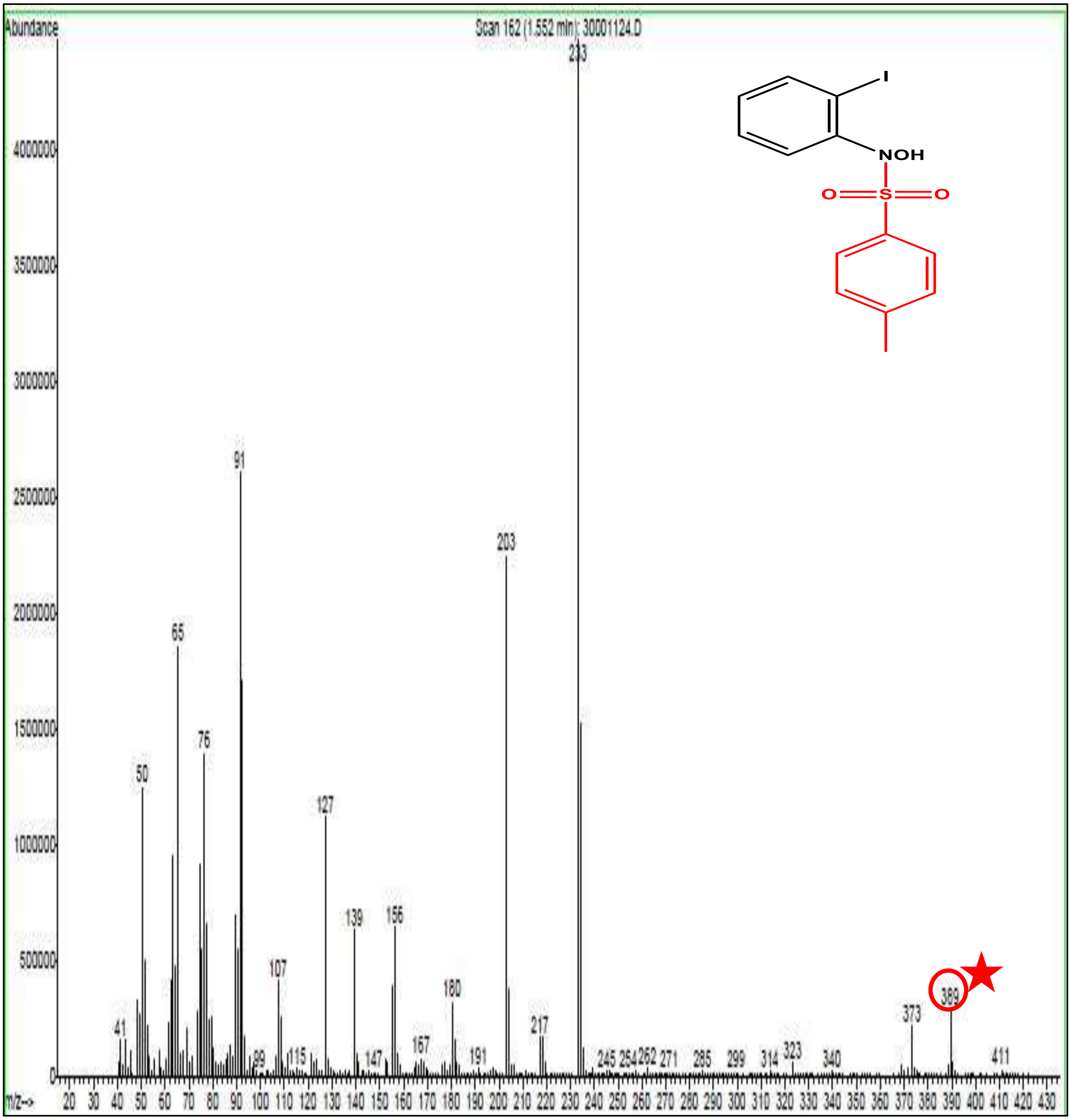

FT-IR spectrum of 3d

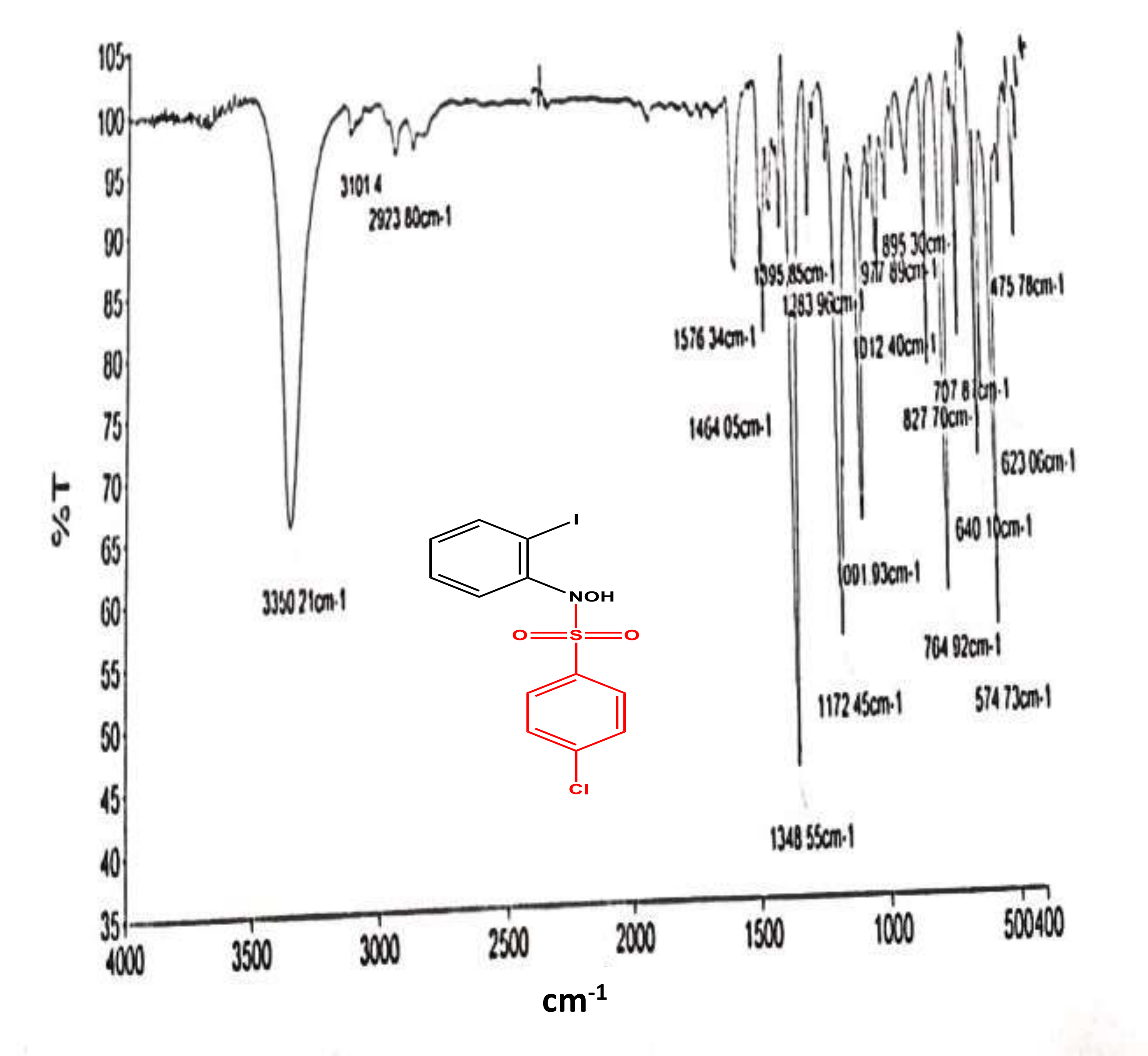

^{1}H NMR spectrum of 3d

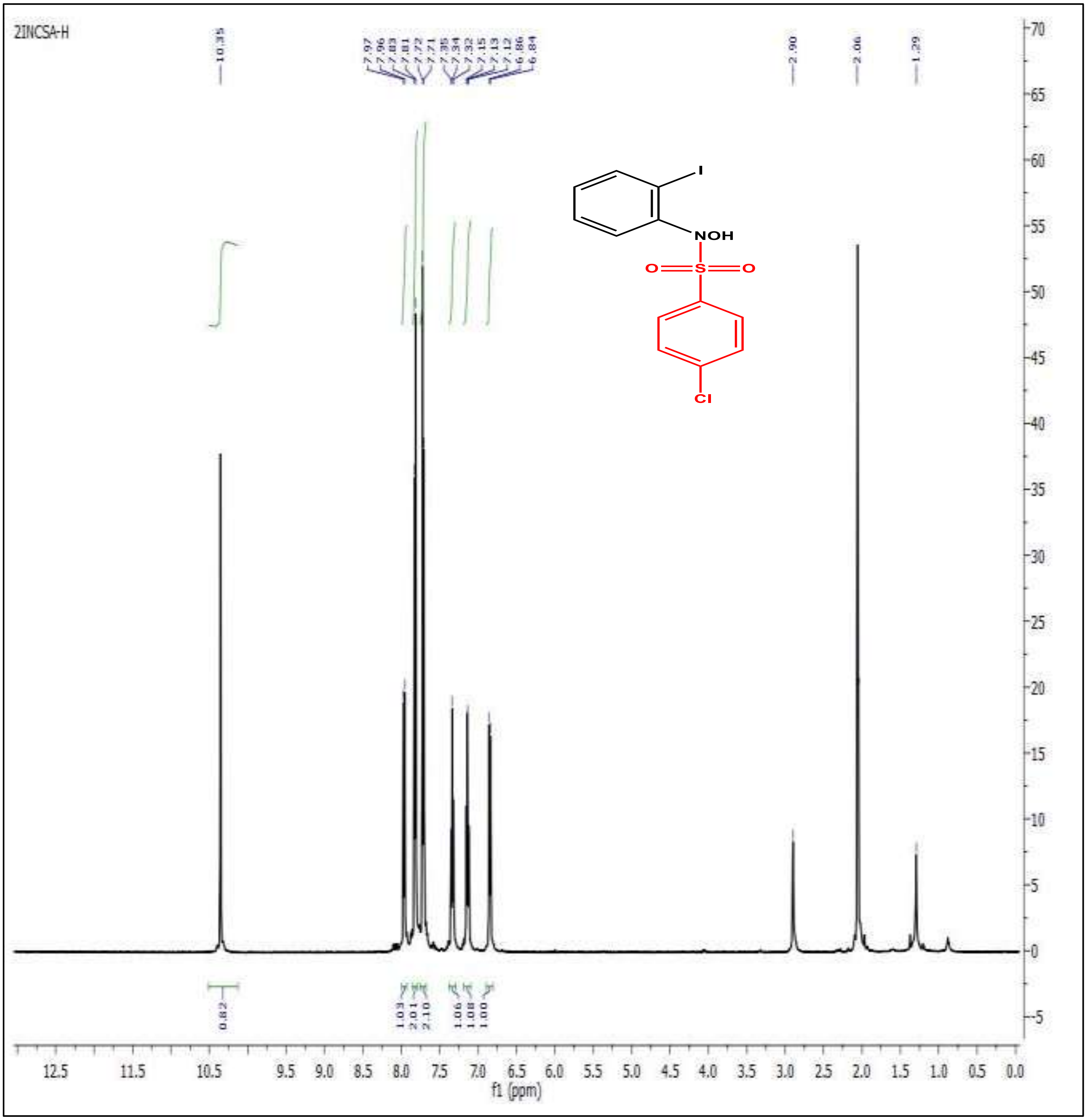

Expanded ^{1}H NMR spectrum of 3d

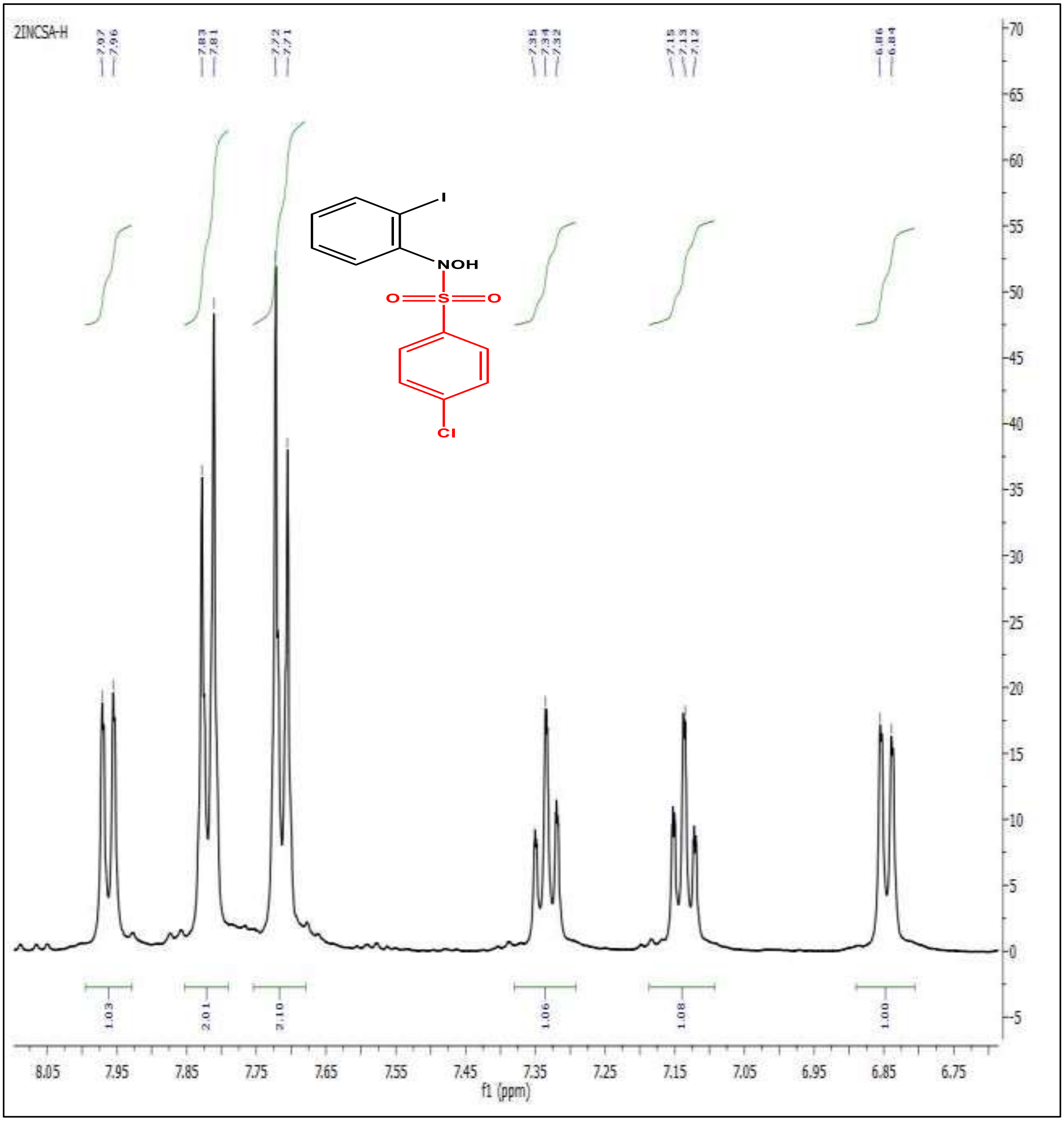

^{13}C NMR spectrum of 3d

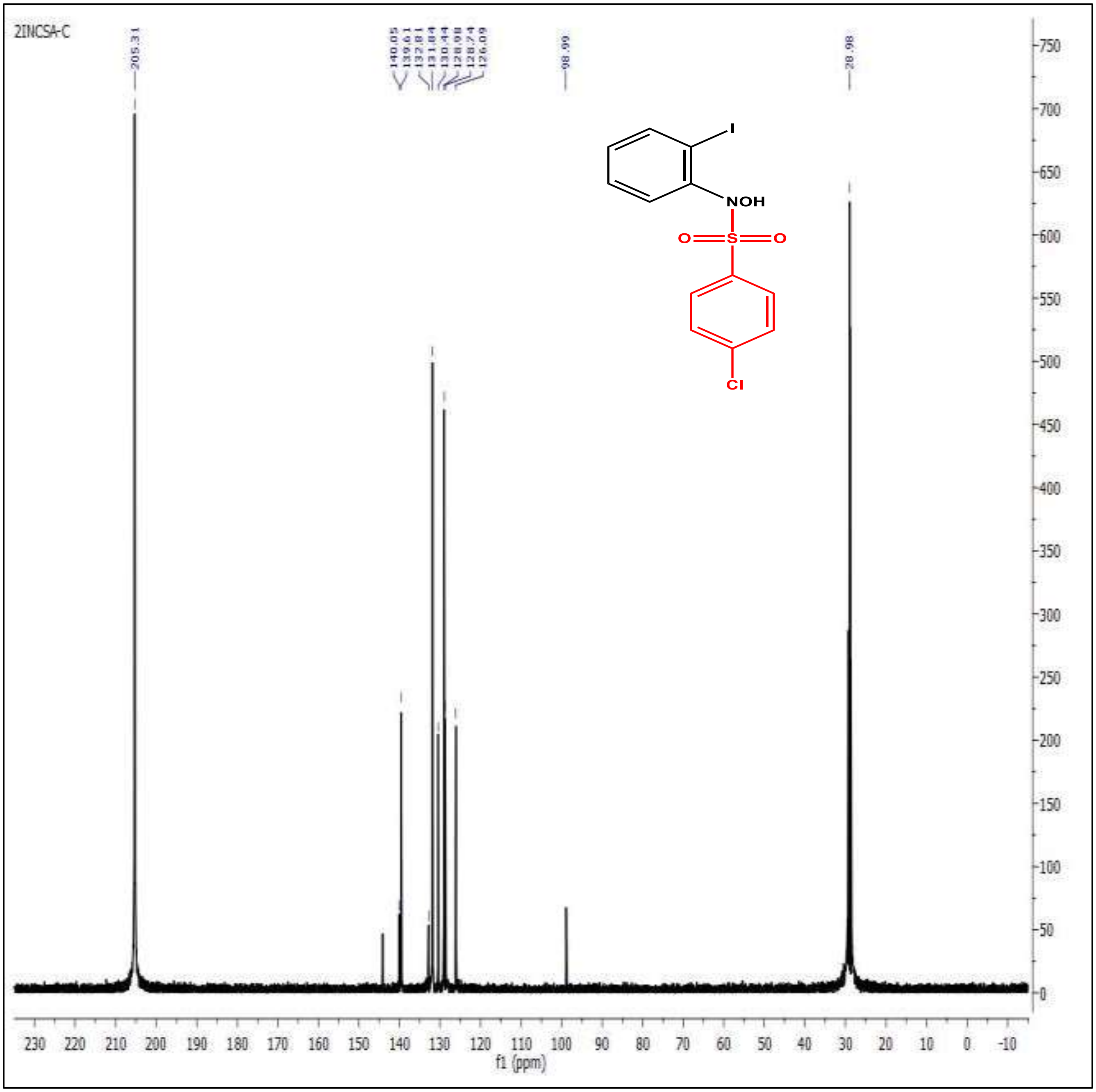

Expanded ^{13}C NMR spectrum of 3d

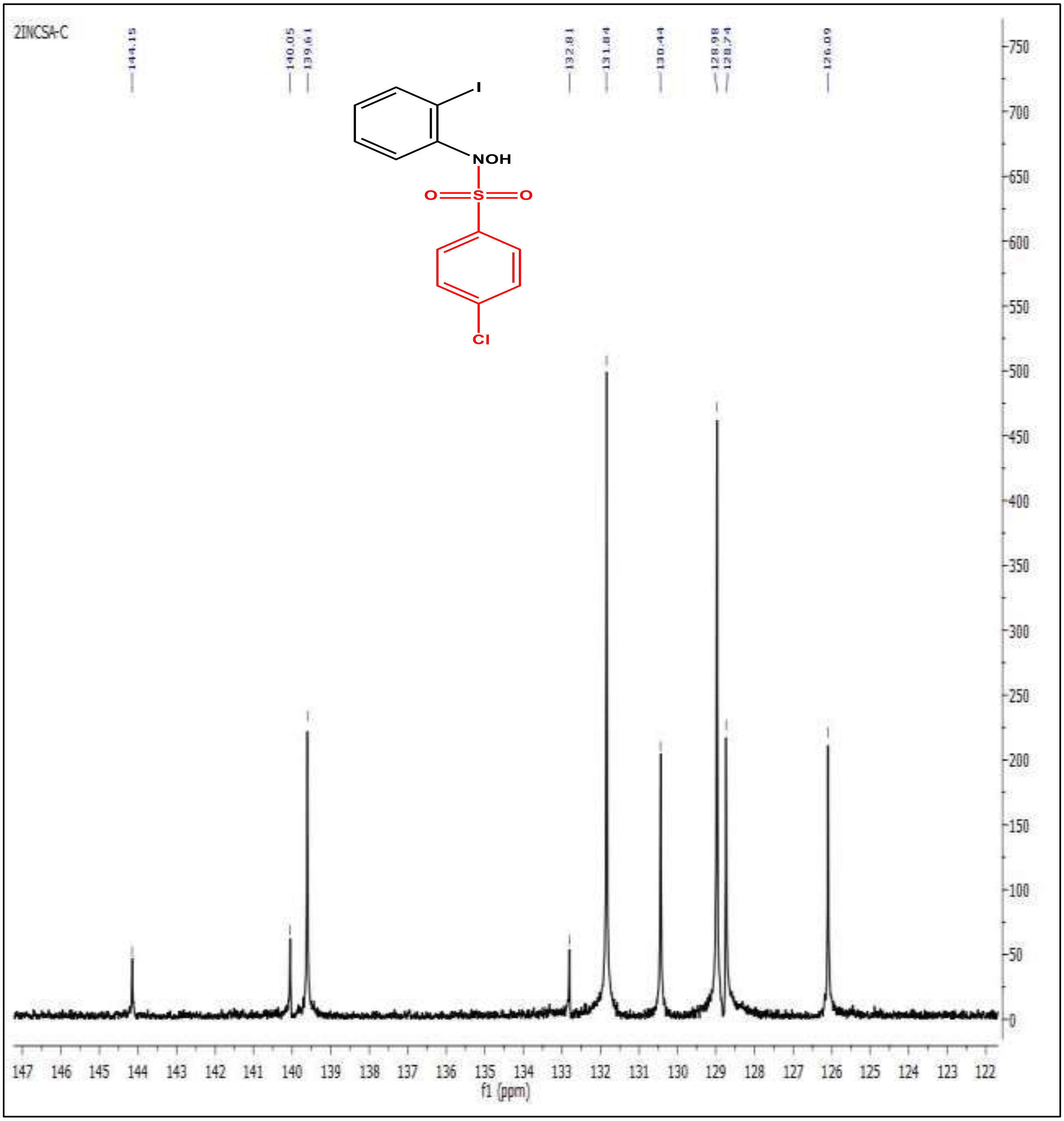

MS spectrum of 3d

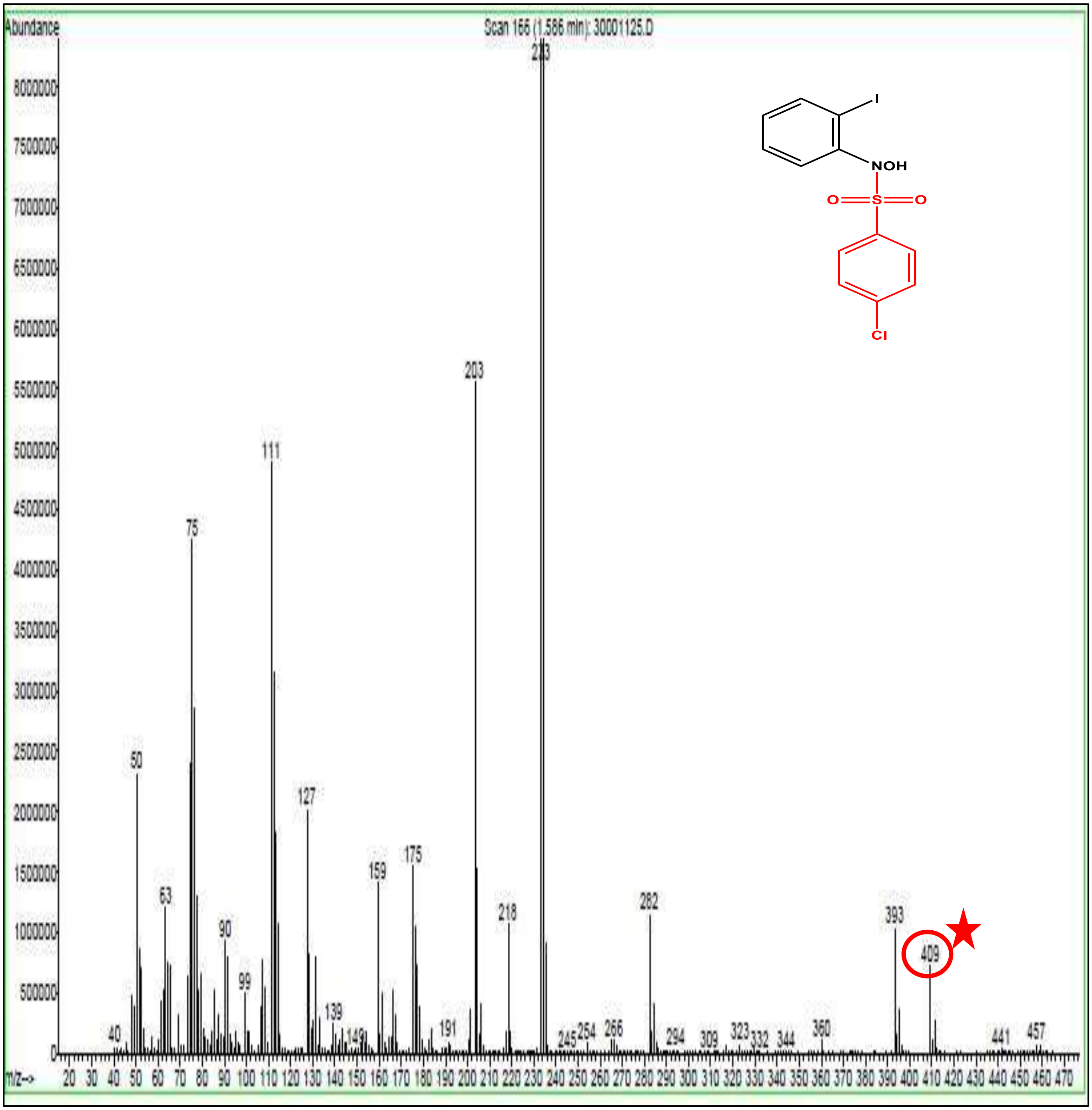

FT-IR spectrum of 1e

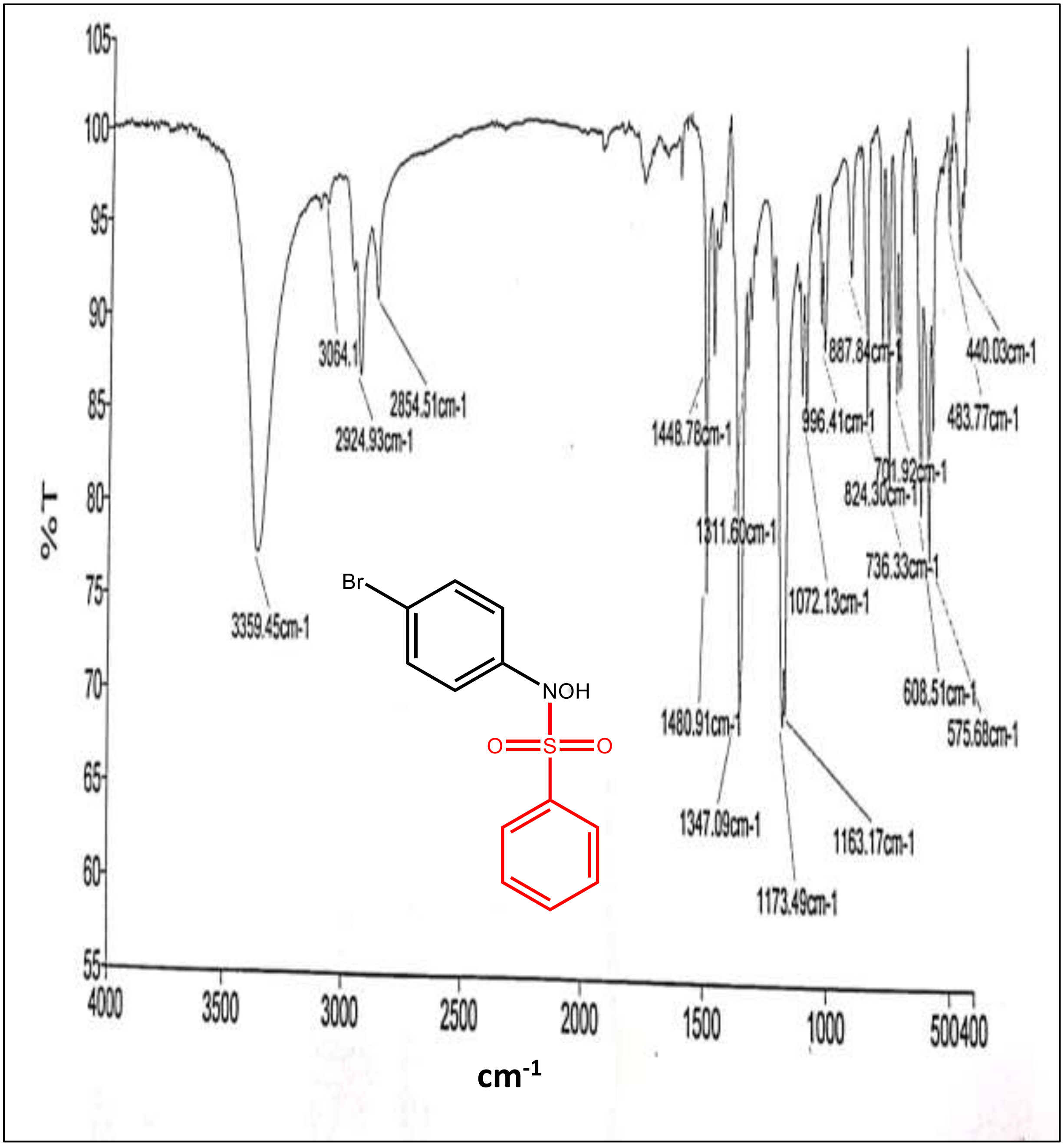

^{1}H NMR spectrum of 1e

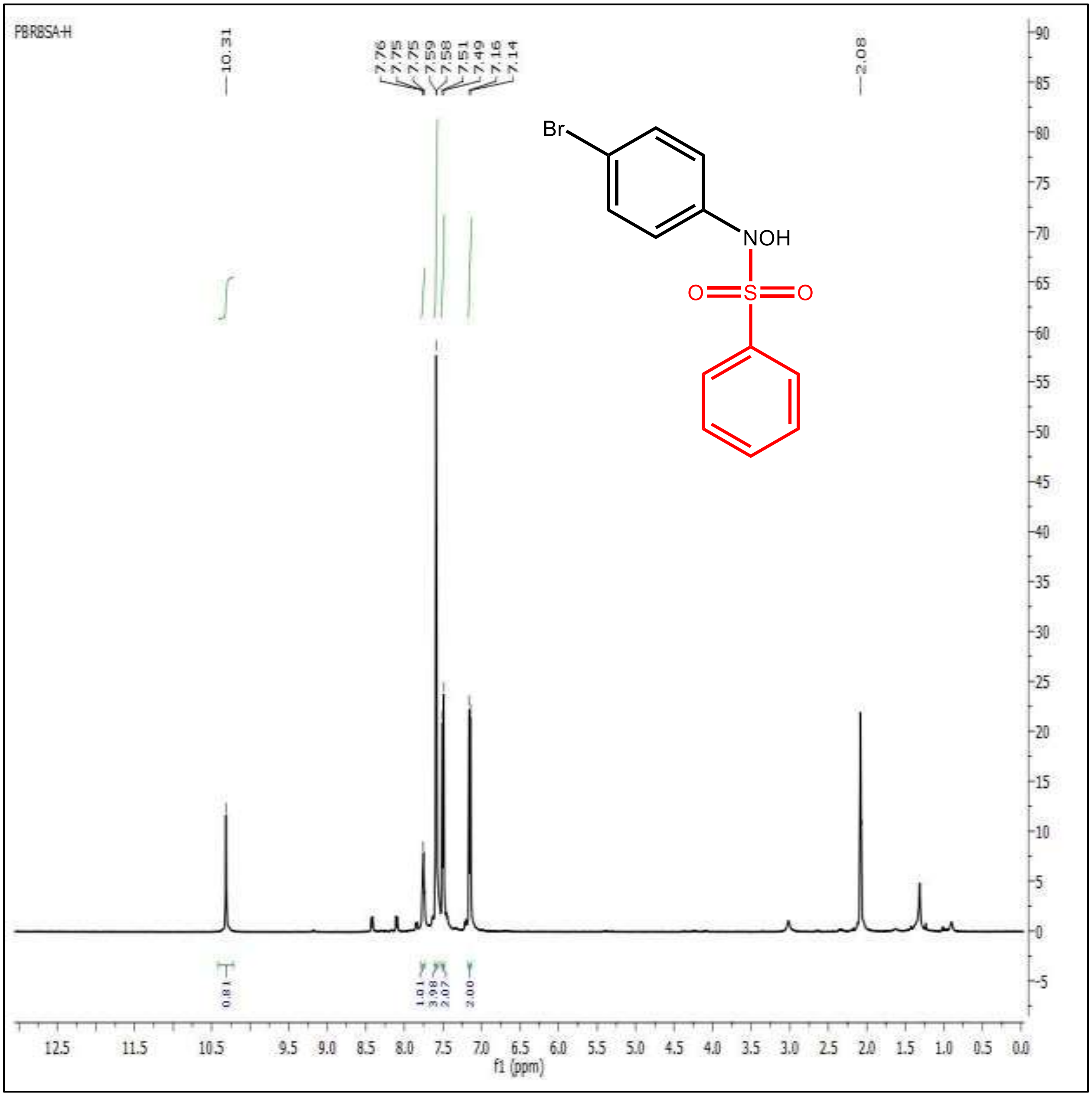

Expanded ^{1}H NMR spectrum of 1e

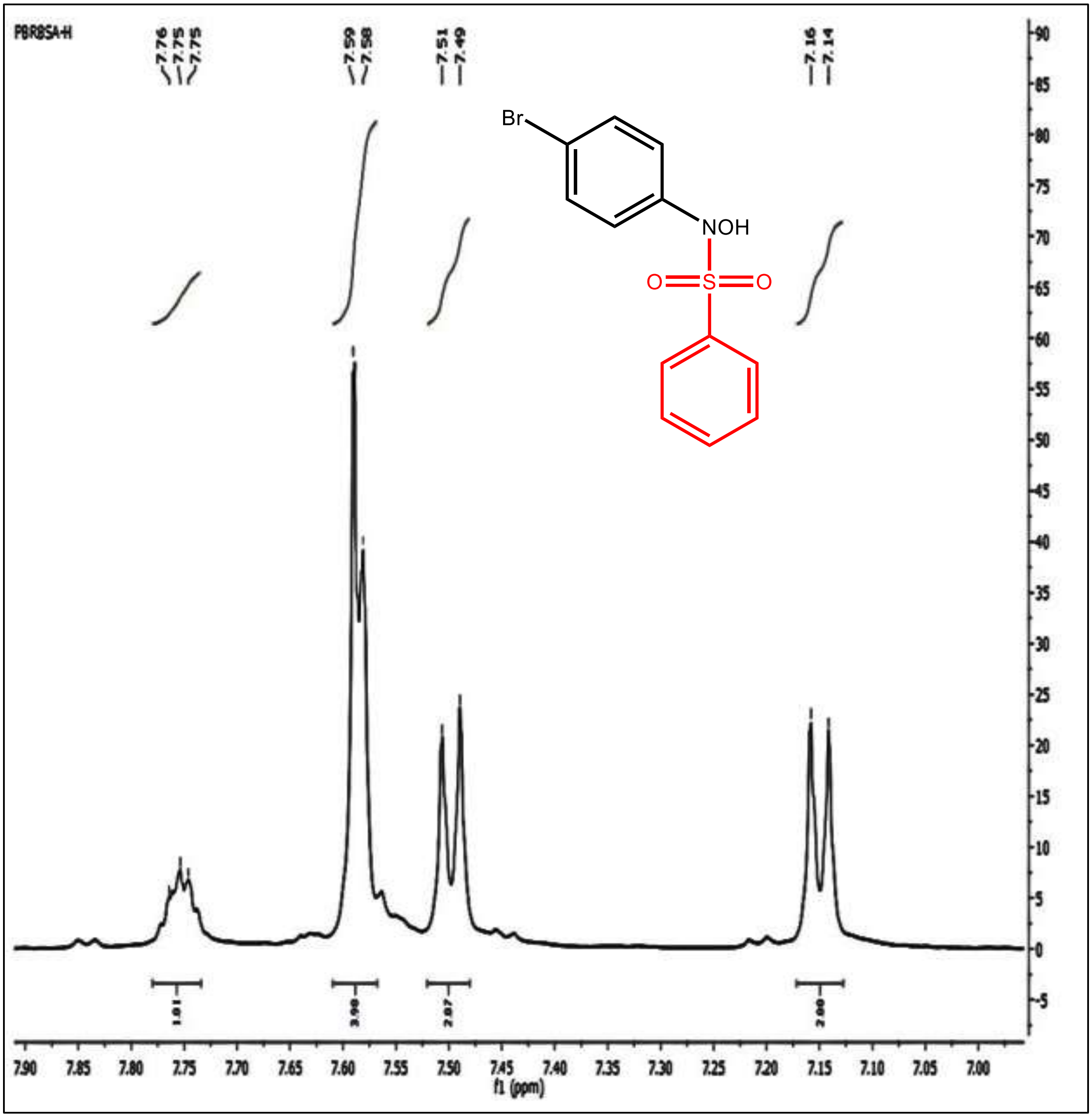

^{13}C NMR spectrum of 1e

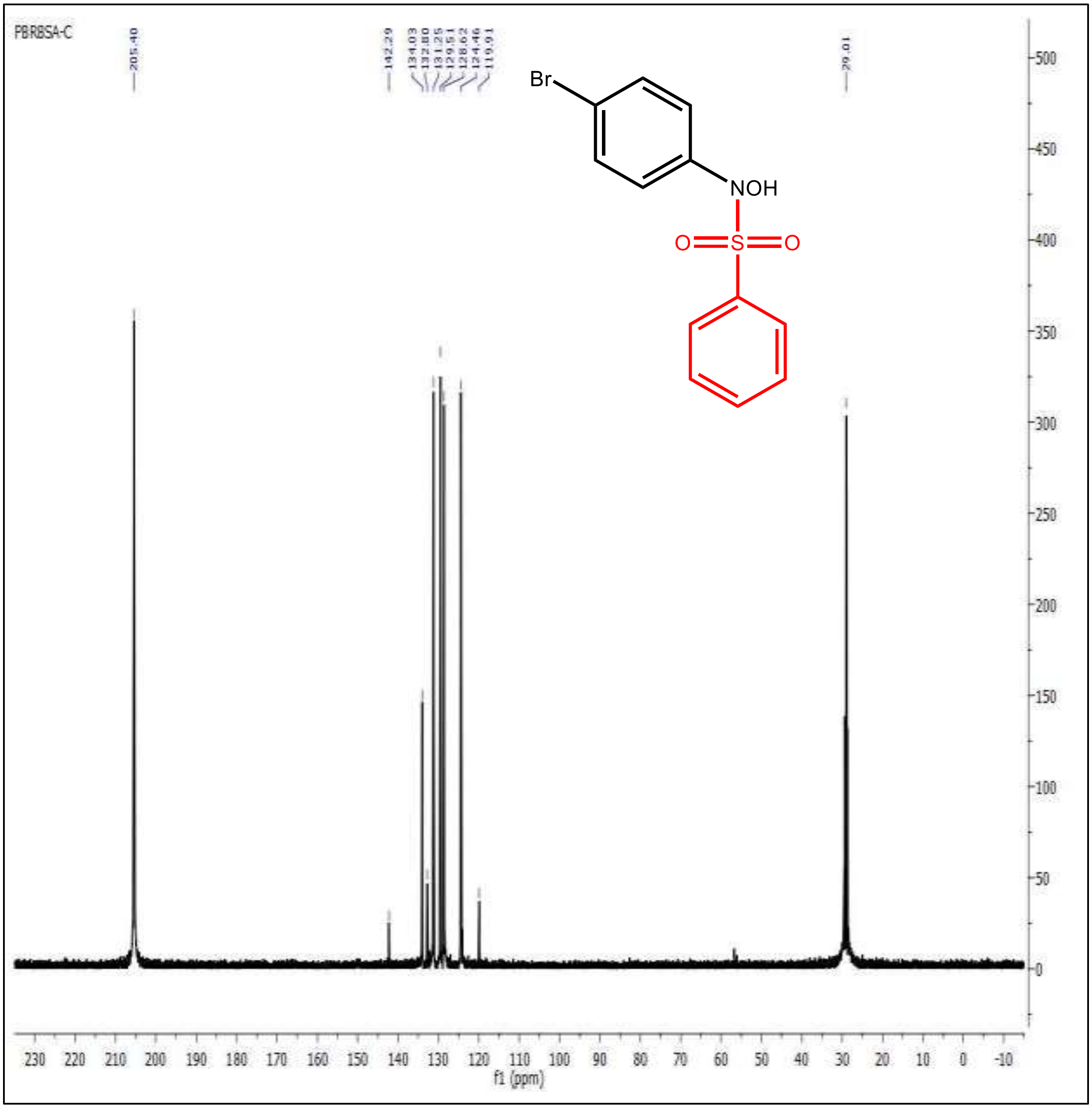

Expanded ^{13}C NMR spectrum of 1e

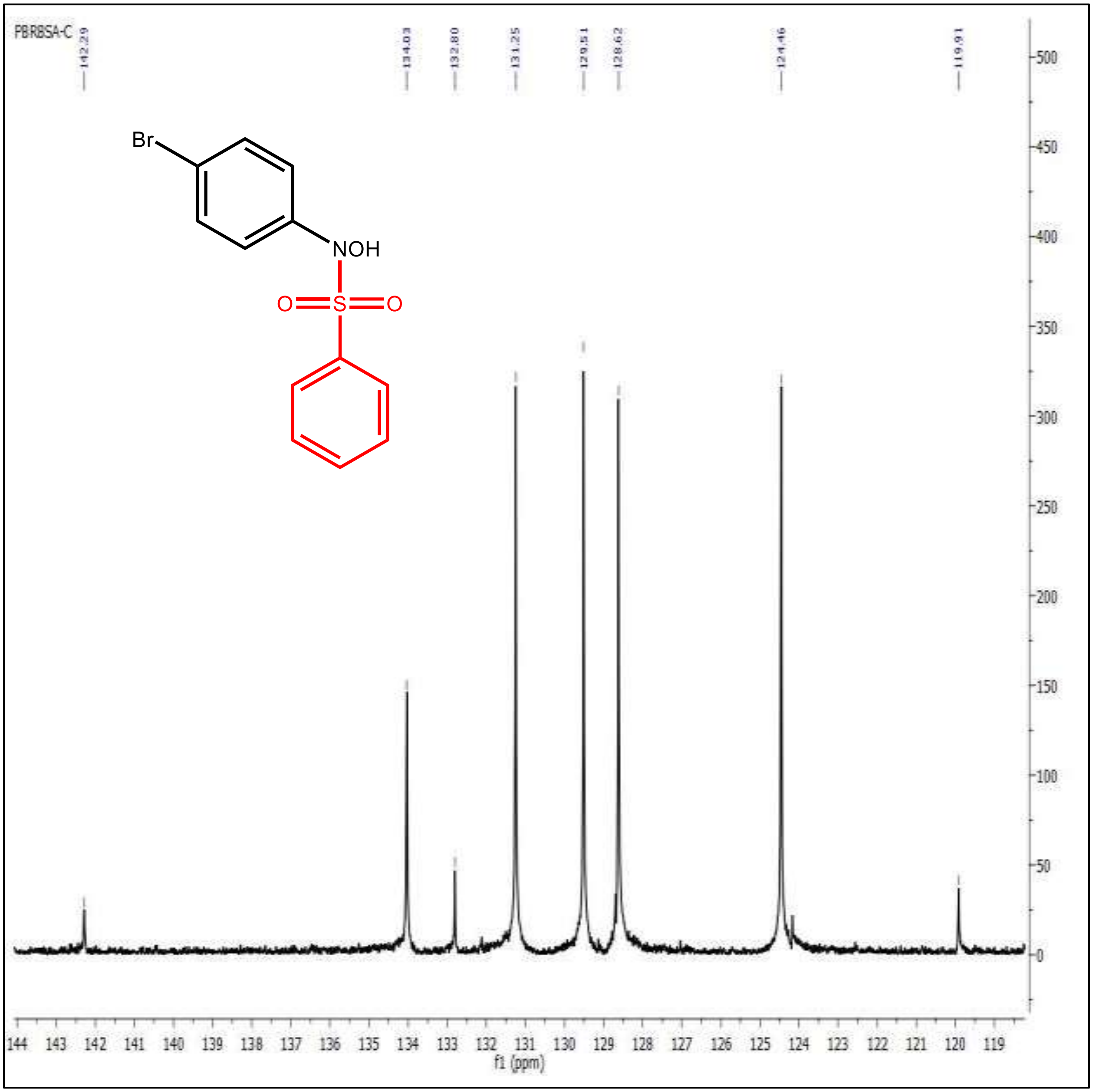

MS spectrum of 1e

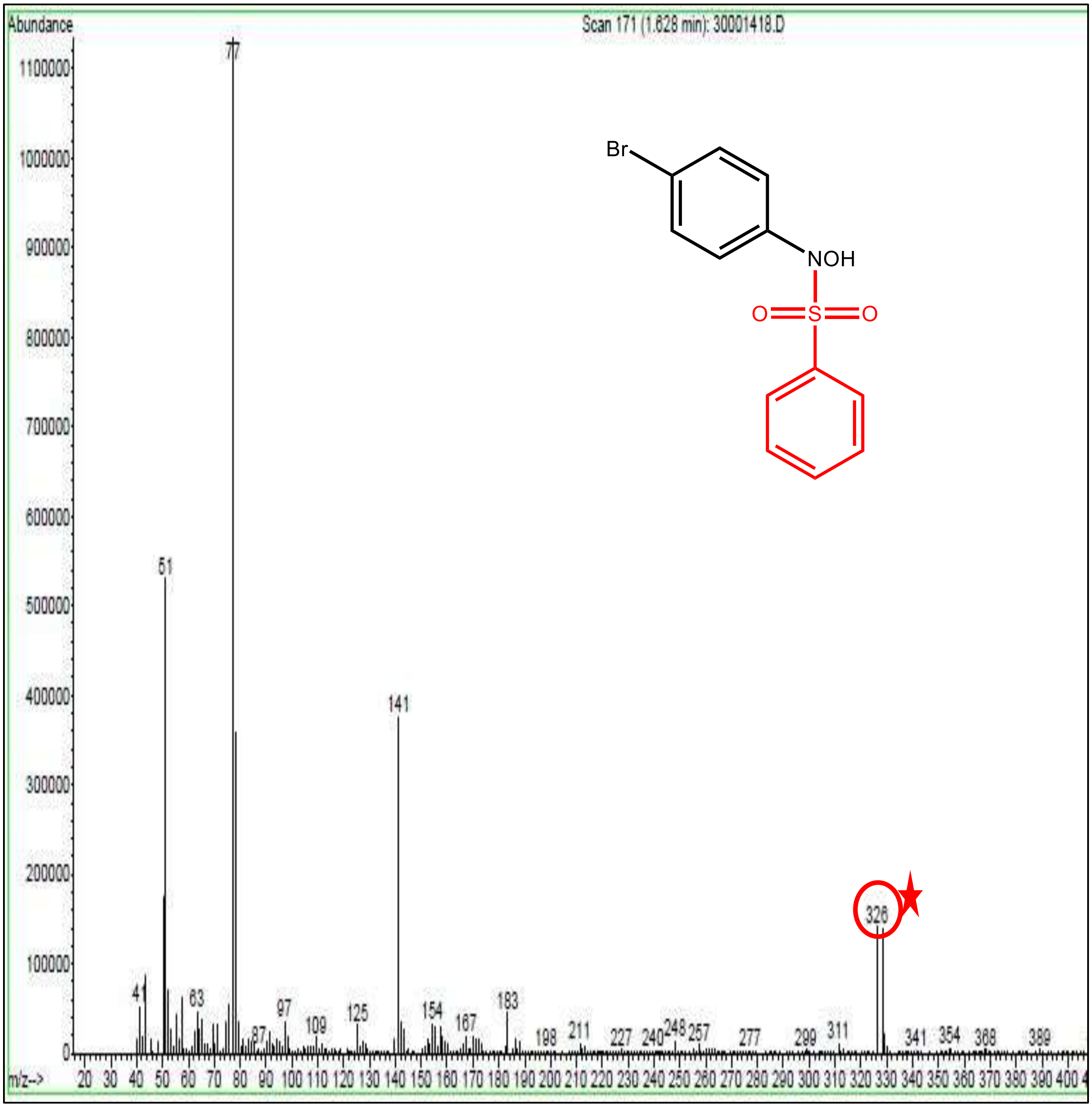

FT-IR spectrum of 2e

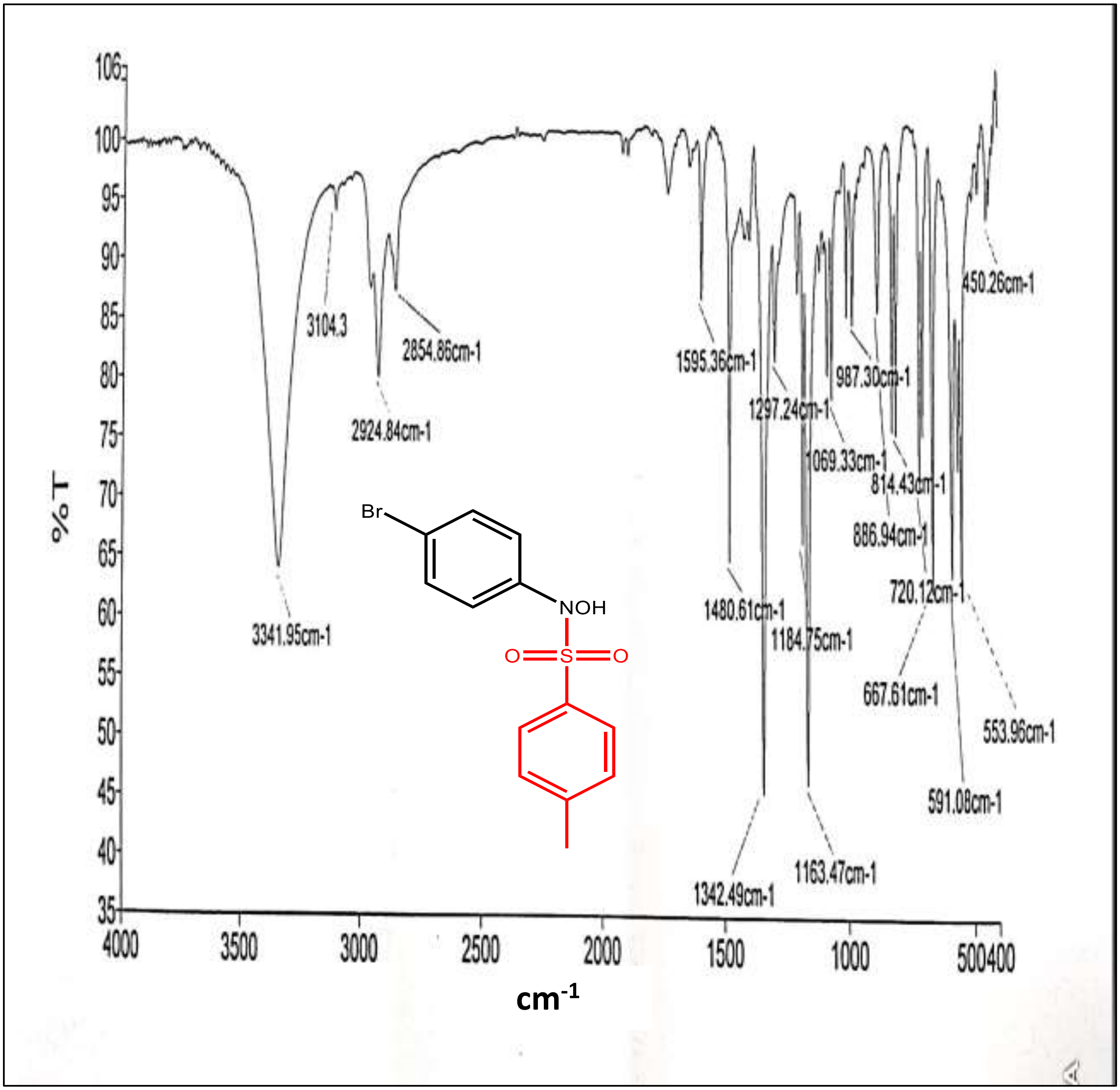

^{1}H NMR spectrum of 2e

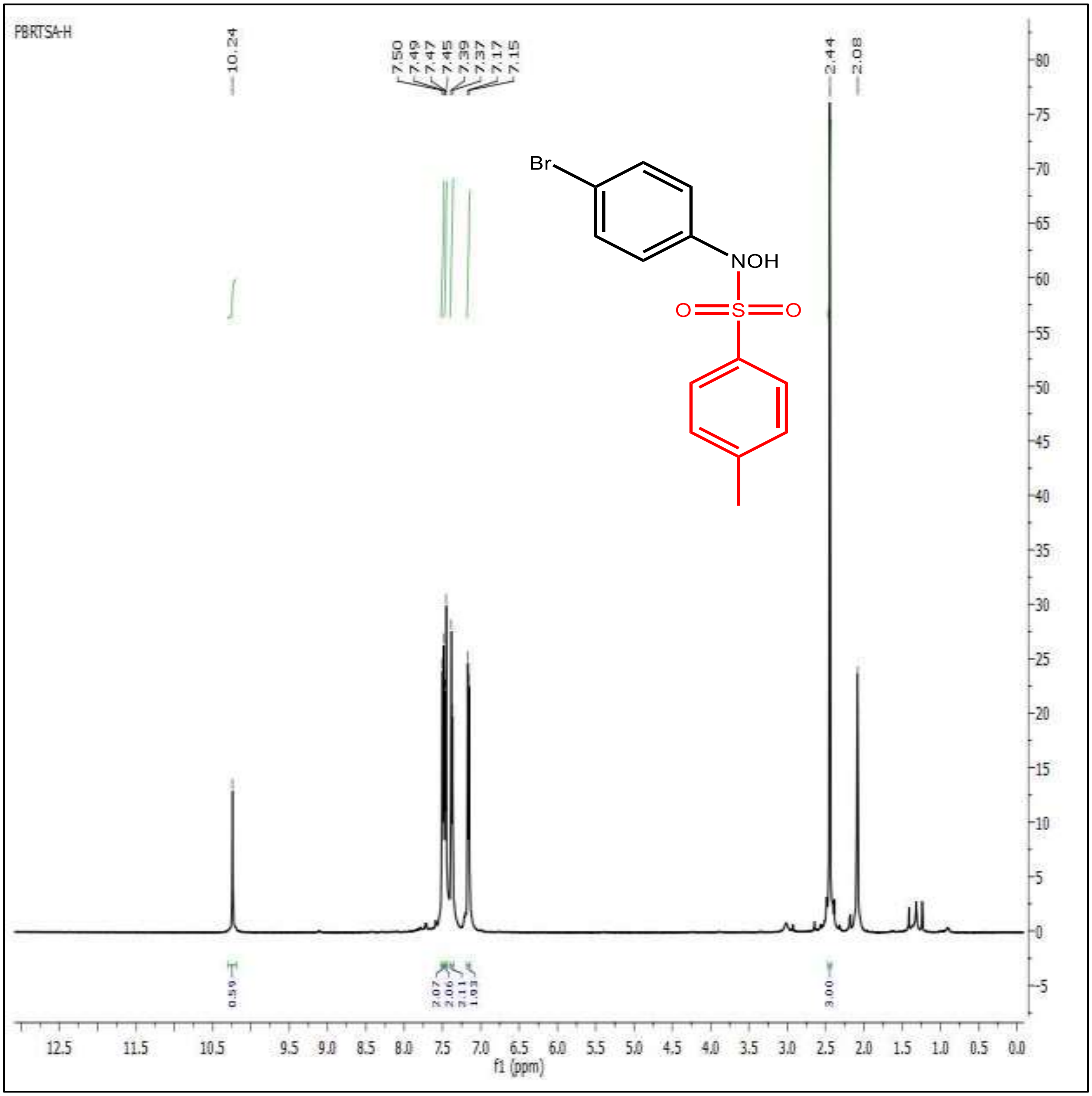

Expanded ^{1}H NMR spectrum of 2e

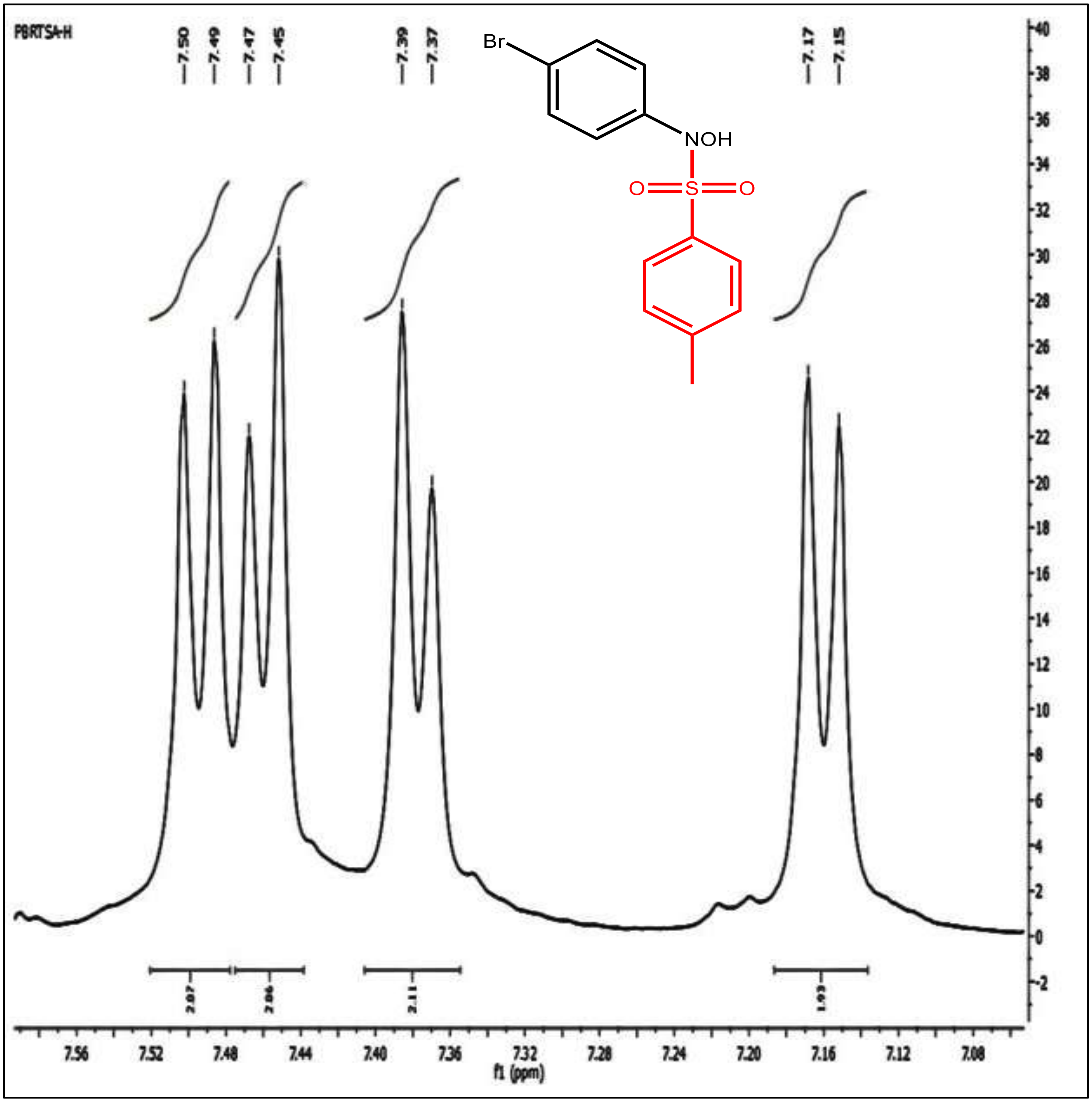

^{13}C NMR spectrum of 2e

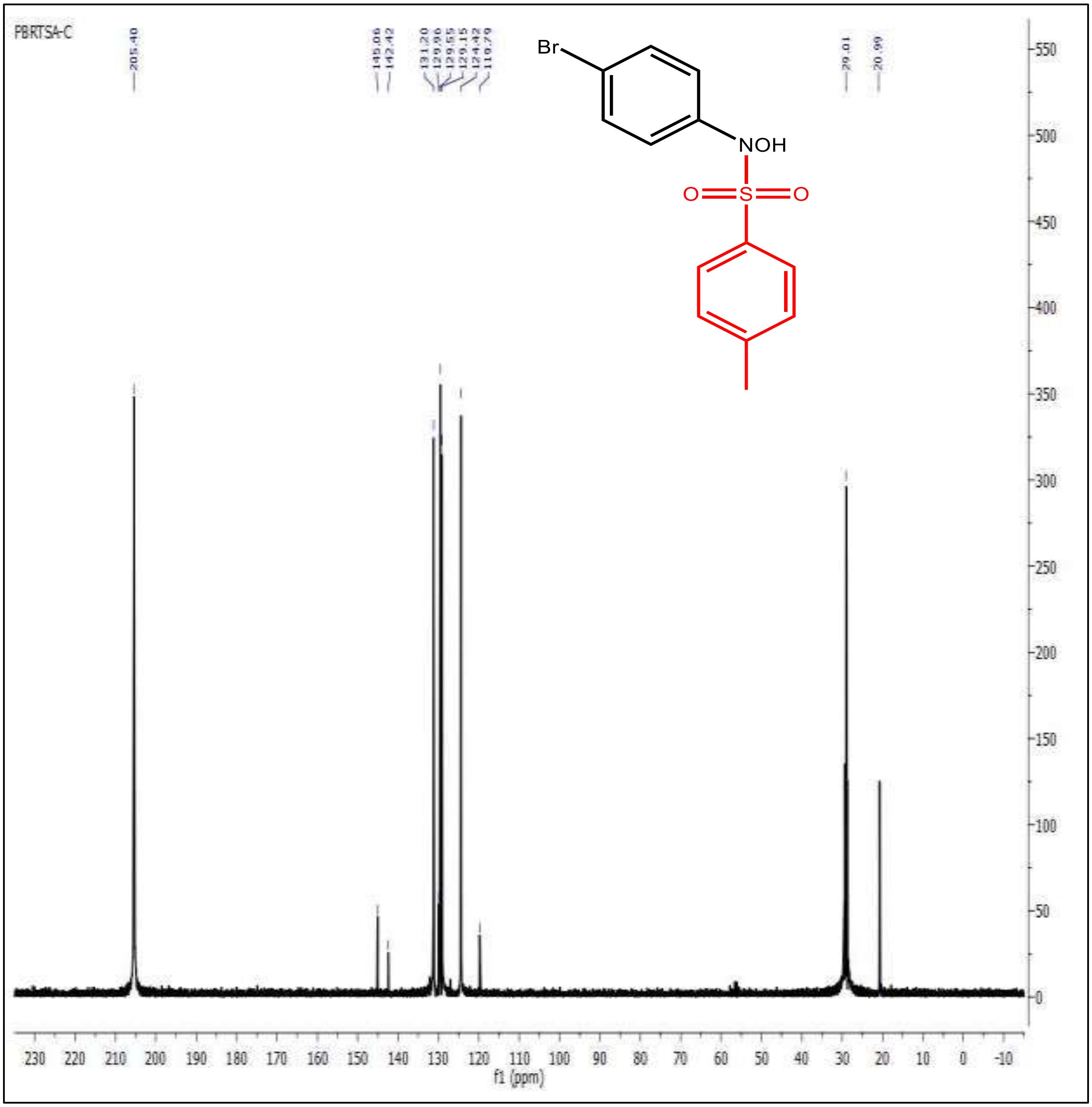

Expanded ^{13}C NMR spectrum of 2e

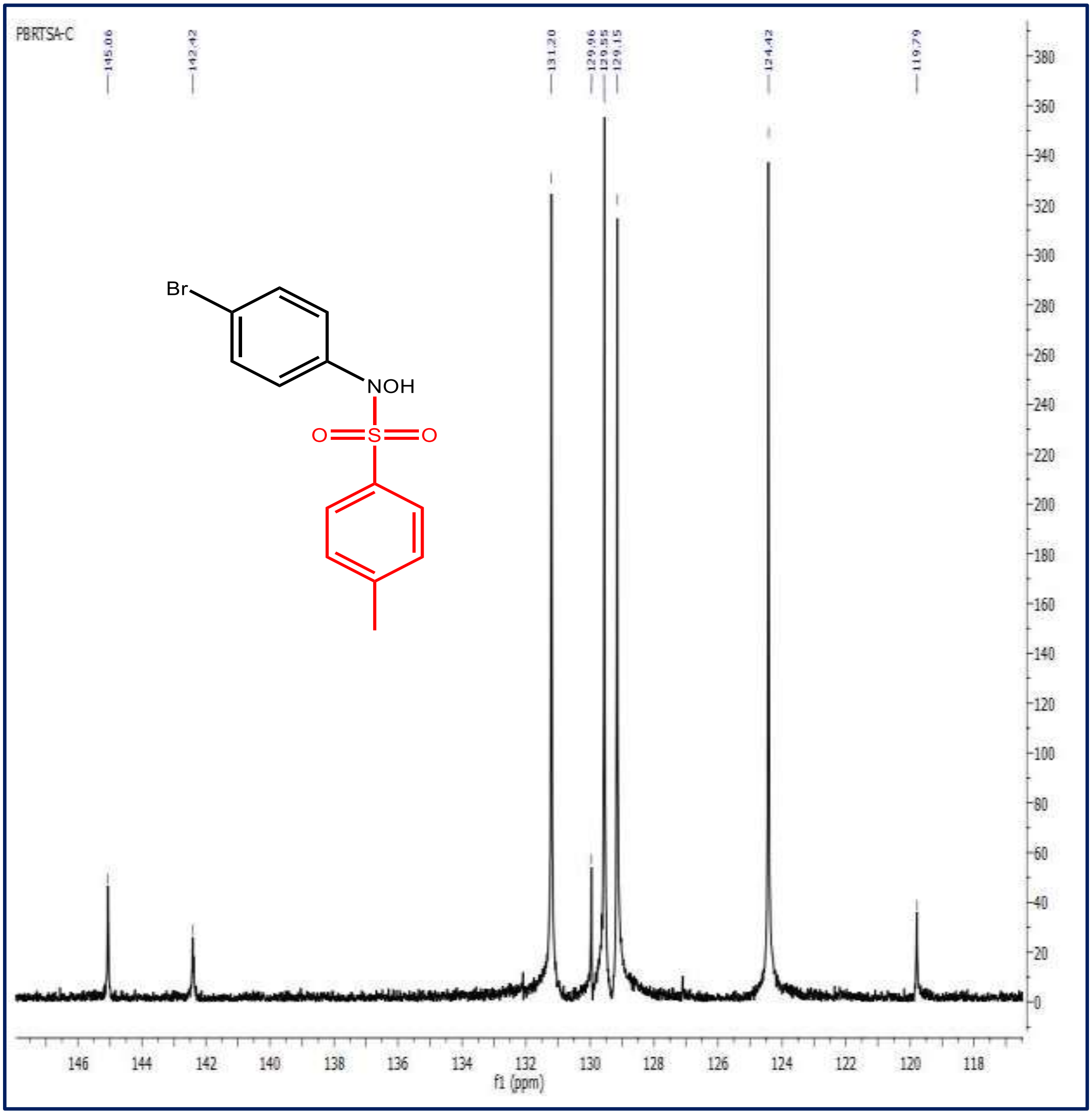

MS spectrum of 2e

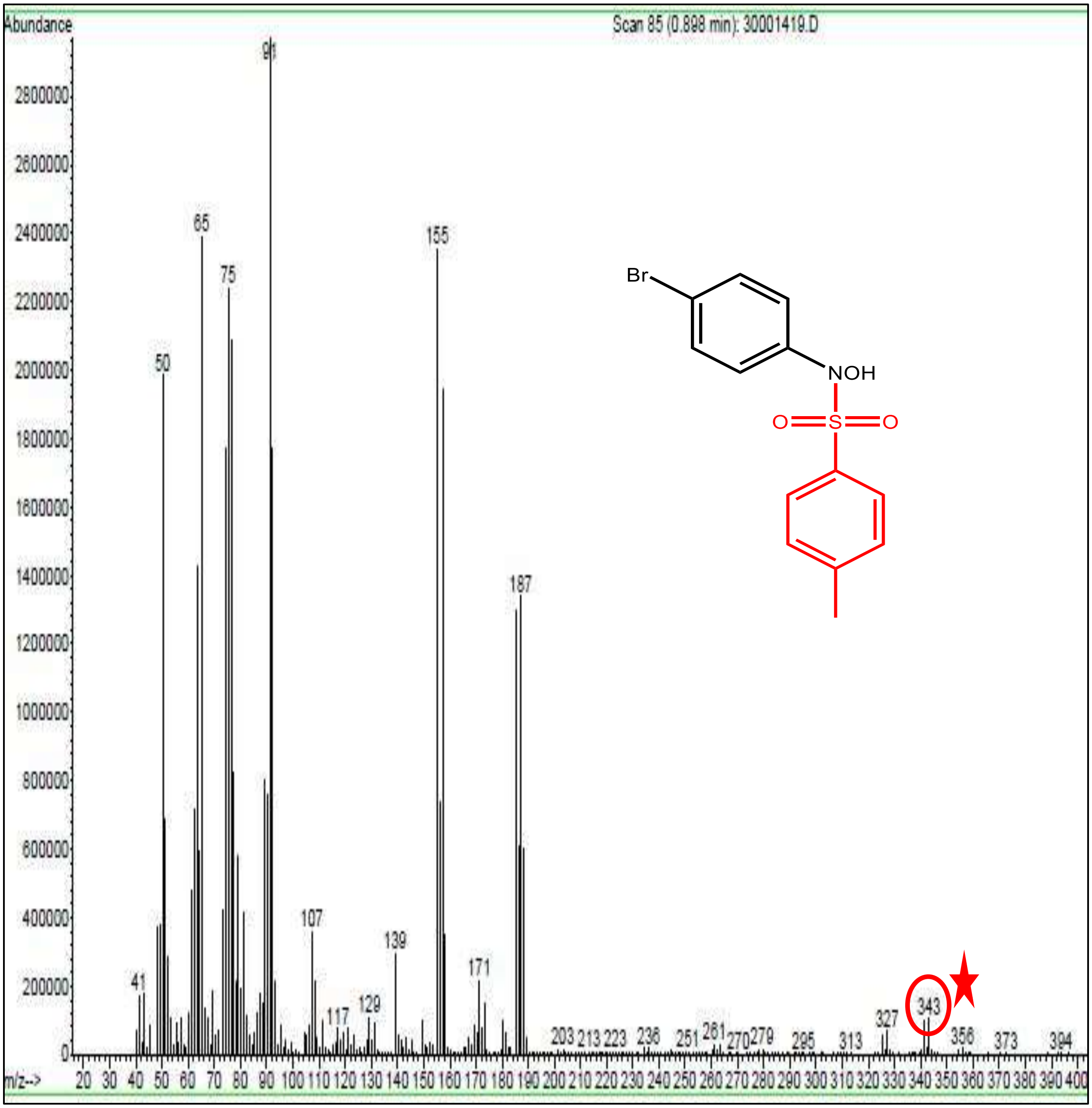

Supplement: Supplementary file 1 — Supplementary Information. [file 41598_2020_74733_MOESM1_ESM.pdf]
